# The Acetylene Bridge in Intramolecular Singlet Fission: A Boon or A Nuisance?

**DOI:** 10.1002/anie.202408615

**Published:** 2024-11-18

**Authors:** Kanad Majumder, Soham Mukherjee, Jungjin Park, Woojae Kim, Andrew J. Musser, Satish Patil

**Affiliations:** ^1^ Solid State and Structural Chemistry Unit Indian Institute of Science Bangalore 560012 India; ^2^ Department of Chemistry and Chemical Biology Cornell University Ithaca New York 14853 USA; ^3^ Department of Chemistry Yonsei University Seoul 03722 Republic of Korea

## Abstract

Various analogues of the alkylsilylacetylene group are frequently used as auxiliary groups to enhance the solubility and stability of the acene dimer core, widely used as platforms to investigate intramolecular singlet fission (iSF) mechanisms. However, while in the 2,2’‐linked dimers they are primarily auxiliary groups, these are essential fragments of the bridging units in 6,6’/5,5’‐linked dimers, the two preferred choices for dimerization. The starkly different iSF dynamics observed in the two variants raise the question of what role the acetylene bridges play. Here, we systematically designed a set of (oligo−)*para*‐phenylene bridged 2,2’‐linked pentacene dimers with an additional acetylene fragment in the bridging unit to mimic the structure of 6,6’‐linked dimers. Contrasting the results with previously reported analogous 2,2’‐linked and 6,6’‐linked pentacene dimers reveals that the acetylene bridges contribute to significant conformational freedom. This effect provides a mechanism to promote spin evolution within the triplet pair to achieve free triplets but also offers new parasitic pathways for triplet‐pair recombination, revealing that this structural motif can be both a boon and a nuisance. Additionally, our analysis reveals that these bridges directly modify the electronic states, highlighting significant pitfalls of the standard chromophore‐bridge‐chromophore framework used to design and interpret photophysics of iSF materials.

## Graphical abstract

Acetylene bridges provide a mechanism to promote spin evolution within the triplet pair to achieve free triplets but also offers new parasitic pathways for triplet‐pair recombination, revealing to be both a boon and a nuisance.

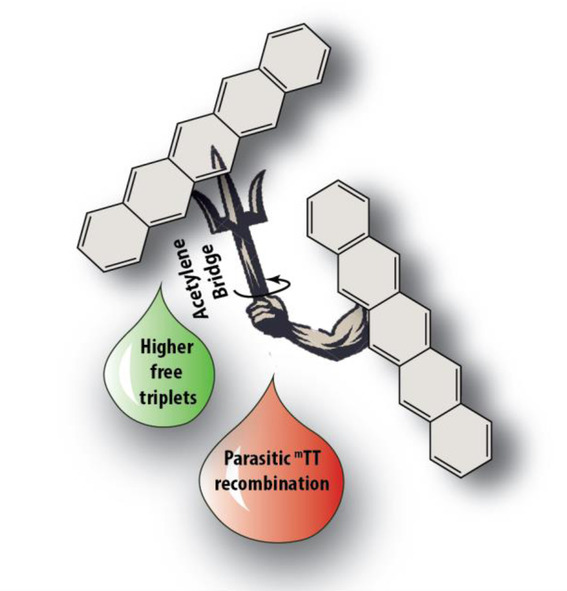

## Introduction

Since the discovery of singlet fission in the 1960s, acenes,[^1^, ^2^, ^3^, ^4^] particularly pentacene and tetracene, have been the most universally used materials to study singlet fission (SF). Although acenes present significant challenges as basic materials for application to photovoltaics and triplet harvesting because of relatively low absorption coefficients and stability issues,[^5^, ^6^, ^7^] they have still been regarded as excellent systems for mechanistic investigation for SF dynamics owing to the relative ease of spectroscopic analysis for differentiating singlet, triplet and triplet pair species.[^8^, ^9^, ^10^] Notably, because of their widespread use and tendency to exhibit SF in derivatized form, the acene systems are optimal candidates for developing molecular dimers to systematically understand intramolecular SF (iSF) mechanisms and their correlation with molecular structure. Most studies within this dimer space explore the impact of tuning the electronic nature of the linker/bridge between chromophores, for instance, by changing the orbital interactions through the mode of linkage, the length of the linker, and the nature of conjugation. Other studies also explore the effects of through‐space interactions in these systems by modulating their molecular geometry/conformation.[^8^, ^9^, ^10^, ^11^, ^12^, ^13^, ^14^, ^15^, ^16^, ^17^, ^18^, ^19^, ^20^, ^21^, ^22^] These studies build on the foundations laid down by earlier experimental and theoretical explorations of intramolecular energy/electron transfer dynamics and bring more of those crucial ideas into the iSF design space.[^23^, ^24^, ^25^, ^26^, ^27^]

Even though there are several possible carbon positions on a monomeric unit to synthesize acene dimers,[^28^, ^29^, ^30^, ^31^, ^32^, ^33^] generally two linking modes, one along the long axis and the other along the short axis of the acene monomer, have been utilized. Representatively, in pentacenes, these are 6.6’‐linked pentacene dimers (short‐axis coupled) and 2,2’‐linked pentacene dimers (long‐axis coupled).[^9^, ^10^] The acene monomers are synthesized with additional alkylsilylacetyelene groups along the short axis due to synthetic feasibility but also to enhance solubility and provide protection against oxidation and decomposition of the acene core.^[34]^ Consequently, one of the structural differences between the short‐axis and long‐axis coupled dimers is the presence of this acetylene bridge along the coupling axis, which makes the acetylene bridges inherently important to the iSF dynamics. A large body of the vast acene literature deals with correlating iSF dynamics with structural variations within these two classes, with little study of the fundamental difference between the two.^[18]^ Specifically, the 6,6’‐linked dimers show ultrafast singlet fission but simultaneously substantial ^m^TT (m=1,3,5) recombination which is the predominant decay pathway in many of these systems, shorter free triplet lifetimes, and reduced triplet yields. The faster iSF in these dimers is often correlated to the presence of energetically accessible charge transfer (CT) states to mediate iSF in these dimers. The acetylene bridges additionally allow the 6,6’‐pentacene dimers to access a large pool of rotational conformers leading to heterogeneity in their iSF behaviour.[^35^, ^36^, ^37^, ^38^] Contrastingly, the 2,2’‐linked dimers show relatively slower iSF but longer TT lifetimes, and correspondingly higher triplet yields.[^9^, ^10^, ^17^, ^18^] A number of these 2,2’‐linked dimers exhibit the direct iSF mechanism due to energetically inaccessible CT states, or in other words, minimal CT character in the lowest exciton.[^29^, ^39^, ^40^] Despite the highly contrasting iSF behaviors of the 2,2’‐ and 6,6’‐dimers, a detailed understanding of the origin of their differences has remained elusive.[^9^, ^10^, ^17^] Furthermore, comparing the two structural variants raises an important question regarding the perceived modularity of iSF dimers, in which the SF chromophores are commonly treated as distinct from the bridging units. Resolving this question could provide a convenient basis for analyzing structure–property relationships in iSF dynamics of various dimeric systems. Since the acetylene bridges are chiefly used in the pentacene dimers, it is of prime importance to acknowledge their effect on iSF and identify whether they merely act as bridging units or provide richer functional effects.

**Figure 1:**
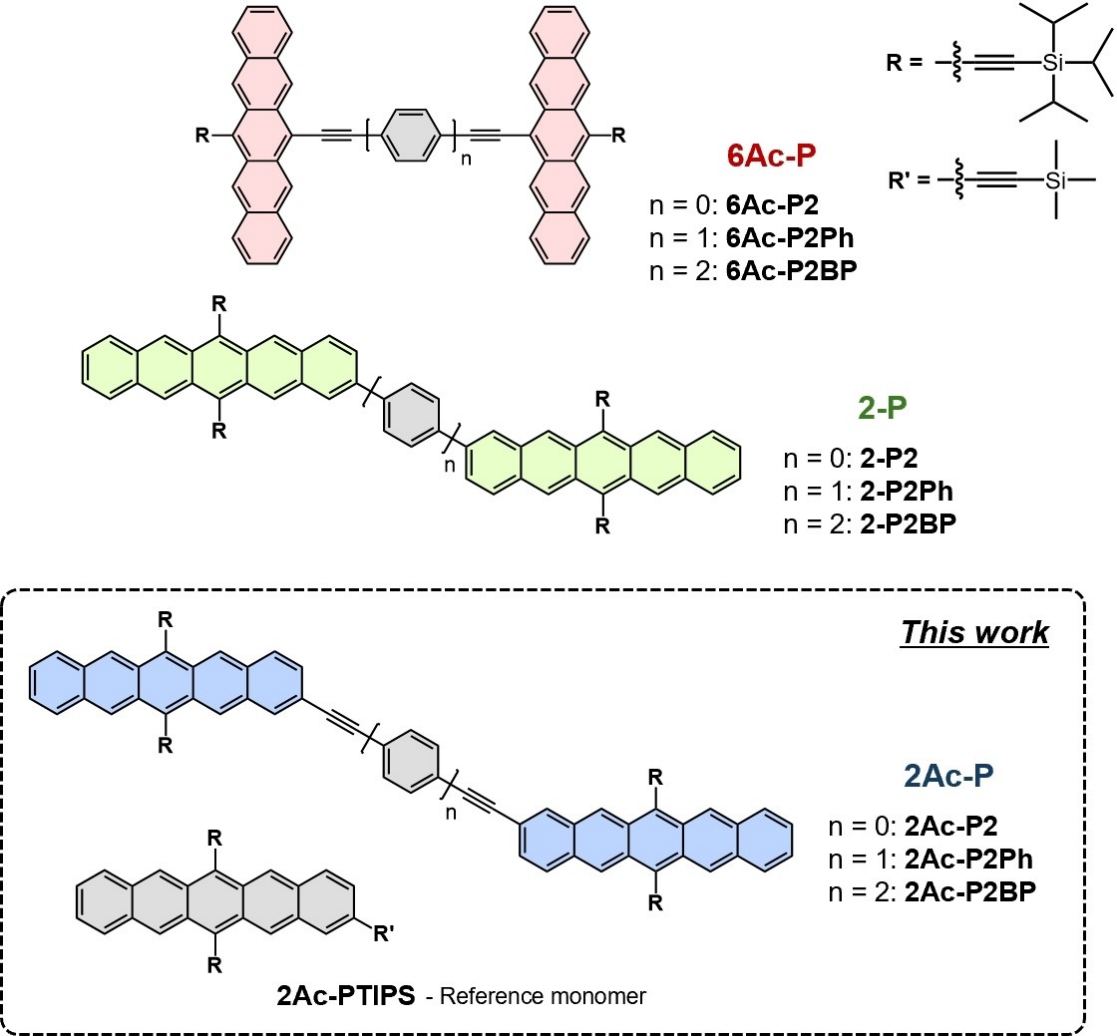
Schematic of the three pentacene dimer classes studied. n indicates the number of *para*‐phenyl rings in the oligophenylene bridges.

Given that the acetylene bridges are known to provide rotational flexibility in the 6,6’‐linked pentacene dimer systems, it urges one to question their role in the contrasting behaviour of the two dimer classes and whether this role provides synthetic scope for future molecular design.[^35^, ^36^, ^37^, ^38^] To investigate systematically the role of the acetylene bridge in SF and understand the origin of the contrasting iSF behavior between the 2.2’‐ and 6,6’‐linked dimers, we present in this work the SF dynamics of a 2,2’‐linked pentacene dimer series with acetylene bridge to mimic the structure of the 6,6’‐linked dimers (Figure 1). We establish that the acetylene bridges provide structural flexibility to the systems that modulate iSF significantly, even though these may not be apparent from the steady‐state absorption in all dimers. Notably, these motifs are important in strongly coupled dimers to facilitate free triplet generation through fluctuating exchange couplings. However, because the acetylene bridges also allow access to planar geometries, they often provide parasitic fast recombination pathways from the TT states. The acetylene bridges may be a boon or a nuisance, depending on the intended application. Our observations also highlight shortcomings of the usually employed chromophore‐bridge‐chromophore basis and the challenges that emerge for extracting broader design principles across diverse dimer structures.

## Results and Discussion

**Three Classes of Pentacene Dimers**. To disentangle the effects on iSF of the acetylene bridges, inter‐pentacene distances, and linking geometry, we directly compare three series of pentacene dimers: **6Ac‐P**, **2‐P**, and **2Ac‐P** with (oligo−)*para*‐phenylene linkers as shown in Figure 1. The **6Ac‐P** and **2‐P** series are established models for 6,6’‐linked and 2,2’‐linked pentacene dimers.[^9^, ^10^, ^37^] Here, we introduce a new series of 2,2’‐linked TIPS‐pentacene dimers, **2Ac‐P**, with additional acetylene linkages to mimic the bridge structure of the **6Ac‐P** series.^[41]^ The synthesis of 2,2’‐linked TIPS‐pentacene dimers with (oligo−)*para*‐phenylene linker was carried out via a carbon‐carbon Sonogashira coupling reaction between the respective bromo derivative of TIPS‐pentacene **1** and the diethynyl substituted (oligo−)*para*‐phenylene linker in the presence of palladium catalyst Pd(PPh_3_)_4_ and copper chloride in tetrahydrofuran (THF), toluene 3 : 1 mixture and diisopropylamine (DIPA) mixture to get the desired pentacene dimer **2Ac‐P2Ph**/**2Ac‐P2BP**. Synthesis of the 2,2’‐linked TIPS‐pentacene dimer without any (oligo−)*para*‐phenylene spacer was carried out via a homocoupling reaction of the ethynyl substituted TIPS‐pentacene **2** in the presence of *N*,*N*,*N*’,*N*’‐tetramethylethylenediamine (TMEDA) and copper chloride in dry dichloromethane (DCM) at room temperature to get the desired pentacene dimer **2Ac‐P2**. According to the previously reported procedures, **6Ac‐P** and **2‐P** dimers were also synthesized[^9^, ^10^] for comparison with **2Ac‐P** dimers. Detailed synthetic procedures and basic characterization results are shown in the Supporting Information Section 2.

**Figure 2:**
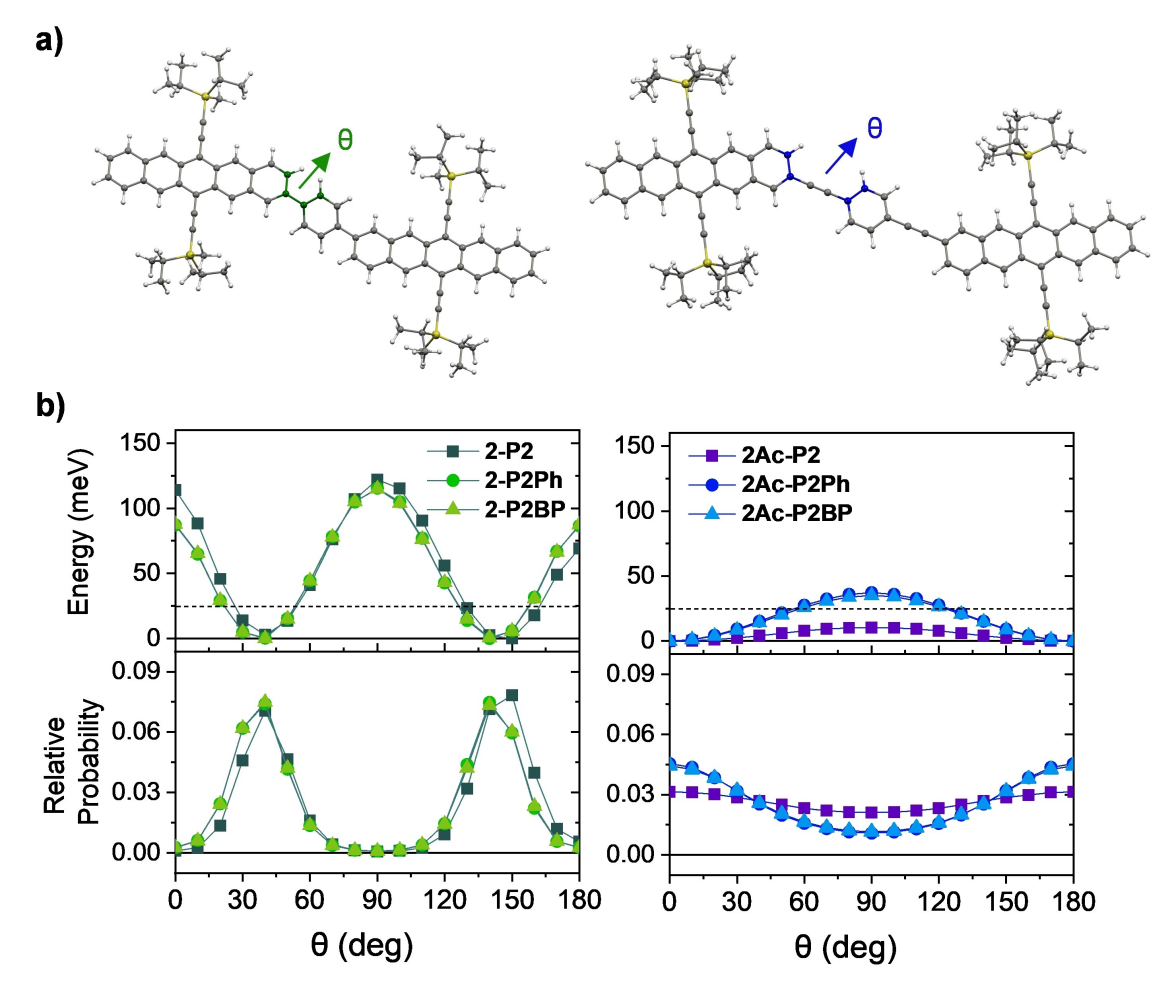
Energy‐minimized structures of (a) **2‐P2Ph** (left) and **2Ac‐P2Ph** (right) calculated at the CAM‐B3LYP/6‐31g(d) level of theory. θ indicates dihedral angles along the pentacene‐phenyl axis (or pentacene‐pentacene axis in the case of **2‐P2** and **2Ac‐P2**. (b) Calculated potential energy curves (top) and relative probabilities (bottom) along the θ coordinate in the ground state of **2‐P** dimers (left) and **2Ac‐P** dimers (right). The horizontal dashed lines in the top panels denote the room temperature energy.

**Molecular Structures**. We performed DFT calculations to assess how acetylene linkers influence the ground‐state structural distributions in the different dimer classes. We focused primarily on comparing the structures of **2‐P** and **2Ac‐P** dimers; a similar analysis of the **6Ac‐P** structures can be found in our previous work.^[35]^ The optimized geometrical parameters for all dimers are detailed in Table S3.1.1. The energy‐optimized structures of **2‐P** dimers exhibit a dihedral angle (θ) ranging from 37° to 40° between the pentacene planes and the adjacent linker ring, or the adjacent pentacene in the case of **2‐P2**. Additionally, **2‐P** dimers display a steep torsional potential due to significant steric hindrance caused by the *ortho* hydrogens of adjacent *para*‐phenyl rings (Figure 2b, left). In contrast, **2Ac‐P** dimers present entirely planar structures between pentacene and *para*‐phenyl planes in their energy‐minimized configurations, thereby maximizing π‐conjugation. However, the acetylene bond in these dimers leads to conformational heterogeneity due to a shallow torsional barrier, allowing all rotational conformers to coexist at room temperature.[^35^, ^38^, ^42^] Notably, the absence of a phenyl ring in **2Ac‐P2** results in a markedly shallower potential energy curve and a lower rotational barrier along the acetylene bridge (Figure 2b, right), especially compared to **2Ac‐P2Ph** and **2Ac‐P2BP**. **6Ac‐P** dimers, as illustrated in Figure S3.1.2, resemble **2Ac‐P** dimers in terms of ground‐state planarity and the low torsional barrier along the acetylene bridge.

Moreover, the additional acetylene bridge in **2Ac‐P** and **6Ac‐P** dimers results in inherently larger inter‐pentacene distances than their **2‐P** counterparts. It is important to note that dimers featuring a biphenyl spacer introduce an additional rotational axis. However, rotation along this axis is constrained in the ground state, potentially disrupting the conjugative interactions between pentacene chromophores in dimers with a biphenyl linkage, particularly in the otherwise planar **2Ac‐P** and **6Ac‐P** dimers.

**Figure 3:**
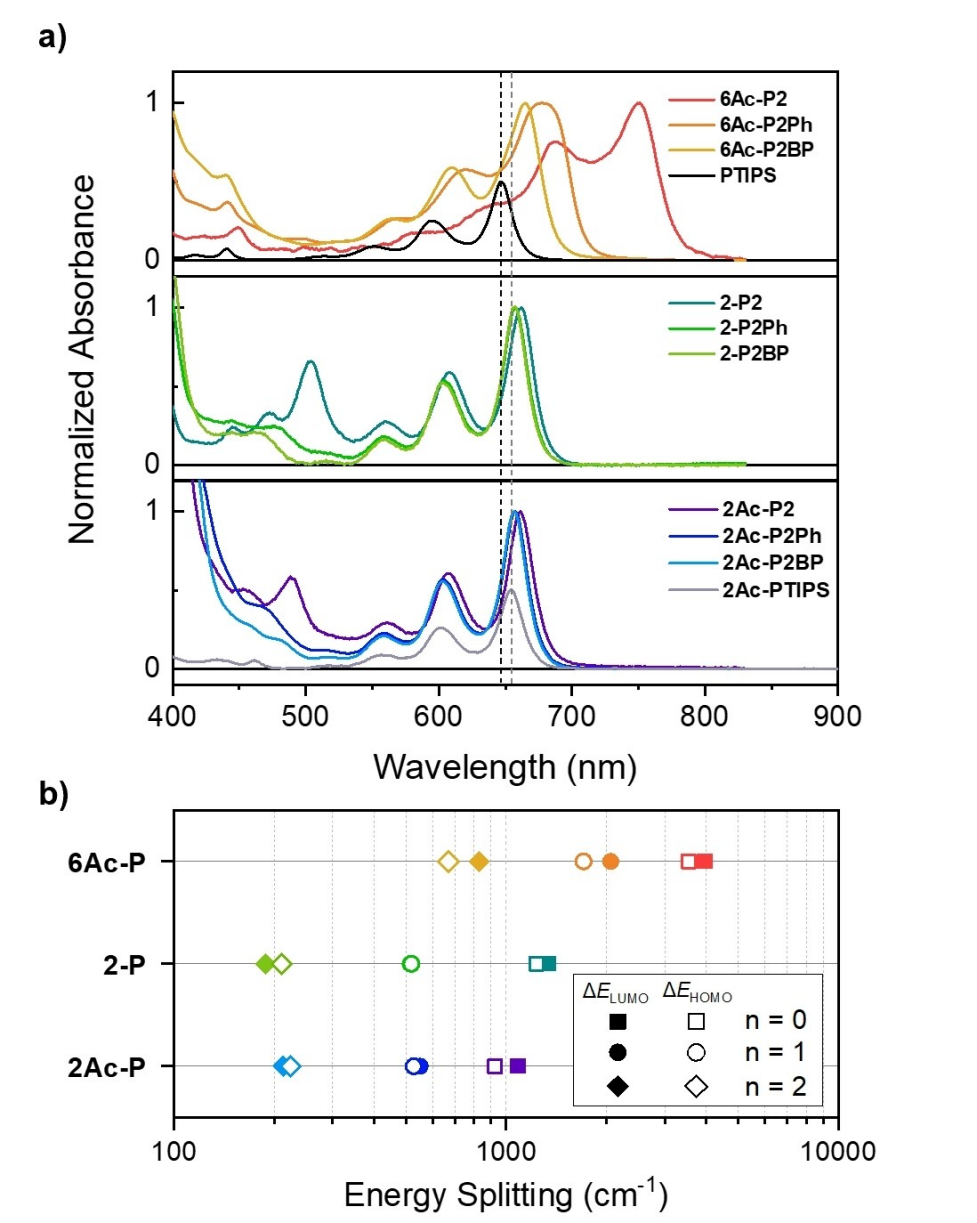
(a) Normalized absorption spectra of pentacene dimers in dilute chlorobenzene solution (c ~10^−5^ M). The absorption spectrum of **PTIPS** (top panel) and **2Ac‐PTIPS** (bottom panel) normalized to 0.5 is also shown for comparison. The black and grey vertical dashed lines indicate the peak positions of the 0–0 absorption band of PTIPS and 2Ac‐PTIPS, respectively. **2‐P2Ph** and **2‐P2BP** (middle panel), and **2Ac‐P2Ph** and **2Ac‐P2BP** (bottom panel) show identical absorption above 550 nm (b) Energy‐level splitting between LUMO+1 and LUMO (Δ*E*_LUMO_) and HOMO and HOMO‐1 (Δ*E*_HOMO_) in **6Ac‐P**, **2‐P**, and **2Ac‐P** dimers.

**Steady‐State Optical Characteristics and Inter‐pentacene Electronic Coupling**. Figure 3 presents a comparative analysis of the normalized absorption in dilute chlorobenzene solution of **6Ac‐P**, **2‐P**, and the newly synthesized **2Ac‐P**. Relative to the reference monomers (PTIPS and 2Ac‐PTIPS) (See Supporting Information Section 2), all dimers exhibit redshifts of the S_0_→S_1_ transition. The magnitude of these shifts is less pronounced when additional *para*‐phenyl spacers, which disrupt inter‐pentacene electronic interactions, are incorporated between the pentacene chromophores. Notably, the **6Ac‐P** dimers display the most significant redshifts, with values up to approximately 265 meV in **6Ac‐P2**. This shift is attributed to the parallel alignment of the conjugation axis and S_0_→S_1_ transition dipole moments resulting in a head‐to‐tail orientation of the dipoles and a strong J‐type excitonic coupling.[^43^, ^44^] A distinctive feature of **6Ac‐P** dimers is their considerably broader absorption line widths than monomers due to inhomogeneous broadening caused by various rotational conformers along the acetylene bonds.[^35^, ^38^] The significant CT character in the local excited states of many of these 6,6‐linked dimers could also be a contributing factor to the spectral broadening. However, we believe this to be a negligible component based on previous studies on 6,6‐ linked dimers which show minimal changes in spectral shape with solvent polarity.[^8^, ^13^, ^15^, ^36^, ^41^, ^45^]

In contrast, **2‐P** dimers exhibit moderate redsshifts (around 50 meV) and their absorption linewidths are similar to those of monomers, indicating a limited rotational distribution along the single‐bond axis. Intriguingly, the acetylene bonds in **2Ac‐P** dimers appear to impact the electronic structures of **2‐P** dimers minimally: the absorption peak positions in **2Ac‐P** dimers closely resemble those in **2‐P** dimers. Moreover, the absorption line widths remain unchanged despite expectations of a broad distribution of rotational conformers due to acetylene bonds. This suggests that chromophore rotation does not significantly affect conjugation through the 2,2’ axis, unlike in the 6,6’ case. This surprising effect could potentially arise from the difference in orbital topology at 2 and 6 positions given that a change in the orbital density at these carbon centres would significantly affect the excitonic coupling, such as that observed for the 450–500 nm CT type transition in **2‐P2** and **2Ac‐P2**.[^16^, ^27^] However, the S_0_→S_1_ transition in the pentacene dimers is primarily a pentacene‐localised HOMO→LUMO transition. The presence of significant electron density at both the 6 and 2 carbon centres of the HOMO and LUMO in the pentacene monomers (SI Section 3) means that orbital topology effects should be minor here, and that they cannot alone explain the drastic differences in the S_0_→S_1_ transition.^[29,46][47]^ Alternatively, we find an interplay of long‐range coulombic coupling (J_coul_), influenced by the alignment between the conjugation axis and S_0_→S_1_ transition dipole moments, and short‐range CT type HOMO‐HOMO and LUMU‐LUMO interactions determines the S_0_→S_1_ transition energies[^39^, ^48^, ^49^, ^50^, ^51^] As a result, due to the misalignment of the conjugation axis and S_0_→S_1_ transition dipole, **2Ac‐P** dimers exhibit a broad conformational range similar to **6Ac‐P** dimers yet maintain a narrow energetic distribution reminiscent of **2‐P** dimers. CT mixed higher energy transitions show signatures of broadening particularly in **2Ac‐P2Ph** and **2Ac‐P2BP** which indicates that these transitions in the 2,2’‐linked dimers might be more sensitive to conformational distributions. However, the mixed nature of these higher energy transitions prevents any meaningful quantitative analysis of the absorption linewidths.

The differences in the spectral broadness and absorption peak positions between 2,2’‐ and 6,6’‐linkages can be attributed to varying degrees of exciton coupling, which are influenced by the inter‐pentacene and pentacene‐linker dihedral angles. All dimer classes exhibit interchromophore coupling via both through‐space and through‐bond mechanisms, where linkers control the balance between the two.[^9^, ^21^, ^35^, ^52^, ^53^, ^54^] Among them, the extent of these short‐range interactions can tentatively be inferred from the energy splitting in the frontier molecular orbitals (FMOs), specifically Δ*E*_LUMO_ (*E*_LUMO+1_–*E*_LUMO_) and Δ*E*_HOMO_ (*E*_HOMO_–*E*_HOMO‐1_).[^49^, ^50^, ^55^, ^56^] Without these short‐range interactions, the monomer pentacene FMOs are expected to be degenerate. However, all the dimers exhibit splitting of the FMO levels indicating different extent of HOMO‐HOMO and LUMO‐LUMO orbital overlaps between monomeric units and hence a method to compare the short range inter‐pentacene couplings (Figure S3.2.1–3.2.3). By analyzing these values based on linkage types and interchromophore dihedral angles, we observed that, for ground‐state optimized geometries, the energy splitting is significantly larger in 6,6’‐linkages compared to 2,2’‐linkages when the same number of *para*‐phenyl linkers are present (Figure 3b), which is in line with the degrees of spectral redshift relative to the reference monomers. Additionally, considering all potential rotational conformers, the energy splitting distribution for 6,6’‐linkages is notably broader than that for 2,2’‐linkages (Figure S3.2.4–3.2.9), corroborating the observation of broader absorption line shapes in **6Ac‐P** dimers.^[35]^ The **2Ac‐P** dimers, in general, exhibit behavior similar to the **2‐P** dimers, with only slight variations in the FMO splitting. These calculations suggest that the acetylene bond primarily contributes to (electronically benign) conformational heterogeneity and does not meaningfully perturb the electronic structure established through the 2,2’‐connection.

**Figure 4:**
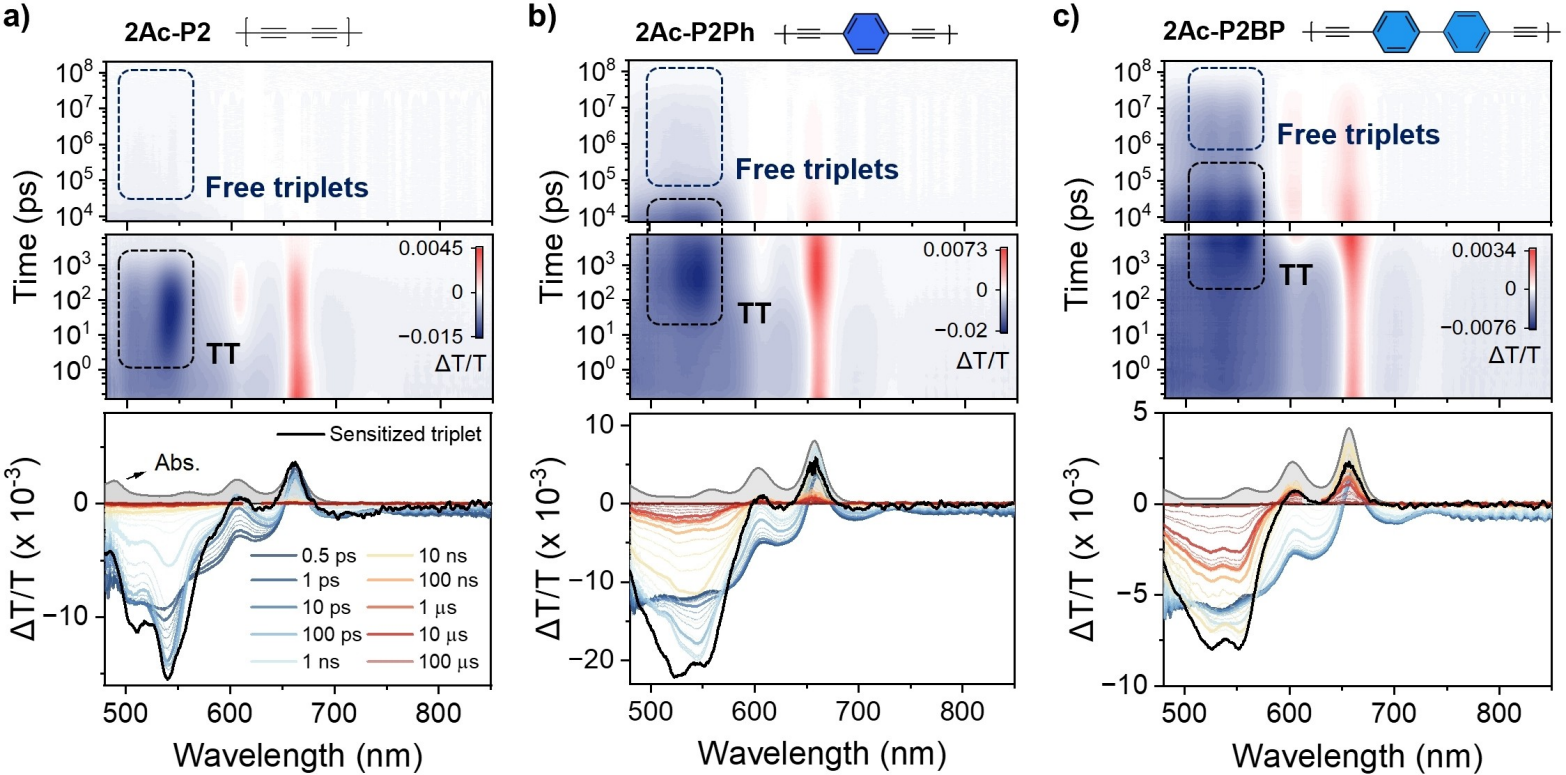
fs‐nsTA results of (a) **2Ac‐P2**, (b) **2Ac‐P2Ph**, and (c) **2Ac‐P2BP** in chlorobenzene (c ~10^−5^ M) after photoexcitation at 0–1 vibronic absorption bands (fsTA – 605 nm, nsTA – 620 nm). Top and bottom panels show contour maps and TA spectra at selected delay times, respectively. Steady‐state absorption (grey filled) and sensitized triplet spectra (black) of each compound are also shown for comparison in the bottom panels.

**iSF Dynamics in 2Ac‐P Dimers**. We employed transient absorption (TA) spectroscopy, utilizing excitation at the 0–1 vibronic band (fsTA at 605 nm and nsTA at 620 nm), to investigate the iSF dynamics of **2Ac‐P** dimers over the fs‐μs timescale (Figure 4). Immediately following photoexcitation, the dimers exhibit a ground‐state bleach (GSB, ΔT/T >0) aligned with the S_0_→S_1_ absorption band, alongside broad photoinduced absorption (PIA, ΔT/T <0) signals in the range of 480–650 nm, corresponding to the S_1_→S_n_ transition in TIPS‐pentacene derivatives and their dimers.[^8^, ^9^, ^10^, ^35^] Additionally, a weak PIA is observed in the NIR spectrum (700–900 nm), with stimulated emission (SE) around 730 nm. As the delay time increases, the SE feature diminishes, while the broad S_1_ PIA feature transforms into vibronically resolved PIA signals of TT, which are similar to the sensitized triplet signals (Figure 4, bottom), across all **2Ac‐P** dimers. The spectral shapes and peak positions of the triplet absorption in the visible spectrum, indicative of a T_1_→T_3_ transition of pentacene derivatives, display notable differences between the three‐dimer series (Figure S4.2.2–4.2.3).[^9^, ^10^] These distinctions are attributed to the alterations in the electronic structures of the PTIPS moiety resulting from substitutions at the 2 and 2’ positions and the wavefunction delocalization extending to the acetylene units, which is evidenced by DFT calculations conducted on reference monomers (Figures S3.4.1).

Multiple isosbestic points, implying direct population transfer between two electronic states, are observed during the spectral evolution from S_1_ to TT (Figures 4 and S4.2.3). This feature enables the direct extraction of time constants for TT formation by analyzing the decay of the S_1_ band (600–650 nm) and the emergence of TT PIA bands (500–550 nm).^[35]^ We observed an increase in TT formation time constants with the addition of *para*‐phenyl linkers, ranging from *n*=0 to *n*=2 (2.7 ps in **2Ac‐P2**; 84 ps in **2Ac‐P2Ph** and 2.0 ns in **2Ac‐P2BP**). This trend mirrors the behavior seen in the **2‐P** and **6Ac‐P** families and can be intuitively understood as a result of reduced inter‐pentacene electronic interactions when introducing *para*‐phenyl linkers. Remarkably, acetylene units in **2Ac‐P** dimers do not lead to any observable dependence of TT formation on the excitation wavelength (Figure S4.2.9). This indicates an absence of the heterogeneous iSF previously noted in 6,6’‐linked dimers that contain acetylene bonds, including the **6Ac‐P** dimers studied in this work.[^35^, ^36^] This outcome contradicts our expectations based on the results from DFT calculations, which predicted a wide distribution of rotational conformers (Figure 2). We propose that this apparent anomaly may be attributed to the electronic structures’ insensitivity to chromophore rotation around the acetylene bond via the 2,2’‐linkage. This observation underscores the insight that conformational heterogeneity does not necessarily equate to diverse dynamics in the excited state.

Following the formation of the TT state, all **2Ac‐P** dimers exhibit multiphasic decay, typically biphasic, a hallmark of triplet dynamics in iSF systems. In particular, these rich decay dynamics confirm that the triplet formation mechanism is iSF even in **2Ac‐P2BP** despite its very slow time constant.[^10^, ^57^, ^58^] The rapid decay components correspond to TT recombination, while the slower one extends into the microsecond regime and represents the decay of free triplets, similar to the lifetime of sensitized triplets (Figure S4.2.8). Notably, in the directly linked **2Ac‐P** dimer the free triplet decay is barely seen. This phenomenon can be attributed to the rapid TT recombination with a time constant τ=860 ps, which impedes the slower spin evolution that typically occurs over tens of nanoseconds. This observation is also linked to the electronic interactions within the dimer, where the stronger interaction prevents the formation of the quintet spin multiplicity in the TT state due to intense intertriplet exchange coupling (*J*).[^59^, ^60^] On the other hand, **2Ac‐P2Ph** and **2Ac‐P2BP** exhibit substantial amounts of free triplets, underscoring the crucial influence of linkers in facilitating the decoherence of spin entanglement within TT by diminishing *J*. Notably, the more gradual TT recombination in **2Ac‐P2BP** (with a time constant τ=48.8 ns) relative to that in **2Ac‐P2Ph** (τ=18.2 ns) is associated with a correspondingly higher prevalence of free triplets. This observation reinforces the previously mentioned concept regarding the impact of linker‐mediated dynamics on free‐triplet generation.

**Figure 5:**
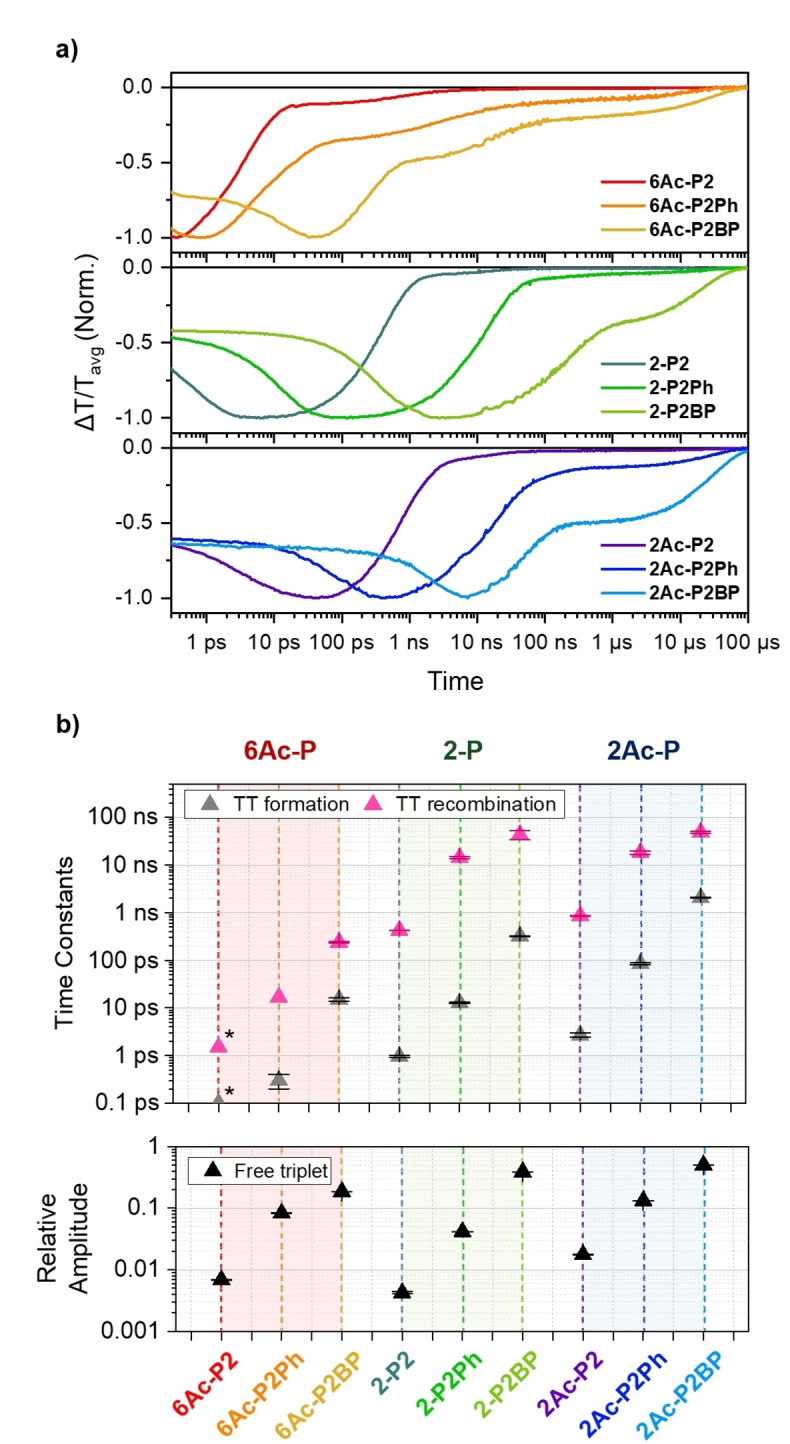
(a) Comparison of normalized kinetics in TT PIA regions among **6Ac‐P** (top), **2‐P** (middle) and **2Ac‐P** (bottom) dimers. (b) Time constants of TT formation and recombination (top) and relative amplitudes of free triplet decay in the kinetics (bottom) shown in panel (a) obtained from multiexponential fits for all dimers. The values with asterisks for the **6Ac‐P2** dimer were taken from the Ref. [29] (See Supporting Information for details). Error bars indicated with horizontal lines.

**Comparison of iSF Dynamics among 2Ac‐P, 2‐P, and 6Ac‐P Dimers**. In addition to our investigation of the iSF dynamics within **2Ac‐P** dimers, we extended our analysis to the **2‐P** and **6Ac‐P** series. This comparison was conducted to thoroughly understand how acetylene linkers and chromophore connectivity influence the overall TT formation and recombination dynamics. The full decay dynamics are complex and proceed over multiple timescales (See Supporting Information Section 4), but we focus our analysis on the first TT recombination process. This step carries the highest weight in the population dynamics and should correspond to decay of the ^1^(TT) state in competition with spin evolution. It is also the recombination process that is most strongly affected by molecular structure. Figures 5a and 5b present a comparative analysis of the kinetics observed within the TT PIA regions and the time constants related to TT formation and recombination for all the dimers. At the first glance, the **6Ac‐P** dimer exhibits faster TT formation and recombination kinetics compared to the other two dimers, and when comparing **2‐P** and **2Ac‐P**, **2‐P** exhibits slightly faster kinetics (Figure 5a). The simplified generalization of the dynamics however overlooks some significant details such as an enhanced primary ^1^TT recombination rate relative to its ^1^TT formation in **2Ac‐P** dimers compared to **2‐P** analogues (SI Figure 4.2.19). This highlights the significant challenges in reconciling the trends in behavior across the full set of 9 dimers. As we illustrate through our discussion below, simple and intuitive design principles like inter‐chromophore distance, linkage position, frontier orbital splitting, or conformational flexibility fail to capture the full range of behavior.[^9^, ^10^, ^15^, ^46^] Instead, the dynamics are governed by a complex interplay of electronic couplings, which set the basic timescales of TT formation and recombination, with structural dynamics that modulate these couplings and provide new pathways for triplet‐pair recombination or spin evolution.^[36]^

In detail, considering the number of *para*‐phenyl units in the linkers, it is observed that when the number remains constant, **6Ac‐P** dimers show faster TT formation and recombination compared to **2‐P** and **2Ac‐P** dimers (Figure 5b). This effect is due to the connectivity at the 6,6’‐position, which facilitates a significantly stronger inter‐pentacene coupling than the other dimers. This observation aligns with the trends in energy splitting within the FMOs (Figure 3b) and the positions of the 0–0 peaks in the ground‐state absorption (Figure 3a). Comparing **2‐P** to **2Ac‐P** dimers, the latter exhibits slower TT formation and recombination time constants, even considering the similarity of peak positions in ground‐state absorption. This shows that the energy splitting within the FMOs presented above does not provide the full picture, as it depends on the number of *para*‐phenyl units, and whether **2‐P** or **2Ac‐P** exhibits larger splitting. We can rationalize the observed behavior by considering the nuances of interpentacene distance that govern the electronic coupling. Two different geometrical conventions can be applied to establish this distance: 1) the center‐to‐center distance between pentacene chromophores, and 2) the edge‐to‐edge distance between the effective chromophore units. When focusing on the edge‐to‐edge criterion, we should consider the electronic wavefunctions in **2Ac‐P** dimers extended to the acetylene units (Figure S3.4.1).

Consequently, for chromophores with an equivalent number of *para*‐phenyl linkers, the distances between two chromophores remain consistent in both **2Ac‐P** and **2‐P** dimers. Accordingly, based on a simple edge‐to‐edge distance we should expect similar dynamics in the analogues of **2‐P** and **2Ac‐P** dimers in contrast to what is observed observed (SI Figure 3.4.2–3.4.5). This indicates that even subtle changes in the electronic wavefunction with different bridges as in **2Ac‐P** compared to **2‐P** substantially alters the coupling between the chromophores. Even within the **2Ac‐P** series the orbital density extends onto the acetylene bridge to varying degrees in the *n*=0,1,2 dimers making the accurate estimation of edge‐to‐edge distance even more difficult. Thus, attention must be directed towards the center‐to‐center distances between pentacenes. This parameter also alone fails to clarify the distinctions observed between the two dimer families such as an enhanced primary recombination rate in **2Ac‐P** dimers compared **2‐P** analogues with similar center‐to‐center distances (See Table S3.1.1). On the other hand, applying this metric to the **6Ac‐P** dimers would suggest they are similar to **2Ac‐P** dimers, which is not the case. Instead, given the more substantial redshifts in the **6Ac‐P** absorption, the faster TT formation and recombination dynamics in **6Ac‐P** compared to **2Ac‐P** emphasizes the critical role of the spatial arrangement and suggests a deeper investigation into how the interplay between these distances and dimer linkage position influence electronic coupling strengths and the resulting kinetics. Additionally, an analysis based on inter‐chromophore distance alone needs to acknowledge the role of structural fluctuations generated by the acetylene linkages. Such structural fluctuations allow variations in the electronic coupling with strongly coupled planar subpopulations providing additional recombination pathways thus facilitating ^1^TT recombination in these dimers as compared to the relatively rigid **2‐P** dimers.

Our subsequent analysis examines the proportion of long‐lived free triplets across all dimers. In contrast to the phenomena observed in thin films or single crystals, generating free triplets through iSF in molecular dimers is generally less efficient. This inefficiency arises because the spatial separation required for the TT state to form and for electronic and spin states to decorrelate is inherently challenging to achieve. Instead, within molecular dimers, the structural dynamics of the chromophores or their linkers predominantly dictate the spin evolution from singlet to quintet states within the TT manifolds by fluctuating *J*,[^35^, ^60^, ^61^] ultimately determining the overall yield of free triplets. In the case of strongly coupled dimers without *para*‐phenyl linkers, the expectation is that decorrelation processes would be challenging to initiate, primarily due to inherently high *J* values. However, the presence of a modest amount of long‐lived triplet signals hints at a minor subset of geometries capable of facilitating the formation of free triplets even in these strongly coupled dimers, although the exact geometry is unclear at the current stage. For dimers incorporating a single *para*‐phenyl unit, this configuration not only diminishes the *J* value by decreasing the electronic coupling between pentacene units but also introduces additional fluctuating exchange interactions through the structural dynamics, i.e. torsion of the phenyl moiety.[^60^, ^61^] This mechanism significantly enhances the triplet generation compared to directly connected dimers. The presence of biphenyl units amplifies this effect even further, leading to a more pronounced increase in the triplet population relative to dimers with fewer phenyl units.

Within the three dimer series, we identified two distinct trends in relative triplet yield from the viewpoint of the number of *para*‐phenyl units: 1) for directly linked and single *para*‐phenyl substituted dimers, the progression is **2‐P**<**6Ac‐P**<**2Ac‐P**; and 2) for dimers substituted with biphenyl, the sequence is **6Ac‐P**<**2‐P**<**2Ac‐P**. Typically, a rapid TT recombination is associated with lower yields of free triplets due to the large *J* values, as previously discussed. However, the first trend underscores the role of geometrical fluctuations, prompted by acetylene units, in facilitating the generation of free triplets despite the large *J* values inherent to an energy‐minimized geometry. In contrast, the presence of biphenyl places the dimers within a regime of weaker coupling, making the intrinsic *J* value a more critical factor in the generation of free triplets than fluctuations in *J* caused by structural dynamics. Across all observed trends, **2Ac‐P** dimers consistently exhibit stronger triplet signals than the others, suggesting that acetylene units significantly enhance the generation of a larger number of triplets, even though the TT formation rates of **2Ac‐P** dimers are slower than those of **2‐P** dimers, due to the greater inter‐pentacene distances. When comparing **2Ac‐P** with **6Ac‐P**, **2Ac‐P** consistently exhibits higher yields than **6Ac‐P**. This indicates that the stronger *J* associated with 6,6’‐connectivity, despite both dimers having an equal number of phenyl units, is detrimental to the formation of long‐lived free triplets.

We can further explore the trends in relative triplet yields from two additional perspectives: 1) center‐to‐center distances and 2) edge‐to‐edge distances between pentacene units. Examining the first perspective reveals a trend of **2Ac‐P**<**2‐P**<**6Ac‐P**. For example, **6Ac‐P2BP**, **2‐P2Ph**, and **2Ac‐P2** exhibit nearly identical center‐to‐center distances (approximately 18 Å), with **6Ac‐P2BP** being the largest, followed by **2‐P2Ph**, and then **2Ac‐P2**. This pattern can be attributed to the number of phenyl linkers, which influences the triplet yields irrespective of the linkage between chromophores. Specifically, the torsional flexibility of phenyl units plays a crucial role in enhancing the free triplet yields from the TT state. This principle is similarly relevant in analyzing trends from the second perspective. At comparable edge‐to‐edge distances, the trend is observed as **6Ac‐P**<**2Ac‐P**<**2‐P**. For instance, considering the similar edge‐to‐edge distance of approximately 6 Å, **2‐P2Ph** reveals higher relative triplet yields compared to **6Ac‐P2** and **2Ac‐P2**, attributed to the presence of a para‐phenyl unit. In addition, from this angle, **6Ac‐P** dimers present lower yields than **2Ac‐P** dimers, which can be linked to differences in inter‐pentacene connectivity (2,2’ versus 6,6’), as discussed above. Overall, these comparative analyses among the three types of dimers with viewpoints of different geometrical factors offer profound insights into the interplay between intrinsic electronic coupling among chromophores and their structural adaptability in influencing TT dynamics and the generation of free triplets.

## Conclusion

In this study, we have meticulously explored the influence of the acetylene bridge on iSF dynamics by examining a set of 2,2′‐linked pentacene dimers that incorporate an acetylene bridge, **2Ac‐P**, with the comparison of previously reported 2,2’‐ and 6,6’‐linked pentacene dimers **2‐P** and **6Ac‐P**. Although the electronic wavefunctions of a pentacene chromophore are delocalized to acetylene units in **2Ac‐P**, it does not significantly alter the electronic structure of their motif dimers, **2‐P**, but induces conformational heterogeneity. Briefly, the iSF dynamics of **2Ac‐P** is the slowest among the dimer series we compared, which is due to the combination of elongated inter‐pentacene distances (compared with **2‐P** dimers) and weaker electronic coupling (compared with **6Ac‐P** dimers). Despite conformational heterogeneity through the acetylene bonds, the heterogeneous SF effect known in **6Ac‐P** dimers was not observed in the 2,2’ linkage, due to the insensitivity of variation in electronic structures along torsional coordinates in **2Ac‐P** dimers. However, the structural flexibility afforded by easy rotation around the acetylene bridges does have an important impact on the subsequent evolution of the triplet pair. Such flexibility enables the dimers to adopt planar geometries. On one hand, these introduce parasitic ^1^(TT) recombination pathways, resulting in faster ^1^(TT) recombination relative to its formation in the acetylene‐bridged structures. On the other hand, this flexibility promotes the generation of free triplets through fluctuating exchange couplings in dimers with naturally large exchange (*J*) values. On balance, we consider the latter effect to outweigh the accelerated ^1^(TT) decay, making acetylene bridges a boon for most iSF applications. The significant contribution from these bridges on the electronic structure furthermore challenges the traditional separation of chromophore and bridge units in the literature, especially in scenarios where the initial excitation extends significantly onto the bridge units. In cases like these, even the determination of seemingly simple parameters like the distance between SF‐active units becomes ambiguous. While the structures of these and similar iSF dimers are synthetically modular, that property does not necessarily extend to their electronic states. As we seek to extract deeper design principles from comparison of diverse bridging motifs, our observations underscore the necessity for a deeper understanding of the spatial arrangement of excited states.

## Associated Content

All experimental procedures are reported in Supporting Information Section 1. Synthesis and characterizations of pentacene dimers are reported in the Supporting Information Section 2 and 5.

## Notes

The authors declare no competing financial interests.

## Author Contributions

S.P. and A.J.M. conceived the project; K.M. synthesized the samples under the supervision of S.P.; K.M. performed structural characterisation, including steady‐state optical measurements; S.M. performed fs‐TA experiments.; J.P. performed ns‐TA experiments.; K.M. and W.K. performed DFT calculations.; K.M., W.K. and S.M. analysed the TA results with input from A.J.M.; All authors discussed and commented on the experiments and results. K.M., W.K., S.M., S.P. and A.J.M. wrote the paper with input from all authors. All the co‐authors read and agreed with the final version of the manuscript.

## Conflict of Interests

The authors declare no conflict of interest.

1

## Supporting information

As a service to our authors and readers, this journal provides supporting information supplied by the authors. Such materials are peer reviewed and may be re‐organized for online delivery, but are not copy‐edited or typeset. Technical support issues arising from supporting information (other than missing files) should be addressed to the authors.

Supporting Information

## Data Availability

The data that support the findings of this study are available in the supplementary material of this article.
