# Supplementary material for: The Acetylene Bridge in Intramolecular Singlet Fission: A Boon or A Nuisance?
Source: Angew Chem Int Ed Engl. 2024 Nov 18;63(52):e202408615. doi: 10.1002/anie.202408615 (PMC11656137; doi:10.1002/anie.202408615)
Supplement: Supplementary file 1 — Supporting Information [file ANIE-63-e202408615-s001.pdf]

Angewandte Chemie
Eine Zeitschrift der Gesellschaft Deutscher Chemiker

Supporting Information

The Acetylene Bridge in Intramolecular Singlet Fission: A Boon or A Nuisance?

K. Majumder, S. Mukherjee, J. Park, W. Kim, A. J. Musser*, S. Patil**

Supporting Information:

The Acetylene Bridge in Singlet Fission: A Boon or A Nuisance?

Kanad Majumder[1], Soham Mukherjee[2], Jungjin Park[3], Woojae Kim[3,*], Andrew J. Musser[2,*], Satish Patil[1,*]

[1]Solid State and Structural Chemistry Unit, Indian Institute of Science, Bangalore 560012, India

[2]Department of Chemistry and Chemical Biology, Cornell University, Ithaca, New York 14853, USA

[3]Department of Chemistry, Yonsei University, Seoul 03722, Republic of Korea

Correspondence to: woojae@yonsei.ac.kr, ajm557@cornell.edu, spatil@iisc.ac.in

Table of Contents

1. **General Materials and Methods**..........4

2. **Synthesis and Spectroscopic Characterization of Compounds**..........6

    2.1. General Synthetic Procedure for Synthesis of 2,2' Pentacene Dimers..........7

        2.1.1. Synthesis of 2,2' Pentacene Dimers (2Ac-P2Ph and 2Ac-P2BP)..........7

        2.1.2. Synthesis of 2,2' Pentacene Dimer (2Ac-P2)..........8

        2.1.3. Synthesis of 2,2' Pentacene Dimers (2-P2Ph and 2-P2BP).....9

        2.1.4. Synthesis of 2,2' Pentacene Dimer (2-P2)..........10

    2.2. Synthesis of Pentacene Precursor for 2,2'-linked Pentacene Dimers..........11

    2.3. Synthesis of Diethynyl derivative of Oligo-(phenylene) Linkers..........15

    2.4. General Synthetic Procedure for Synthesis of 6,6' Pentacene Dimers..........16

3. **Computational Methods**..........23

    3.1. Structural Parameters..........24

    3.2. Energy level splitting..........27

    3.3. Ground State Optimised Geometries..........36

    3.4. Triplet Spin Density..........38

4. **Supporting Figures**..........43

    4.1. Steady State Measurements..........43

4.2. Transient Absorption Measurements..45

5. ^{1}H, ^{13}C NMR Spectra and MALDI of Compounds...63

6. References..99

1. General Materials and Methods:

Materials.

All solvents were dried by standard methods. Chemicals were purchased from Aldrich, Acros Organics, S.D. fine chemicals, B.L.D. Pharm, T.C.I. India and Spectrochem and used without further purification. Compound 3a-h were synthesized via reported procedures as described later. THF was distilled from sodium/benzophenone ketyl. Anhydrous Na_2SO_4 was used as the drying agent after aqueous work-up. Evaporation and concentration in vacuo was done at water aspirator pressure. All reactions were performed in standard, dry glassware under an inert atmosphere of nitrogen or argon. Column chromatography: silica gel-60 (230–400 mesh). Thin Layer Chromatography (TLC): pre-coated plastic sheets covered with 0.20 mm silica gel with fluorescent indicator UV 254 nm; visualization by UV light. Detailed synthesis procedures are provided in Supplementary Information Section 2.

Sample Characterisation and Steady-state Optical Characterization.

The 1H spectra were taken in ECX500 - Jeol 500 MHz High Resolution Multinuclear FT-NMR Spectrometer.at 500 MHz frequency. The chemical shifts were reported as δ values (ppm) relative to TMS. Also, 13C were recorded on the same instrument at 125 MHz frequency. All sample were dissolved in CDCl3 / d6-DMSO solvent. MALDI-MS were recorded on a Bruker daltonics Autoflex Speed system using α-Cyano-4- hydroxy-cinnamic acid (CCA) as a matrix.

The optical absorption spectra of the molecules in solution were recorded with PerkinElmer (Lambda-35) spectrometer at room temperature. Spectroscopic grade solvents were used for performing all the studies. High concentration stock solution was prepared in chlorobenzene and finally diluted to 10 µM (10×10^{-6} M) concentration to perform all the steady state experiments.

Density Functional Theory Calculations were done with Gaussian 16 Program.[1] The geometry optimization and potential energy curve scan calculations were performed using CAM-B3LYP functional[2] with 6-31g(d) basis set.[3]

Transient Absorption Spectroscopy.

Femtosecond transient absorption measurements were performed with an automated transient absorption spectrometer (HELIOS, Ultrafast Systems), driven by the Yb:KGW amplifier (PHAROS-SP, Light Conversion) operating at 8 kHz. The OPA and SH module (ORPHEUS and LYRA-SH, Light Conversion) generate a180-fs narrowband pump pulse. A portion of the fundamental was separated to generate a white light continuum probe pulse ranging from 450 nm to 1600 nm using 1 cm sapphire (VIS, 450-915 nm) and YAG (NIR, 1120-1630 nm) crystal. For UV probe ranging from 350 nm to 510 nm, the frequency doubled 515 nm was used. The beam diameters (1/e^2 height) for pump and probe pulses at the sample position were 650 and 140 μm, respectively. TA spectra were collected with magic angle condition between pump and probe and in a shot-to-shot fashion. Pump-probe time delay was set by a mechanical delay stage from -3 to 7600 ps. A 2 mm path length cuvette (Hellma, HL110-2-40) was used. A magnetic stirrer (Ultrafast Systems) was used to prevent photodegradation of the sample. The nanosecond TA experiments were conducted by an automated TA spectrometer (EOS, Ultrafast Systems) with a combination of two independent lasers: a Yb:KGW amplifier (Pharos-SP-1.5 mJ, Light Conversion) with a 1 kHz repetition rate and a supercontinuum laser (LEUKOS) with a 2 kHz repetition rate. Pump pulses were generated through a commercial collinear optical parametric amplifier and its second harmonic module (ORPHEUS and LYRA, Light Conversion). Time delays were electronically controlled. A quartz cell with a 2 mm path length (21/Q/2, Starna) was employed with the sample concentration of c = ~ 10^{-5} M. The samples were prepared inside a glovebox (0.0 ppm O_2 level) with fresh anhydrous solvents and sealed with Teflon tape and parafilm. After every experiment, the steady-state absorption was carefully checked, and we confirmed that there was no degradation of the sample.

2. Synthetic and Spectroscopic Characterisation of Compounds

a) Synthesis of 2,2'-linked Pentacene Dimers **(2Ac-P):**

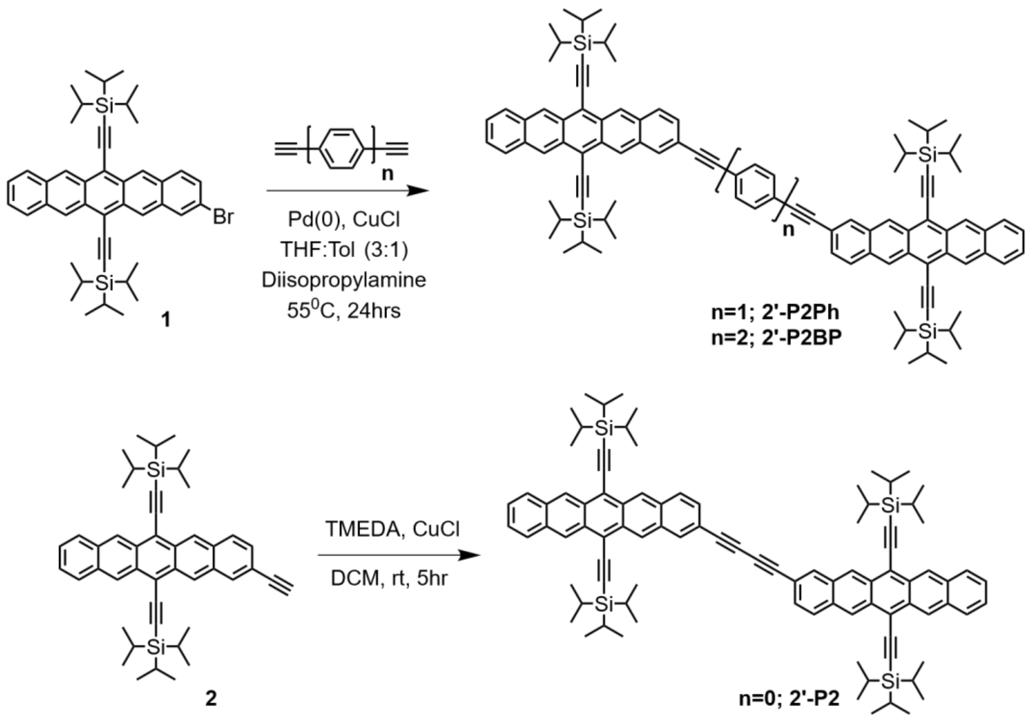

b) Synthesis of 2,2'-linked Pentacene Dimers **(2-P):**

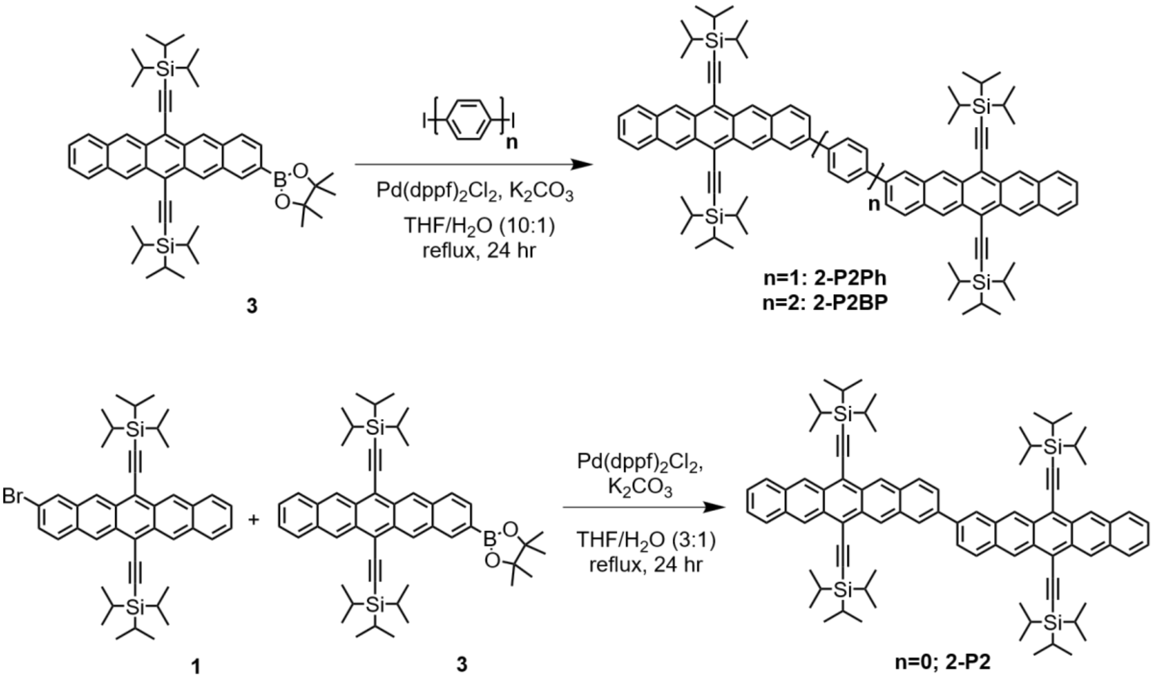

Figure S2.1. Synthesis of 2,2'-linked pentacene dimers

2.1. General Synthetic Procedure for Synthesis of 2,2′ Pentacene Dimers

The targeted molecules **2Ac-P** were synthesized via carbon-carbon Sonogashira and carbon-carbon homocoupling. The targeted molecules **2-P** were synthesized according to the known procedure as described in the literature.[4] The synthetic route and the molecular structures are illustrated in Scheme S2.1. Intermediates and final compounds were purified by silica gel chromatography; structures and purity were confirmed by ^{1}H, ^{13}C and MALDI-TOF.

2.1.1. Synthesis of 2,2′ Pentacene Dimers (2Ac-P2Ph and 2Ac-P2BP):

To a solution of **1** (0.300 g, 0.42 mmol, 2 equiv) and diethynyl derivative of the oligophenylene linker, **5b** (0.21 mmol, 1 equiv) in dry THF and toluene mixture (3:1, 15mL) and diisopropylamine (5.0 mL, 3.6 g, 36 mmol) which had been deoxygenated for 10 min with argon was added $Pd(PPh_3)_4$ (0.025 g, 0.0216 mmol) and CuCl (0.008 g, 0.08 mmol). The reaction mixture was further deoxygenated with argon for an additional 2 min. The reaction mixture was stirred for 24 h at 55 °C, cooled to rt, and poured into satd. aq. NH_4Cl (100 mL). H_2O (150 mL) was added, and the mixture was extracted with CH_2Cl_2 (80 mL, 50 mL). The organic phase was washed with 5% NH_4Cl (200 mL), dried (Na_2SO_4), filtered, and the solvent removed in vacuo. Column chromatography (silica gel, hexanes/chloroform) afforded the product (yield 55-65%) as a blue-green solid.

Spectroscopic Characterisation of 2Ac-P2Ph:

Yield: 0.208 g (71%). The product has low solubility, thus ^{1}H and ^{13}C NMR spectroscopy have poorly defined signals. ^{1}H NMR (500 MHz, $CDCl_3$): δ 9.30-9.28 (br, m, 4H), 9.26-9.24 (br, 4H), 8.16 (s, 2H), 7.98-7.95 (m, 4H), 7.95-7.92 (d, 2H), 7.64 (s, 4H), 7.46-7.43 (d, 2H), 7.43-7.40 (m, 4H), 1.41-1.35 (m, 84H). ^{13}C NMR (125 MHz, $CDCl_3$): δ 132.62, 132.50, 131.91, 131.84, 131.74, 131.64, 131.52, 131.17, 131.04, 130.98, 130.86, 130.82, 129.04, 128.88, 128.77, 128.72, 128.65, 128.04, 126.79, 126.72, 126.66, 126.55, 126.49, 126.48, 126.27, 123.29, 120.46, 118.73, 118.65, 107.72, 107.47, 104.54, 92.43, 91.10, 19.10, 11.75. MALDI-MS m/z calcd. for $C_{98}H_{110}Si_4$ (M^+) 1398.768, found 1398.222.

Spectroscopic Characterisation of 2Ac-P2BP:

Yield: 0.238g (77%). The product has low solubility, thus ^{1}H and ^{13}C NMR spectroscopy have poorly defined signals. ^{1}H NMR (500 MHz, $CDCl_3$): δ 9.31-9.28 (br, m, 4H), 9.27-9.23 (br, 4H), 8.16 (s, 2H), 7.98-7.95 (m, 4H), 7.95-7.92 (d, 2H), 7.72-7.66 (m, 6H), 7.63-7.60 (m,2H), 7.48-7.45 (d, 2H), 7.43-7.40 (m, 4H), 1.41-1.35 (m, 84H). ^{13}C NMR (125 MHz, $CDCl_3$): δ 140.26, 133.16, 132.48, 132.37, 131.68, 131.17, 131.01, 130.98, 130.87, 130.81, 128.98, 128.77, 128.68, 128.16, 127.05, 126.90, 126.63, 126.26, 122.67, 122.61, 120.65, 120.59, 118.70, 118.63, 107.67, 107.44, 104.57, 91.42, 91.22, 19.13, 11.76. MALDI-MS m/z calcd. for $C_{104}H_{114}Si_4$ (M^+) 1474.799, found 1474.310.

2.1.2. Synthesis of 2,2′ Pentacene Dimer (2Ac-P2):

To a suspension of CuCl (0.040 g, 0.40 mmol) in TMEDA (1.1 mL, 0.85 g, 7.3 mmol) was added dry CH_2Cl_2 (10 mL). This suspension was oxygenated by bubbling O_2 for approx. 2 min. To this mixture was added a solution of **2** (0.250 g, 0.377 mmol) in dry CH_2Cl_2 (3 mL). The reaction mixture was allowed to stir 4 h at rt open to the atmosphere and then poured into satd. aq. NH_4Cl (200 mL). The mixture was extracted with CH_2Cl_2 (2 × 50 mL). The combined organic phase was washed with H_2O (200 mL, 150 mL), satd. aq. NaCl (150 mL), dried (Na_2SO_4), and the solvent removed in vacuo. Column chromatography (silica gel, 4:1 CH2Cl2/hexanes) afforded the product (0.119 g, 55%) as a purplish-blue solid.

Spectroscopic Characterisation of 2Ac-P2:

Yield: 0.140g (56%). The product has low solubility, thus ^{1}H and ^{13}C NMR spectroscopy have poorly defined signals. ^{1}H NMR (500 MHz, $CDCl_3$): δ 9.31-9.28 (br, m, 4H), 9.26-9.24 (br, 4H), 8.24 (s, 2H), 7.98-7.95 (m, 4H), 7.94-7.91 (d, 2H), 7.43-7.39 (m, 6H), 1.38-1.35 (m, 84H). ^{13}C NMR (125 MHz, $CDCl_3$): δ 134.72, 132.58, 132.53, 131.34, 131.16, 131.14, 130.97, 130.90, 130.85, 129.21, 128.79, 128.77, 127.58, 127.08, 126.67, 126.54, 126.49, 126.37, 126.33, 119.17, 118.96, 118.69, 107.95, 107.61, 104.44, 83.74, 75.97, 19.12, 19.09, 11.75, 11.74. MALDI-MS m/z calcd. for $C_{92}H_{105}Si_4$ ($[M-H]^+$) 1321.729, found 1321.648.

2.1.3. Synthesis of 2,2′ Pentacene Dimers (2-P2Ph and 2-P2BP):

Compound 1 was synthesized according to procedure reported in the literature.[4]

To a dry round bottomed flask was added **3** (0.400 g, 0.52 mmol, 2.2 equiv), diiodo derivative of the oligophenylene linker, (0.24 mmol, 1 equiv), K_2CO_3 (0.52 mg, 17 equiv) and Pd(dppf)$_2$Cl$_2$.DCM (0.1 equiv). Sequential vacuum and argon were used to degas the mixture followed by the addition of degassed THF and H_2O (10:1 ratio, 150 mL). The mixture was heated to reflux and maintained for 24 h in the dark. After the reaction, the mixture was cooled, and poured into satd. aq. NH_4Cl (150 mL). H_2O (100 mL) was added, and the mixture was extracted with CH_2Cl_2 (100 mL, 50 mL). The organic phase was washed with 5% NH_4Cl (200 mL), dried (Na_2SO_4), filtered and the solvent removed in vacuo. Column chromatography (silica gel, hexanes/chloroform) afforded the product (yield 55-65%) as a blue-green solid.

Spectroscopic Characterisation of 2-P2Ph:

Yield: 0.211 g (65%). The product has low solubility, thus ^{1}H and ^{13}C NMR spectroscopy have poorly defined signals. ^{1}H NMR (500 MHz, $CDCl_3$): δ 9.37 (s, 2H), 9.34-9.29 (m, 6H), 8.23 (s, 2H), 8.10-8.07 (m, 2H), 7.99-7.95 (m, 8H), 7.82–7.78 (m, 2H), 7.43-7.39 (m, 4H), 1.41-1.35 (m, 84H). ^{13}C NMR (125 MHz, $CDCl_3$): δ 137.80, 132.53, 132.44, 132.40, 131.60, 131.06, 130.89, 130.86, 130.73, 129.60, 128.78, 127.91, 127.83, 127.80, 127.79, 127.75, 127.70, 126.75, 126.51, 126.44, 126.31, 126.30, 126.21, 26.16, 126.07, 125.94, 125.91, 118.58, 118.39, 107.30, 104.78, 104.73, 19.12, 19.11, 11.78. MALDI-MS m/z calcd. for $C_{94}H_{110}Si_4$ (M^+) 1350.768, found 1350.352.

Spectroscopic Characterisation of 2-P2BP:

Yield: 0.247 g (72%). The product has low solubility, thus ^{1}H NMR spectroscopy have poorly defined signals. ^{13}C NMR could not be obtained due to poor solubility. ^{1}H NMR (500 MHz, $CDCl_3$): δ 9.36 (s, 2H), 9.33-9.28 (m, 6H), 8.21 (s, 2H), 8.10-8.05 (d, 2H), 7.98-7.91 (m, 8H), 7.89–7.86 (m, 4H), 7.79-

7.76 (d, 2H), 7.43-7.39 (m, 4H), 1.41-1.35 (m, 84H). MALDI-MS m/z calcd. for $C_{100}H_{114}Si_4$ (M^+) 1426.799, found 1426.377.

2.1.4. Synthesis of 2,2′ Pentacene Dimer (2-P2):

To a dry round bottomed flask was added **1** (0.15 g, 0.2 mmol), **3** (0.16 g, 0.2 mmol), $Pd(dppf)_2Cl_2$ ·DCM (0.015 g, 0.1 equiv), and K_2CO_3 (500 mg, 3.6 mmol). Sequential vacuum and argon were used to degas the mixture followed by the addition of degassed H_2O (2 mL) and THF (6 mL). The resulting solution was heated to 70 °C and maintained for 24 h in dark. After the reaction, the solution was poured into satd. aq. NH_4Cl (150 mL). H_2O (100 mL) was added, and the mixture was extracted with CH_2Cl_2 (100 mL, 50 mL). The organic phase was washed with 5% NH_4Cl (200 mL), dried (Na_2SO_4), filtered and the solvent removed in vacuo. Column chromatography (silica gel, hexanes/DCM) afforded the product (yield 77%) as a blue-green solid.

Spectroscopic Characterisation of 2-P2:

Yield: 0.173 g (68%). The product has low solubility, thus ^1H and ^{13}C NMR spectroscopy have poorly defined signals. ^1H NMR (500 MHz, $CDCl_3$): δ 9.40 (s, 2H), 9.35-9.28 (m, 6H), 8.33 (s, 2H), 8.15-8.12 (d, 2H), 7.99-7.95 (m, 4H), 7.93–7.90 (d, 2H), 7.43-7.39 (m, 4H), 1.41-1.35 (m, 84H). ^{13}C NMR (125 MHz, $CDCl_3$): δ 137.79, 132.54, 132.52, 132.45, 132.43, 131.66, 131.13, 130.97, 130.86, 130.76, 129.73, 128.78, 126.92, 126.87, 126.45, 126.31, 126.26, 126.17, 126.07, 125.85, 118.61, 118.45, 107.46, 107.31, 104.80, 104.74, 19.11, 11.79. MALDI-MS m/z calcd. for $C_{88}H_{106}Si_4$ (M^+) 1274.737, found 1274.276.

2.2. Synthesis of Pentacene Precursor for 2,2′-linked Pentacene Dimers:

Compound **1,2,3** was synthesized according to procedure reported in the literature.[4]

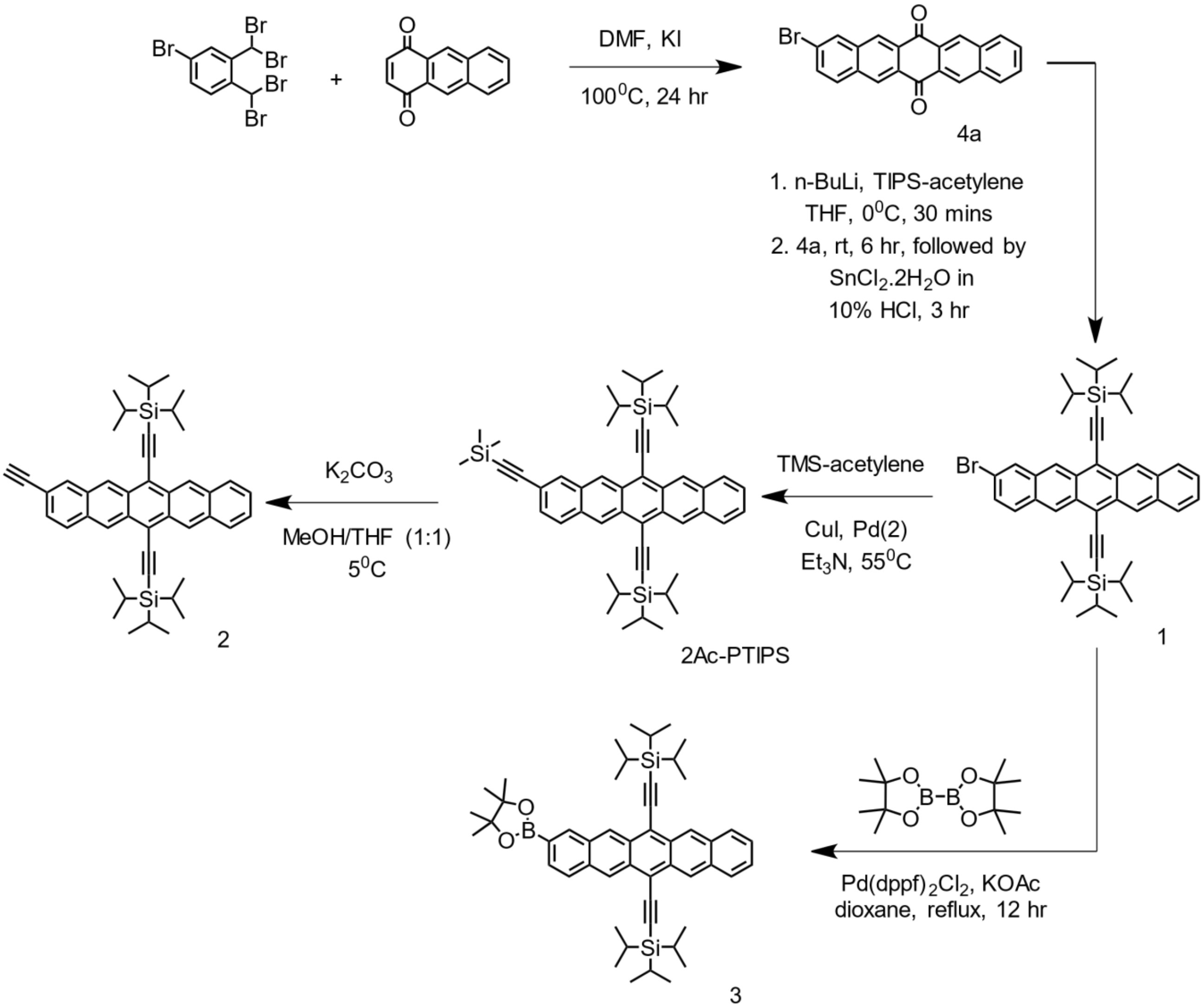

Figure S2.2. Synthesis of Pentacene precursor for 2,2’ linked pentacene dimers.

Anthracene-1,4-dione:

Following a literature procedure[5], quinizarine (15.0 g, 62.5 mmol) was dissolved in 300 mL of methanol, the mixture was then cooled to 0 °C and $NaBH_4$ (9.7 g, 25 mmol) was added portion-wise. The mixture was stirred for 2 h, then 110 mL of a 6 N aqueous solution of HCl were slowly added. The precipitated was filtered and washed with water and cold acetone. 11.6 g (yield 89%) of a red-

orange solid were obtained. The spectral data were in agreement with that previously reported in the literature.

2-bromopentacene-6,13(5aH,13aH)-dione (4a):

Following literature procedures, 4-Bromo-1,2-bis(dibromomethyl)benzene (9.0 g, 18.0 mmol), anthracene-1,4-dione (3.6 g, 17.0 mmol) and NaI (12.6 g, 84 mmol) were dissolved in 70 mL of dimethylformamide. The mixture was stirred at 110 °C for 24 h. Then the reaction was cooled to 0 °C and filtered, the collected solid was washed thoroughly with H_2O, methanol, acetone and diethylether. 4.34 g (yield 66%) of a gold-like insoluble solid were recovered. The spectrometric mass data are in agreement with that previously reported in the literature.

((2-bromopentacene-6,13-diyl)bis(ethyne-2,1-diyl))bis(triisopropylsilane) (1)

Following literature procedures, to a solution of (triisopropylsilyl)acetylene (3.5 equiv.) in dry and degassed THF (25 mL) in 200 mL Schlenk flask at - 78 °C added n-butyl lithium (3.4 equiv., 2.5 M in hexanes). This solution was allowed to stir at -78 °C for 1 h followed by the addition of 3 (4.0 g, 1.0 equiv.) under positive argon flow. The solution was allowed to warm to rt and stirred overnight (16 h) or until solid pentacenequinone was no longer observed. To this clear, deep yellow solution was added of a saturated solution of tin (II) chloride dihydrate in aqueous solution (50 mL) during which the solution turned deep blue. The resulting mixture was stirred at rt for 1 h under dark and filtered over a pad of silica. The solid was washed with DCM and the combined organic layer was washed with water (2 x 200 mL), dried over anhyd. Na_2SO_4, filtered and the solvent was removed under reduced pressure to get the crude product. The crude was purified by silica chromatography using hexanes as an eluent to obtain bromo pentacene derivative **1** as a deep blue solid in 65% yield. 1H NMR (500 MHz, $CDCl_3$, δ ppm): 9.34 (s, 1H), 9.32 (s, 1H), 9.29 (s, 1H), 9.22 (s, 1H), 8.16 (s, 1H), 8.01-7.99 (m, 2H), 7.87-7.86 (m, 1H), 7.48-7.44 (m, 3H) and 1.44-1.38 (m, 42H).

((2-ethynylpentacene-6,13-diyl)bis(ethyne-2,1-diyl))bis(triisopropylsilane)(2):

To a solution of trimethylsilylacetylene (3.0 mL, 2.108 g, 21.46 mmol) and pentacene derivative, **1** (4.0 g, 5.57 mmol), in dry THF and toluene mixture (3:1, 15mL) and diisopropylamine (5.0 mL, 3.6 g, 36 mmol) which had been deoxygenated for 10 min with argon was added $Pd(PPh_3)_4$ (0.030 g, 0.026 mmol) and CuCl (0.010 g, 0.10 mmol). The reaction mixture was further deoxygenated with argon for an additional 2 min. The reaction mixture was stirred for 24 h at 55 °C, cooled to rt, and poured into satd. aq. NH_4Cl (100 mL). H2O (150 mL) was added, and the mixture was extracted with CH_2Cl_2 (80 mL, 50 mL). The organic phase was washed with 5% NH_4Cl (200 mL), dried (Na2SO4), filtered, and the solvent removed in vacuo. Column chromatography (silica gel, hexanes/chloroform) afforded **2Ac-PTIPS** (yield 65%) as a dark green solid.

To a solution of **2Ac-PTIPS** (2 g, 2.72 mmol) in THF (100 mL) and MeOH (100 mL) cooled to 0 °C was added K_2CO_3 (1.21 g, 8.75 mmol). The reaction mixture was maintained between 0 °C and 5 °C and stirred for 5.5 h and then poured into satd. aq. NH_4Cl (200 mL). H2O (50 mL) was added, and the mixture was extracted with hexanes (80 mL, 2 × 50 mL). The combined organic phase was washed with satd. aq. NH_4Cl (200 mL), satd. aq. $NaHCO_3$ (200 mL), satd. aq. NaCl (150 mL), dried (Na_2SO_4), and the solvent removed in vacuo. Column chromatography (silica gel, hexanes/chloroform) afforded **6** (yield 75%) as a dark green solid. 1H NMR (500 MHz, CDCl3): δ 9.28 (s, 2H), 9.24 (s, 2H), 8.14 (s, 1H), 7.97–7.94 (m, 2H), 7.90–7.88 (d, 1H), 7.42-7.38 (m, 2H), 7.36 (d, 1H), 3.27 (s, 1H), 1.36–1.33 (m, 42H).

((2-(4,4,5,5-tetramethyl-1,3,2-dioxaborolan-2-yl)pentacene-6,13-diyl)bis(ethyne-2,1-diyl))bis(triisopropylsilane) (3):

To a dry round bottomed flask was added **1** (4.0 g, 5.57 mmol), $Pd(dppf)_2Cl_2$ ·DCM (203 mg, 0.25 mmol), KOAc (1.91 g, 19.5 mmol), and bis(pinacolato)diboron (2.82g, 11.1 mmol). Sequential vacuum and argon were used to degas the mixture followed by the addition of degassed 1, 4 dioxane (70 mL).

The mixture was heated to 80 °C and maintained for 12 h in the dark. After the reaction, the mixture was cooled to rt and the solvent was removed under reduced pressure. The crude was partitioned between DCM (250 mL) and water (200 mL). The organic layer was separated, washed with water (2 x 200 mL), dried over anhyd. Na_2SO_4, filtered and the solvent was removed under reduced pressure to get the crude product. The crude was purified by silica chromatography using mixtures of hexanes/DCM as an eluent to obtain BPin pentacenes derivative **3** as a deep blue solid in 49% yield. 1H NMR (500 MHz, CDCl3, δ ppm): 9.34-9.32 (m, 2H), 9.30 (s, 1H), 9.27 (s, 1H), 8.51-8.507 (m, 1H), 7.98-7.95 (m, 2H), 7.93-7.90 (m, 1H), 7.72-7.70 (m, 1H), 7.42-7.40 (m, 2H), 1.44 (s, 12H) and 1.41-1.36 (m, 42H).

2.3. Synthesis of Diethynyl derivative of Oligo-(phenylene) Linkers:

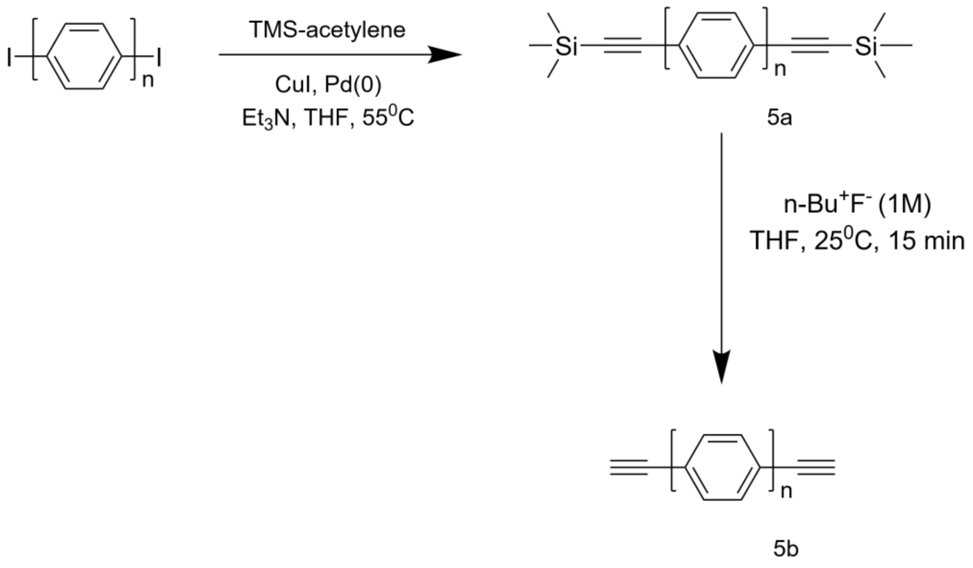

Figure S2.3. Synthesis of diethynyl derivative of linkers.

2.4. General Synthetic Procedure for Synthesis of 6,6′ Pentacene Dimes:

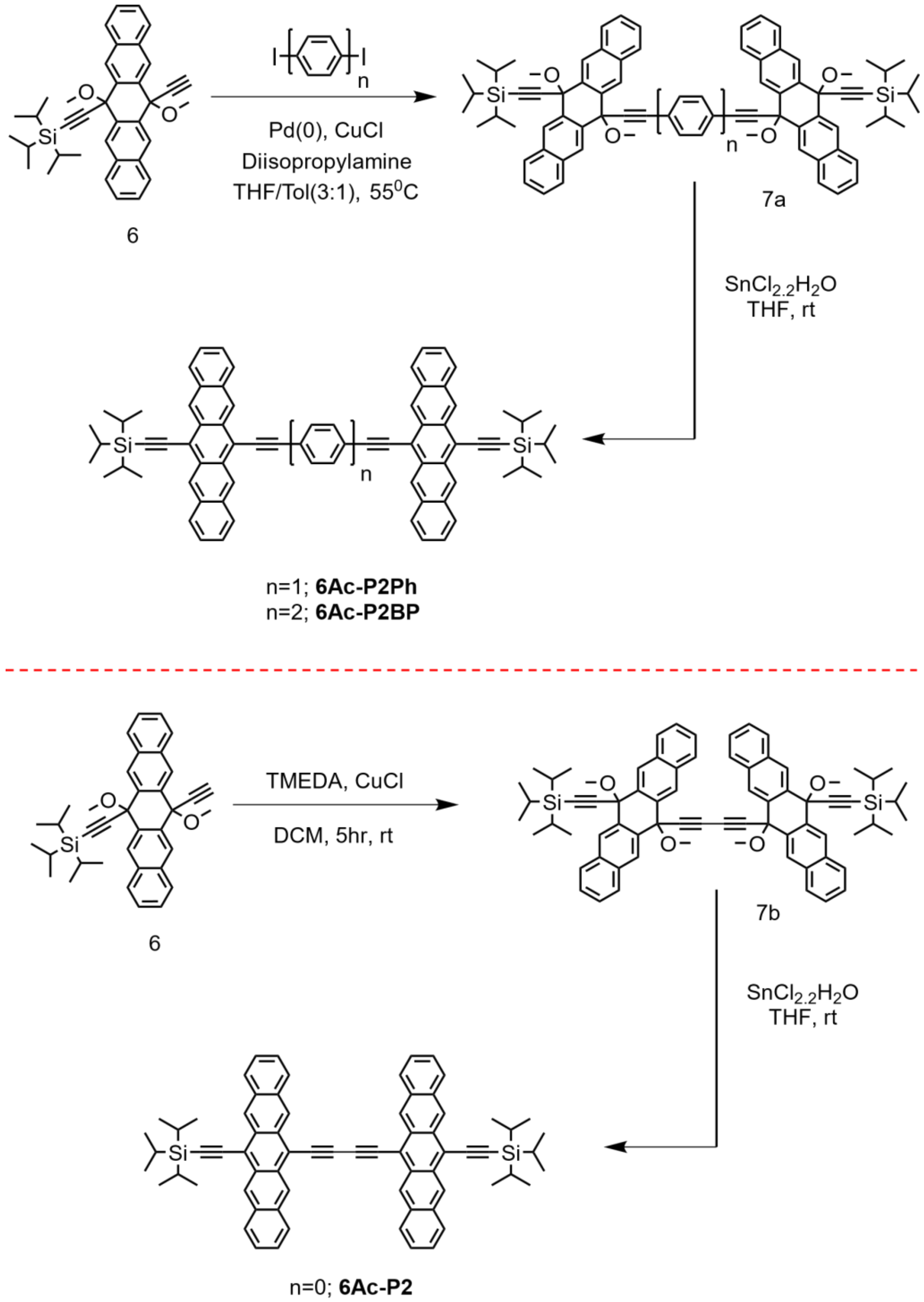

Figure S2.4. Synthesis of 6,6'-linked pentacene dimers.

The targeted molecules **6Ac-P** were synthesized according to the known procedure as described in the literature. The synthetic route and the molecular structures are illustrated in Scheme S2.4. Intermediates and final compounds were purified by silica gel chromatography; structures and purity were confirmed by ^{1}H, ^{13}C and MALDI-TOF.

2.4.1. Synthesis of 6,6′ Pentacene Dimers (6Ac-P2Ph and 6Ac-P2BP):

To a solution of **7a-b** (0.18 mmol, 1 equiv) in dry THF (12 mL) that had been deoxygenated by bubbling argon for 2 min was added $SnCl_2 \cdot 2H_2O$ (0.21g, 0.92 mmol, 5.1 equiv). The reaction flask was wrapped in aluminium foil to limit light exposure. This mixture was further deoxygenated for ca. 2 min. The solution was stirred at rt for a total of 5.5 h before pouring the mixture into MeOH (70 mL). The mixture was filtered and the solid was washed with MeOH (5 × 15 mL). The solid was suspended in CH_2Cl_2 (ca. 5 mL) and stirred for 5 min before adding hexanes (ca. 40 mL). The mixture was filtered and the solid was washed with hexanes (4 × 15 mL) to afford the analogous **6Ac-P** (yield 75-85%) as a blue-green solid.

Spectroscopic Characterisation of 6Ac-P2Ph:

Yield: 0.162g (87%). The product has low solubility, thus ^{1}H and ^{13}C NMR spectroscopy have poorly defined signals. ^{1}H NMR (500 MHz, $CDCl_3$): δ 9.28 (s, 4H), 9.27 (s, 4H), 8.07–8.04 (m, 4H), 8.03 (s, 4H), 7.99-7.95 (m, 4H), 7.44–7.40 (m, 8H), 1.40-1.36 (m, 42H). ^{13}C NMR (100 MHz, $CDCl_3$): δ 132.36, 132.04, 130.66, 130.30, 128.83, 128.68, 126.60, 126.25, 126.16, 125.94, 123.92, 118.86, 117.66, 107.57, 104.80, 104.53, 90.64, 19.12, 11.79. MALDI-MS m/z calcd. for $C_{76}H_{70}Si_2$ (M^+) 1038.501, found 1038.567.

Spectroscopic Characterisation of 6Ac-P2BP:

Yield: 0.174g (87%). The product has low solubility, thus ^{1}H and ^{13}C NMR spectroscopy have poorly defined signals. ^{1}H NMR (500 MHz, $CDCl_3$): δ 9.31-9.29 (s, 4H+4H), 8.07–8.02 (m, 8H), 7.97 (d, 4H), 7.88 (d, 4H), 7.44–7.40 (m, 8H), 1.40-1.36 (m, 42H). ^{13}C NMR (125 MHz, $CDCl_3$): δ 140.59, 132.49, 132.40 132.36, 130.74, 130.34, 128.82, 128.73, 127.37, 126.58, 126.21, 126.15, 126.05, 123.16, 118.63, 117.98, 107.45, 104.82, 104.47, 89.28, 19.11, 11.78. MALDI-MS m/z calcd. for $C_{82}H_{74}Si_2$ ($[M-H]^+$) 1113.525, found 1113.536.

Spectroscopic Characterisation of 6Ac-P2:

Yield: 0.123g (71%). The product has low solubility, thus 1H and 13C NMR spectroscopy have poorly defined signals. 1H NMR (500 MHz, CDCl3): δ 9.39 (s, 4H), 9.34 (s, 4H), 8.16-8.13 (m, 4H), 8.02-7.98 (m, 4H), 7.47-7.43 (m, 8H), 1.40-1.37 (m, 42H). 13C NMR (125 MHz, CDCl3): δ 132.82, 132.51, 131.59, 130.59, 128.85, 128.73, 126.97, 126.58, 126.36, 125.94, 120.00, 116.40, 108.65, 104.69, 89.76, 86.52, 17.12, 11.77. MALDI-MS m/z calcd. for C70H66Si2 (M+) 962.470, found 962.173.

2.4.2. Synthesis of 6,6′ Pentacene Dimer Precursor (7a):

To a solution of **6** (0.400 g, 0.74 mmol, 2 equiv) and diiodo derivative of the linker (0.37 mmol, 1 equiv) in dry THF and toluene mixture (3:1, 15mL) and diisopropylamine (5.0 mL, 3.6 g, 36 mmol) which had been deoxygenated for 10 min with argon was added $Pd(PPh_3)_4$ (0.030 g, 0.026 mmol) and CuCl (0.010 g, 0.10 mmol). The reaction mixture was further deoxygenated with argon for an additional 2 min. The reaction mixture was stirred for 24 h at 55 °C, cooled to rt, and poured into satd. aq. NH_4Cl (100 mL). H_2O (150 mL) was added, and the mixture was extracted with CH_2Cl_2 (80 mL, 50 mL). The organic phase was washed with 5% NH_4Cl (200 mL), dried (Na_2SO_4), filtered, and the solvent removed in vacuo. Column chromatography (silica gel, 15:2 hexanes/EtOAc) afforded **7a** (yield 55-65%) as an off white foamy solid.

Spectroscopic Characterisation of 7a (n=1):

Yield: 0.271g (63%). ^1H NMR (500 MHz, $CDCl_3$): δ 8.69 (s, 4H), 8.40 (s, 4H), 7.94-7.92 (m, 4H), 7.90-7.87 (m, 4H), 7.53-7.50 (m, 8H), 7.11 (s, 4H), 3.06 (s, 6H, OCH_3), 3.00 (s, 6H, OCH_3), 1.25-1.22 (m, 42H).

Spectroscopic Characterisation of 7b (n=2):

Yield: 0.289g (63%). ^1H NMR (500 MHz, $CDCl_3$): δ 8.71 (s, 4H), 8.45 (s, 4H), 7.98-7.95 (m, 4H), 7.92-7.89 (m, 4H), 7.55-7.52 (m, 8H), 7.33 (br, 8H), 3.09 (s, 6H, OCH_3), 3.05 (s, 6H, OCH_3), 1.25-1.22 (m, 42H).

2.4.3. Synthesis of 6,6′ Pentacene Dimer Precursor (7b):

To a suspension of CuCl (0.040 g, 0.40 mmol) in TMEDA (1.1 mL, 0.85 g, 7.3 mmol) was added dry CH2Cl2 (10 mL). This suspension was oxygenated by bubbling O2 for approx. 2 min. To this mixture was added a solution of **1** (0.200 g, 0.367 mmol) in dry CH_2Cl_2 (3 mL). The reaction mixture was allowed to stir 4 h at rt open to the atmosphere and then poured into satd. aq. NH_4Cl (200 mL). The mixture was extracted with CH_2Cl_2 (2 × 50 mL). The combined organic phase was washed with H2O (200 mL, 150 mL), satd. aq. NaCl (150 mL), dried (Na_2SO_4), and the solvent removed in vacuo. Column chromatography (silica gel, 4:1 CH2Cl2/hexanes) afforded the product (0.119 g, 60%) as a blue-green solid.

Spectroscopic Characterisation of 7b (n=0):

Yield: 0.289g (63%). 1H NMR (500 MHz, $CDCl_3$): δ 8.60 (s, 4H), 8.30 (s, 4H), 7.89-7.85 (m, 4H), 7.87-7.84 (m, 4H), 7.51-7.48 (m, 8H), 2.96 (s, 6H, OCH3), 2.92 (s, 6H, OCH3), 1.17-1.14 (m, 42H).

2.4.4. Synthesis of Pentacene Precursor for 6,6′-linked Pentacene Dimers:

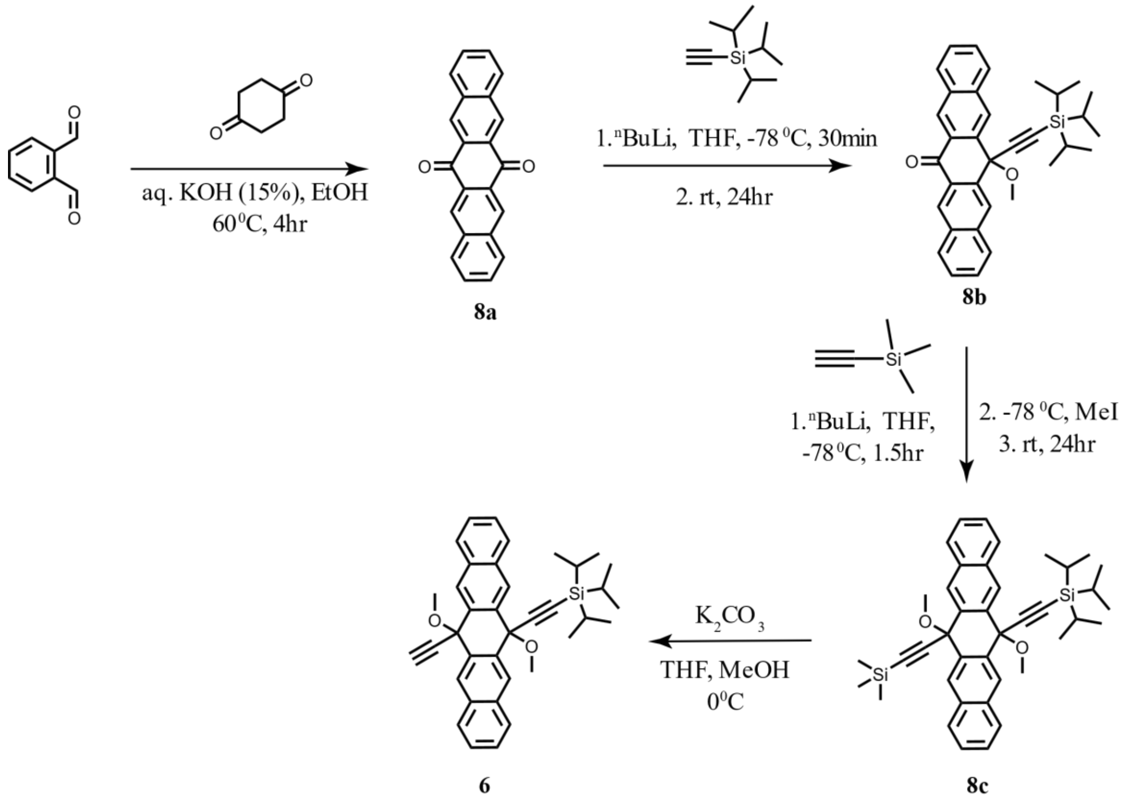

Figure S2.5. Synthesis of Pentacene precursor for 6,6’-linked pentacene dimers.

Compound 6 was synthesized according to procedure reported in the literature.[6,7]

Synthesis and Spectroscopic Characterisation of pentacene-6,13-quinone (8a): 6.20 g (46.2 mmol) of ortho-phthaldialdehyde and 2.50 g (22.3 mmol) of cyclohexanedione were dissolved in 250 mL of ethanol. 20 mL of a 15% aqueous KOH solution was added, upon which the solution turned brownish, and a yellow/orange precipitate formed. For completion of the reaction the mixture was stirred for an additional 4 h at rt. After cooling the solid was filtered and thoroughly washed with acetone, giving the product as a yellow solid (6.125 g, 19.8 mmol; 90 %). The product was carried forward to the next step as such. ^{1}H NMR (500 MHz, $CDCl_3$): δ 8.89 (s, 4H), 8.08-8.06 (dd, 4H), 7.66-7.64 (dd, 4H).

Synthesis and Spectroscopic Characterisation of 8b:

To a solution of tri-isopropylsilylacetylene (4.25 g, 23.3 mmol) in THF (30 mL) cooled to -78 °C was added dropwise ^{n}BuLi (1.6 M in hexanes, 12.5 mL, 20 mmol). The solution was allowed to stir for 15 min before being transferred slowly via cannula into a suspension of 6,13- pentacenequinone (6.16 g, 20 mmol) in THF (70 mL) at rt. The reaction mixture was stirred for 24 h at rt. The reaction was cooled to –15 °C and quenched via the addition of satd. aq. NH_4Cl (1 mL). The suspension was filtered and the solid was washed with 1:1 THF/water (3 × 4 mL), then THF (3 × 4 mL). This allowed for the recovery of the excess 6,13-pentacenequinone, which could be used in subsequent reactions after drying under high vacuum. The filtrate was collected into a filter flask which already contained satd. aq. NH_4Cl (100 mL), and after filtration and mixing, the mixture was extracted with CH_2Cl_2 (60 mL, 40 mL). The combined organic phase was washed with satd. aq. NaCl (100 mL), dried (Na_2SO_4), and the solvent removed in vacuo to provide a solid orange residue. This solid was redissolved in minimum volume CH_2Cl_2 and precipitated by adding hexanes (100 mL) and cooling to -78 °C. The yellow-coloured solid was collected by vacuum filtration and washed with cold hexanes (3 × 4 mL). A second crop of product was obtained from the filtrate and combined with the first crop. The solid was redissolved in acetone and filtered to remove insoluble impurities. The solvent of the filtrate was removed in vacuo to afford 4b (8.85 g, 90%) as a yellow solid. ^{1}H NMR (500 MHz, $CDCl_3$): δ 8.84 (s, 2H), 8.73 (s, 2H), 8.06-8.04 (d, J = 8 Hz, 2H), 7.94-7.93 (d, J = 8 Hz, 2H), 7.66-7.63 (app t, J = 7.5 Hz, 2H), 7.60-7.57 (app t, J = 7.5 Hz, 2H), 2.99 (s, 1H), 1.17–1.15 (m, 21H). ^{13}C NMR (125 MHz, $CDCl_3$): δ 184.4, 138.7, 135.6, 132.8, 129.8, 129.7, 128.9, 128.5, 128.2, 127.4, 127.3, 108.2, 90.1, 68.7, 18.7, 11.3.

Synthesis and Spectroscopic Characterisation of 8c:

To a solution of trimethylsilylacetylene (3.0 mL, 2.108 g, 21.46 mmol) in THF (20 mL) cooled to -78 °C was added slowly ^{n}BuLi (1.6 M in hexanes, 11.5 mL, 18.36 mmol). The solution was allowed to stir for 15 min before being transferred via cannula into a solution of 4b (3 g, 6.12 mmol) in THF (30 mL) cooled to -78 °C. The reaction mixture was allowed to stir at -78 °C for 15 min before removing the

cooling bath and allowing the solution to warm to 0 °C and stir for 2.5 h. The solution was cooled to -78 °C and MeI (3.8 mL, 8.68 g, 61.2 mmol) was added slowly. The reaction flask was wrapped in aluminium foil to limit light exposure and the mixture was allowed to warm to rt and stir for 24 h. The mixture was cooled to 0 °C and poured into satd. aq. NH_4Cl (150 mL). H_2O (150 mL) was added, and the mixture was extracted with CH_2Cl_2 (2 × 50 mL). The combined organic phase was washed with H_2O (200 mL), satd. aq. NaCl (150 mL), dried (Na_2SO_4), and the solvent removed in vacuo. Column chromatography (silica gel, 1:1 CH_2Cl_2/hexanes) afforded 4c (2.9 g, 78%) as an off white solid. ^1H NMR (500 MHz, $CDCl_3$): δ 8.68 (s, 2H), 8.41 (s, 2H), 7.98–7.95 (m, 2H), 7.92–7.89 (m, 2H), 7.56–7.51 (m, 4H), 3.01 (s, 3H), 3.00 (s, 3H), 1.26–1.20 (m, 21H), 0.06 (s, 9H). ^{13}C NMR (125 MHz, $CDCl_3$): δ 134.0, 133.5, 133.3, 132.7, 128.2, 128.1, 128.0, 126.9, 126.7, 126.6, 106.7, 105.5, 91.6, 90.2, 75.9, 73.6, 51.9, 51.8, 18.7, 11.4, - 0.2.

Synthesis and Spectroscopic Characterisation of 6:

To a solution of 4c (4.88 g, 7.91 mmol) in THF (100 mL) and MeOH (100 mL) cooled to 0 °C was added K_2CO_3 (1.21 g, 8.75 mmol). The reaction mixture was maintained between 0 °C and 5 °C and stirred for 5.5 h and then poured into satd. aq. NH_4Cl (200 mL). H2O (50 mL) was added, and the mixture was extracted with hexanes (80 mL, 2 × 50 mL). The combined organic phase was washed with satd. aq. NH_4Cl (200 mL), satd. aq. $NaHCO_3$ (200 mL), satd. aq. NaCl (150 mL), dried (Na_2SO_4), and the solvent removed in vacuo. The rather pure oil was was redissolved in minimal CH_2Cl_2 (ca. 2 mL) and precipitated through the addition of MeOH (80 mL) and cooling to -78 °C. The solid was washed with cold MeOH (3 × 15 mL) to afford 1 (3.81 g, 88%) as an off white solid. ^1H NMR (500 MHz, $CDCl_3$): δ 8.70 (s, 2H), 8.43 (s, 2H), 7.99–7.94 (m, 2H), 7.94–7.89 (m, 2H), 7.57–7.51 (m, 4H), 3.04 (s, 3H), 3.02 (s, 3H), 2.69 (s, 1H), 1.28–1.21 (m, 21H). ^{13}C NMR (125 MHz, $CDCl_3$): δ 133.52, 133.50, 133.1, 132.9, 128.3, 128.2, 128.1, 126.93, 126.89, 126.7, 105.2, 92.1, 85.9, 76.0, 73.7, 72.9, 51.9, 51.8, 18.8, 11.4.

3. Computational Methods

Calculations were done using Density Functional Theory (DFT). The lowest energy conformers in the ground state were calculated with the CAM-B3LYP[2] functional and the 6- 31G(d)[3] basis set using the Gaussian program package.[1] All states were found using DFT. The basis set size is kept nominal due to the large number of atoms. The presence of only real frequencies confirmed that these were indeed minima on the respective potential energy surfaces. The CAM-B3LYP functional is chosen based on its more accurate estimate of charge transfer excitations in acenes which B3LYP underestimates enormously.[2] Since the pentacene dimers discussed in this work are systems in which each monomer is covalently connected through linkers and is mainly affected by through-bond interactions, we have chosen a simple basis set without diffuse function.[8,9]

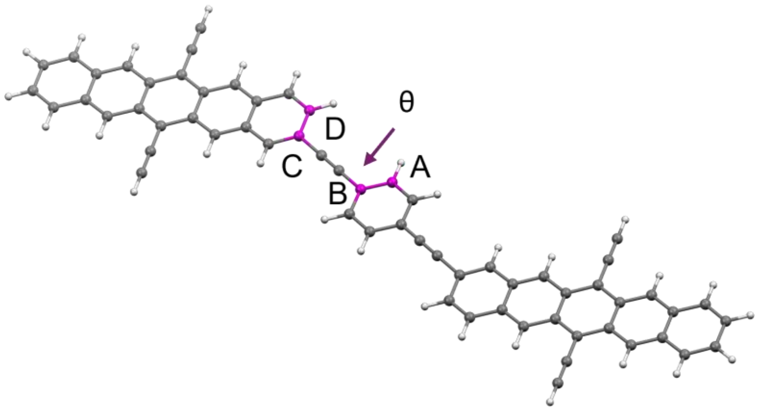

The same functional and basis set was used in the potential energy scan over the dihedral angle between the pentacene planes and the plane of the (oligo)phenylene linkers (θ) as to estimate the rotational energy barrier along the acetylene linkage as shown in Figure 2 to estimate the rotational energy barrier along the biphenyl axis. The potential energy of each conformer was calculated in a relaxed optimization where the angle θ is fixed and all other atomic coordinates are allowed to relax to find the lowest energy of the system. No symmetry constraints were used.

3.1.1. Structural parameters

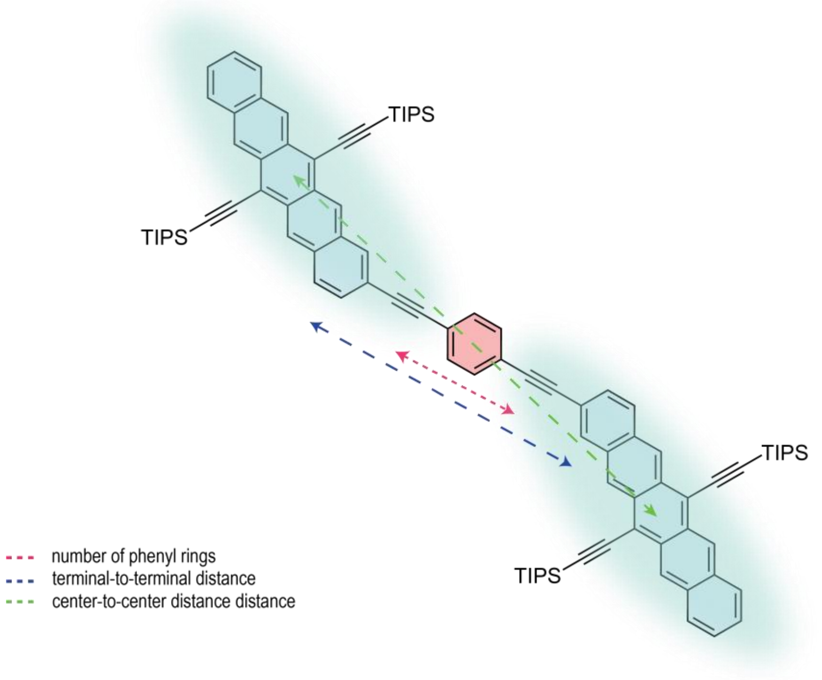

Figure S3.1.1. Pentacene-to-pentacene interchromophore distances. Center-to-center (green) defines the interchromophore distance between the central rings of each pentacene. Edge-to-edge (red) defines the interchromophore distance between the effective chromophore units depending on the extent of singlet density delocalisation. Terminal-to-terminal (blue) defines the interchromophore distance between the terminal rings of each pentacene.

Compounds		Interpentacene distance (Å) (centre-to-centre)	Interpentacene distance (Å) (terminal-to-terminal)	θ (°)	φ((°) biphenyl torsion
**6Ac-P**	**6Ac-P2**	9.47	9.47	0	NA
	**6Ac-P2Ph**	13.77	13.77	0	NA
	**6Ac-P2BP**	18.09	18.09	0	37
**2-P**	**2-P2**	13.62	4.33	40	NA
	**2-P2Ph**	17.81	8.64	37	NA
	**2-P2BP**	21.94	12.95	37	37
**2Ac-P**	**2Ac-P2**	18.64	9.4	0	NA
	**2Ac-P2Ph**	22.82	13.77	0	NA
	**2Ac-P2BP**	27.03	18.09	0	37

Table S3.1.1 Structural parameters of optimized geometries for **6Ac-P**, **2-P** and **2Ac-P** pentacene dimers.

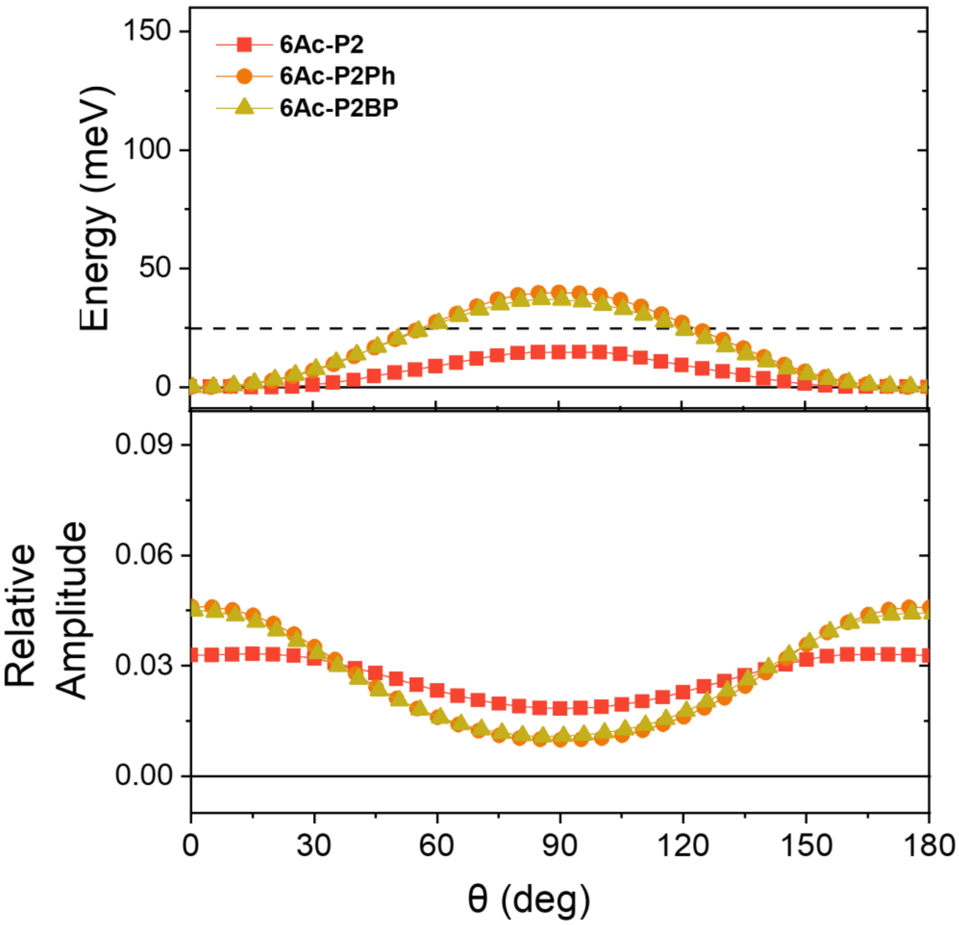

Figure S3.1.2 Calculated potential energy curves (top) and relative probabilities (bottom) along the θ coordinate in the ground state of **6Ac-P** dimers. The horizontal dashed lines in the top panels denote the room temperature energy.

3.2. Energy level splitting:

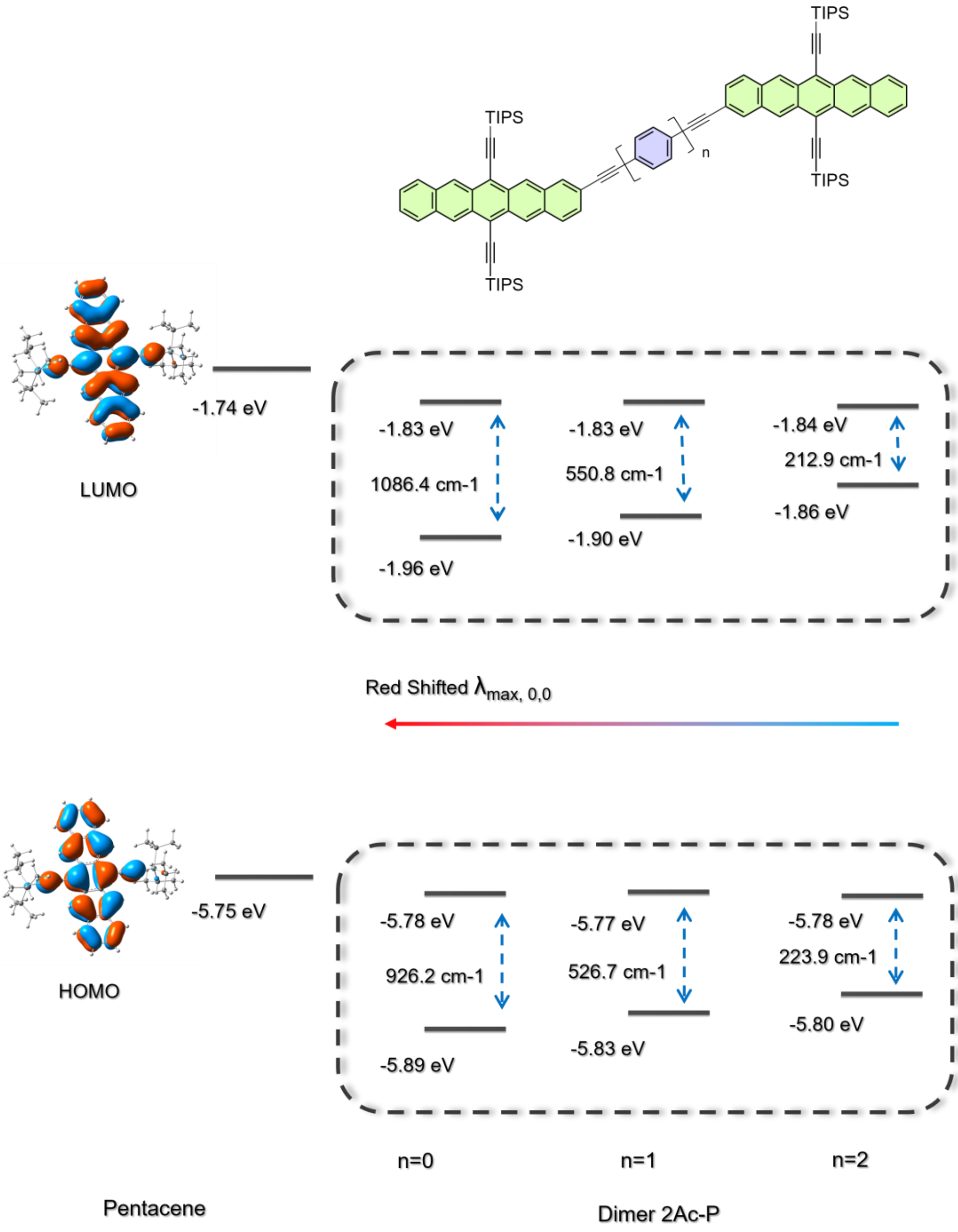

Figure S3.2.1 Energy-level splitting between LUMO+1 and LUMO (ΔE_{LUMO}) and HOMO and HOMO-1 (ΔE_{HOMO}) **2Ac-P** dimers.

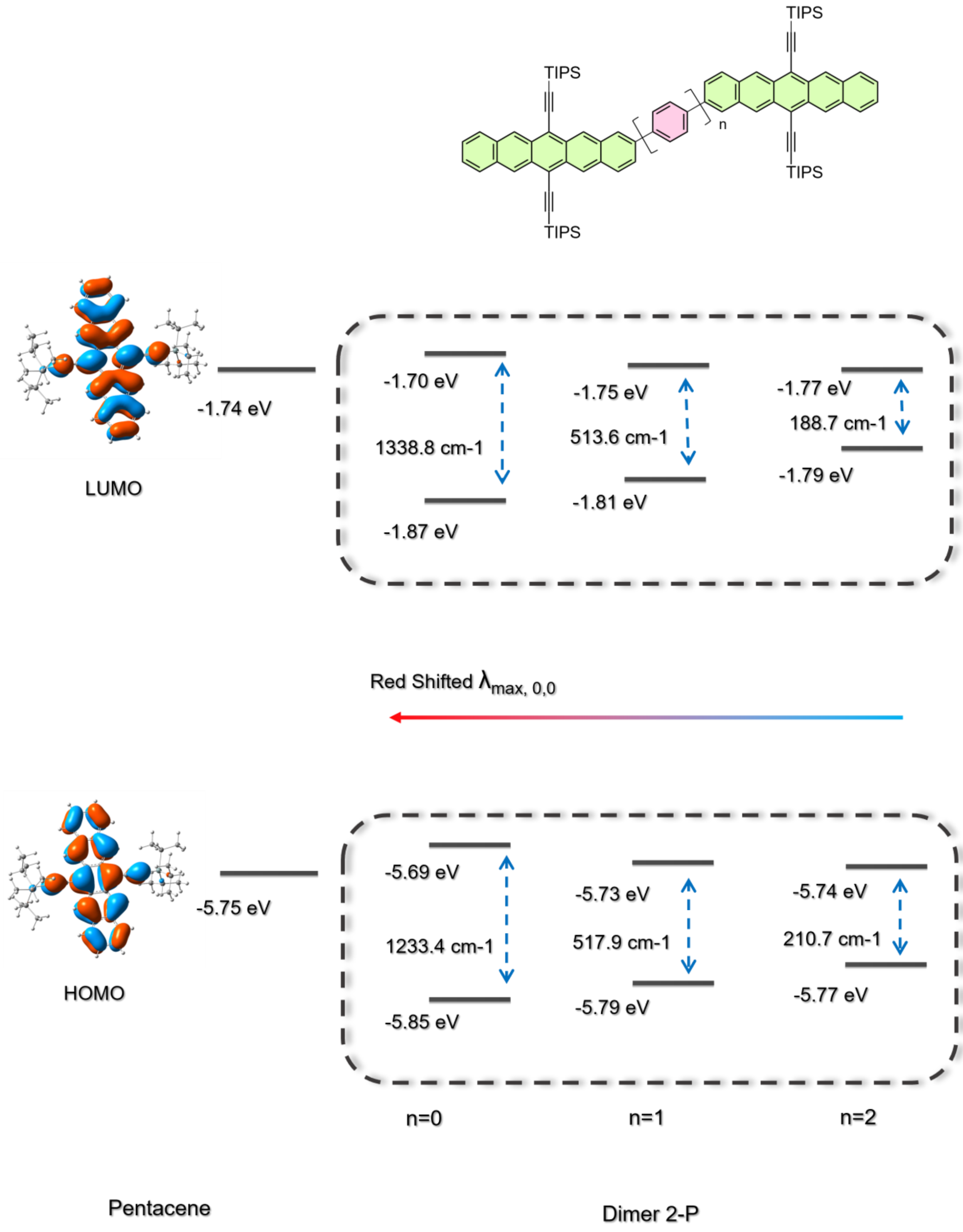

Figure S3.2.2 Energy-level splitting between LUMO+1 and LUMO (ΔE_{LUMO}) and HOMO and HOMO-1 (ΔE_{HOMO}) **2-P** dimers.

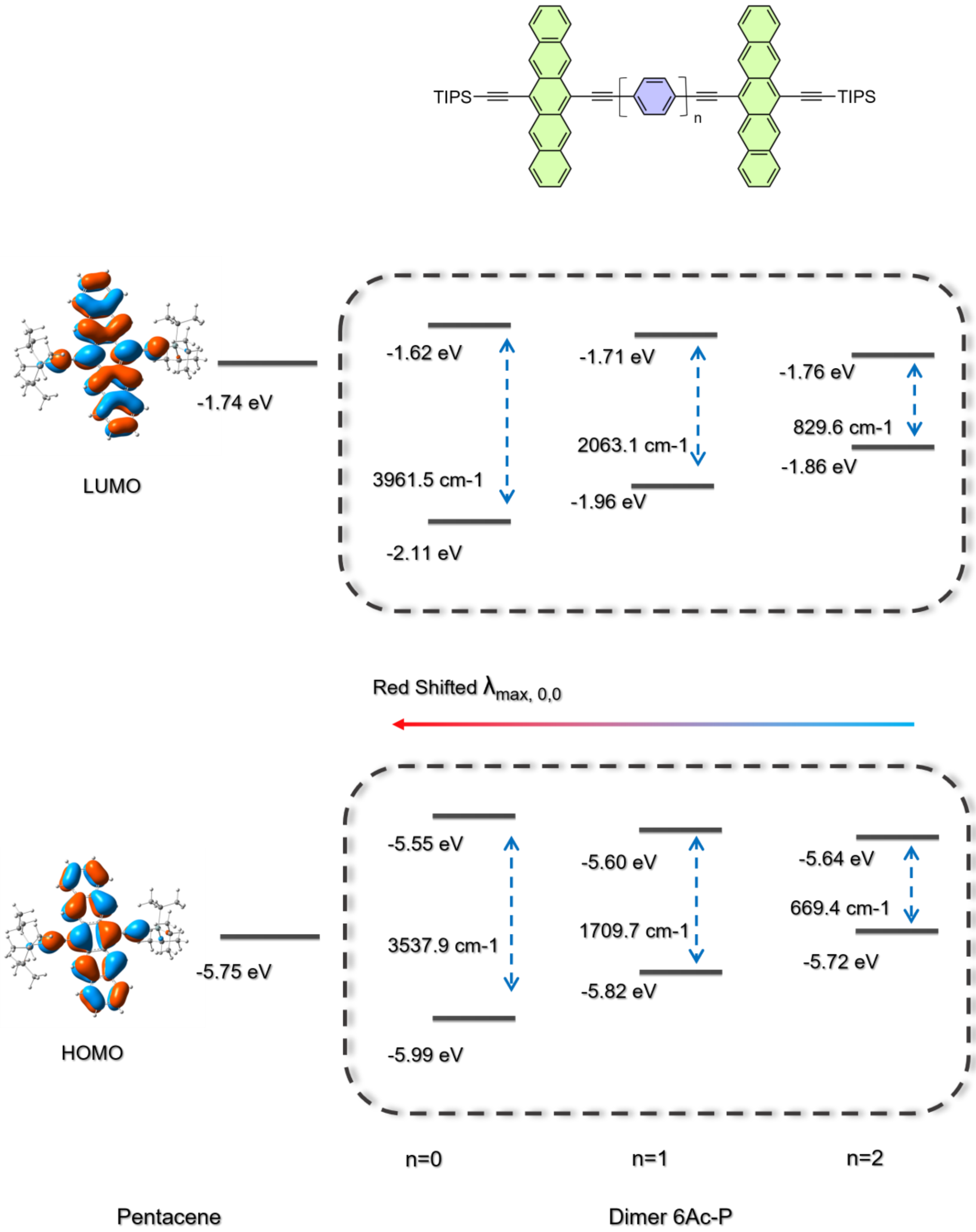

Figure S3.2.3 Energy-level splitting between LUMO+1 and LUMO (ΔE_{LUMO}) and HOMO and HOMO-1 (ΔE_{HOMO}) **6Ac-P** dimers.

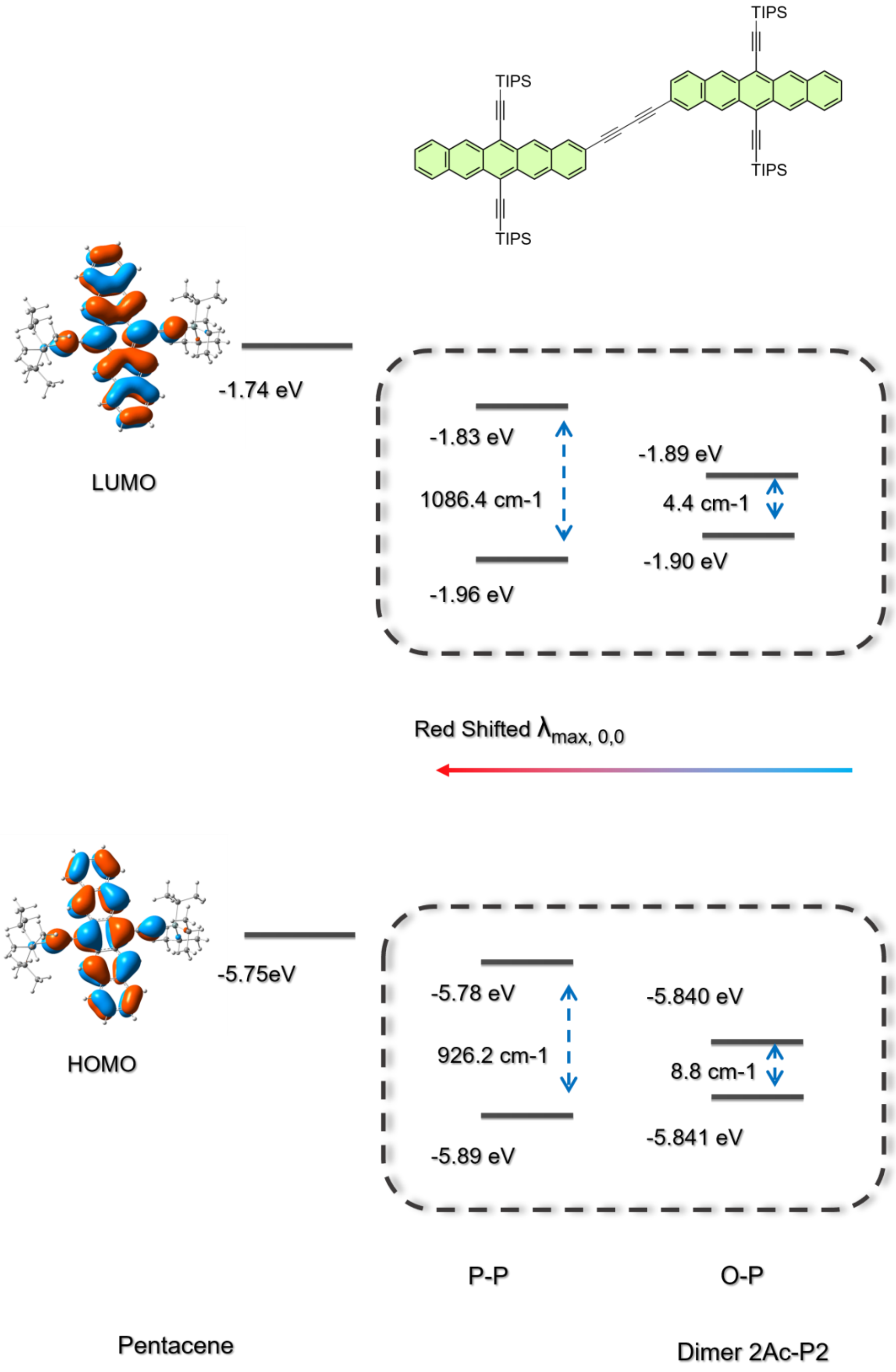

Figure S3.2.4 Energy-level splitting between LUMO+1 and LUMO (ΔE_{LUMO}) and HOMO and HOMO-1 (ΔE_{HOMO}) in rotational conformers (P-P, O-P) of **2Ac-P2** dimer (DOI: 10.1021/jacs.3c06075). The P-P conformer represents the conformer when the two pentacenes are on the same plane and the same plane with the bridge. The O-P conformer represents the conformer when one of the pentacene is orthogonal to the plane of the other and the bridge. The O-O conformer represents the conformer where both the pentacenes are orthogonal to the bridge plane but are parallel to each other.

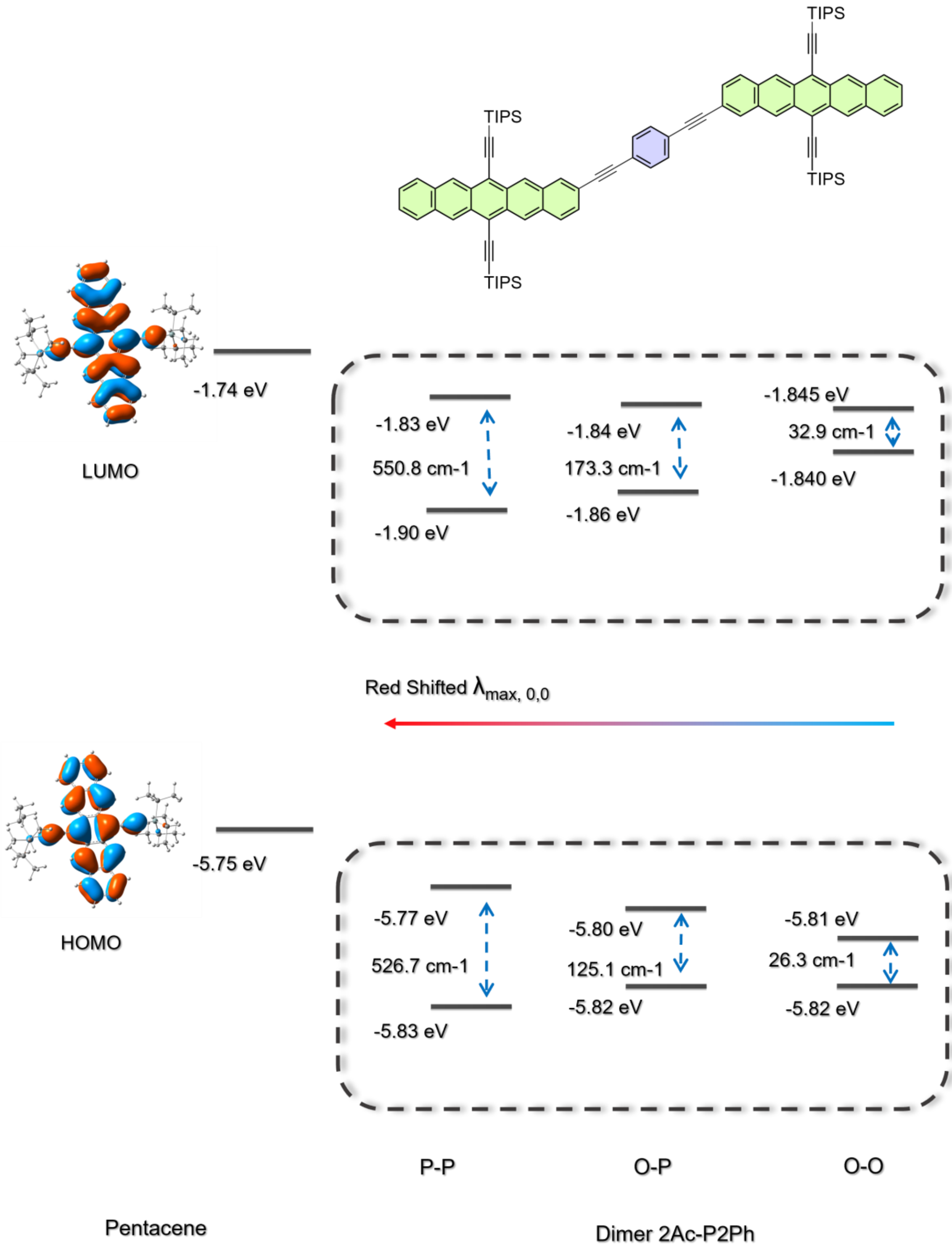

Figure S3.2.5 Energy-level splitting between LUMO+1 and LUMO (ΔE_{LUMO}) and HOMO and HOMO-1 (ΔE_{HOMO}) in rotational conformers (P-P, O-P, O-O) of **2Ac-P2Ph** dimer.

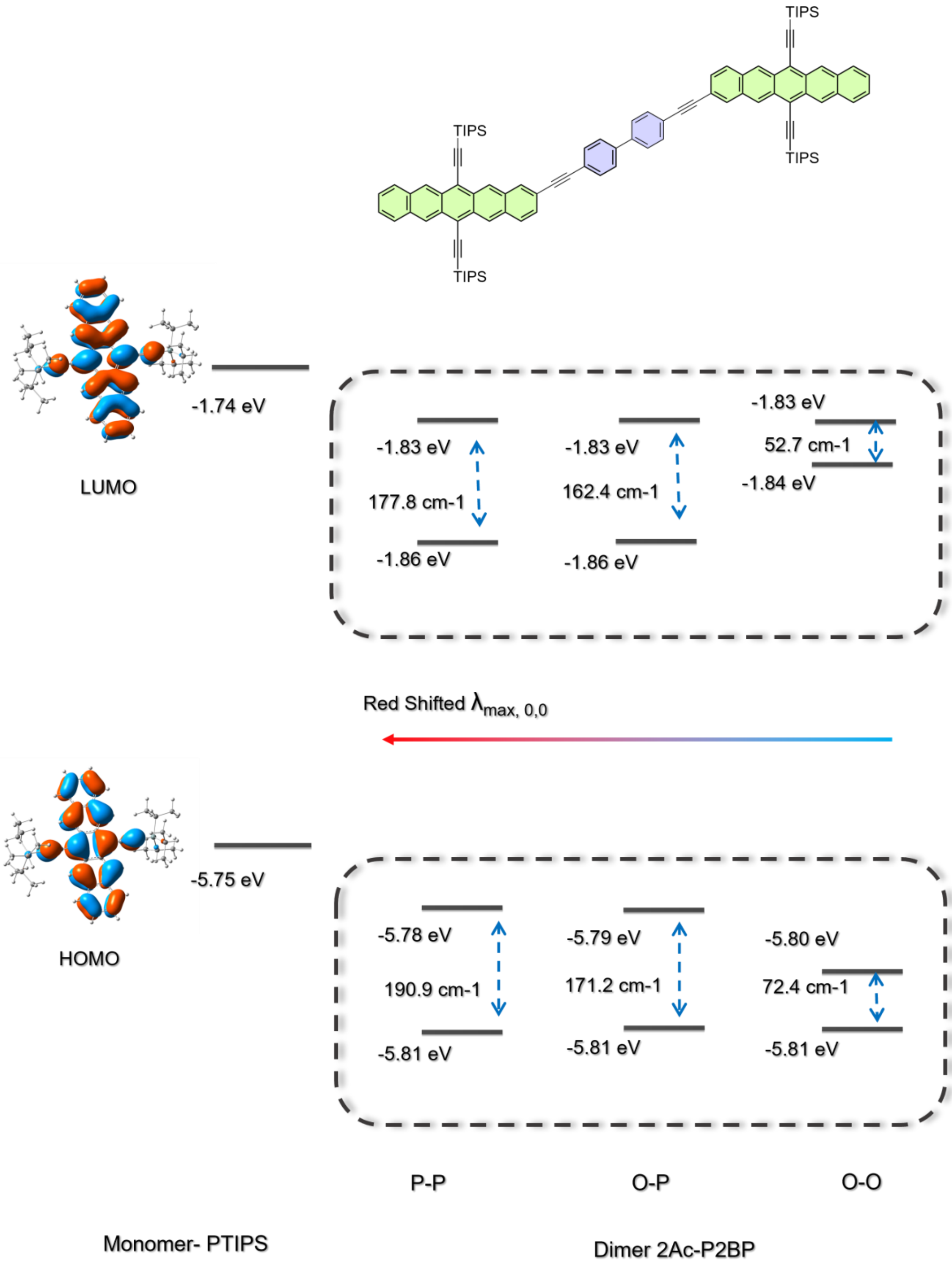

Figure S3.2.6 Energy-level splitting between LUMO+1 and LUMO (ΔE_{LUMO}) and HOMO and HOMO-1 (ΔE_{HOMO}) in rotational conformers (P-P, O-P, O-O) of **2Ac-P2BP** dimer.

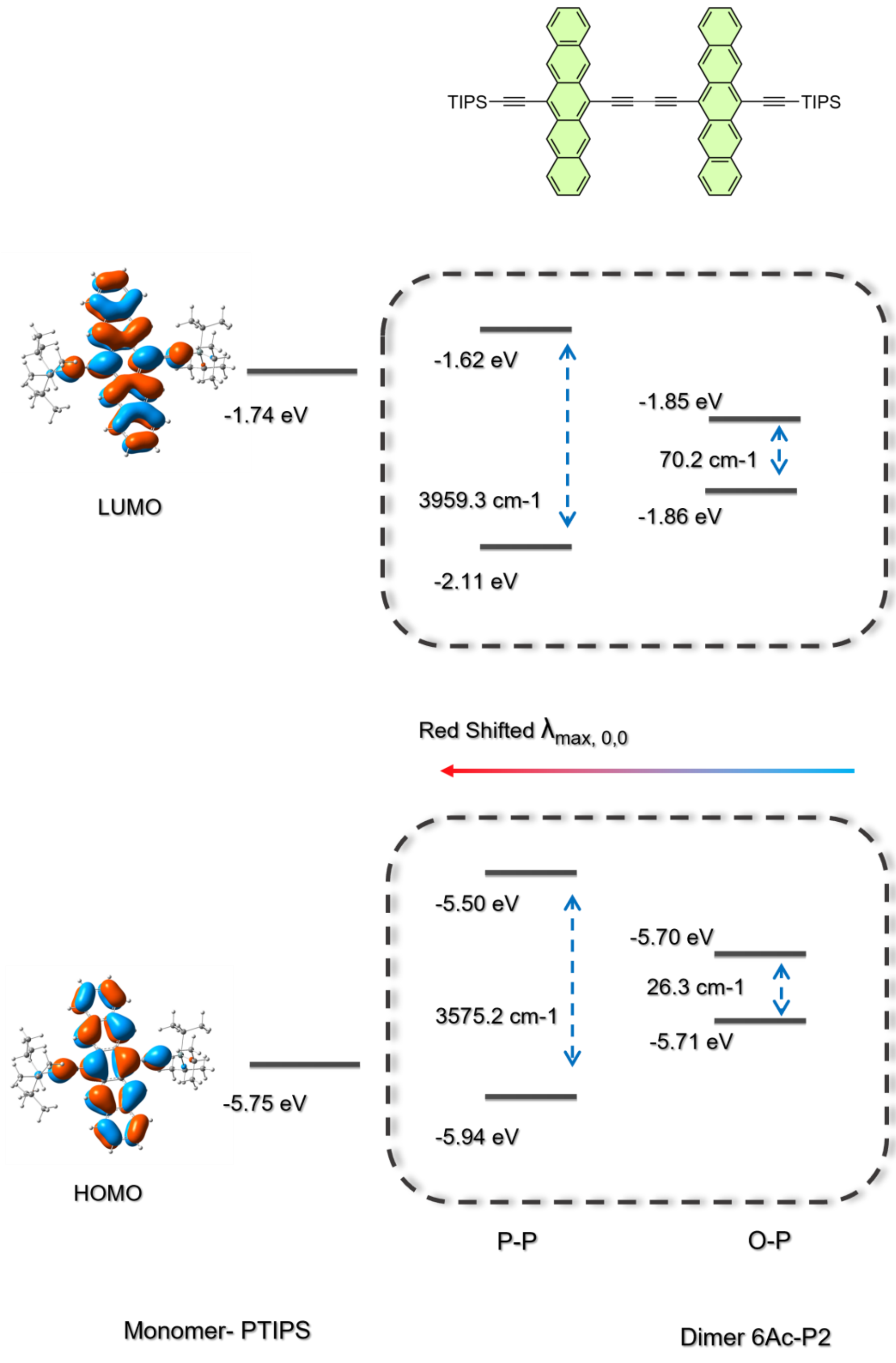

Figure S3.2.7 Energy-level splitting between LUMO+1 and LUMO (ΔE_{LUMO}) and HOMO and HOMO-1 (ΔE_{HOMO}) in rotational conformers (P-P, O-P) of **6Ac-P2** dimer.

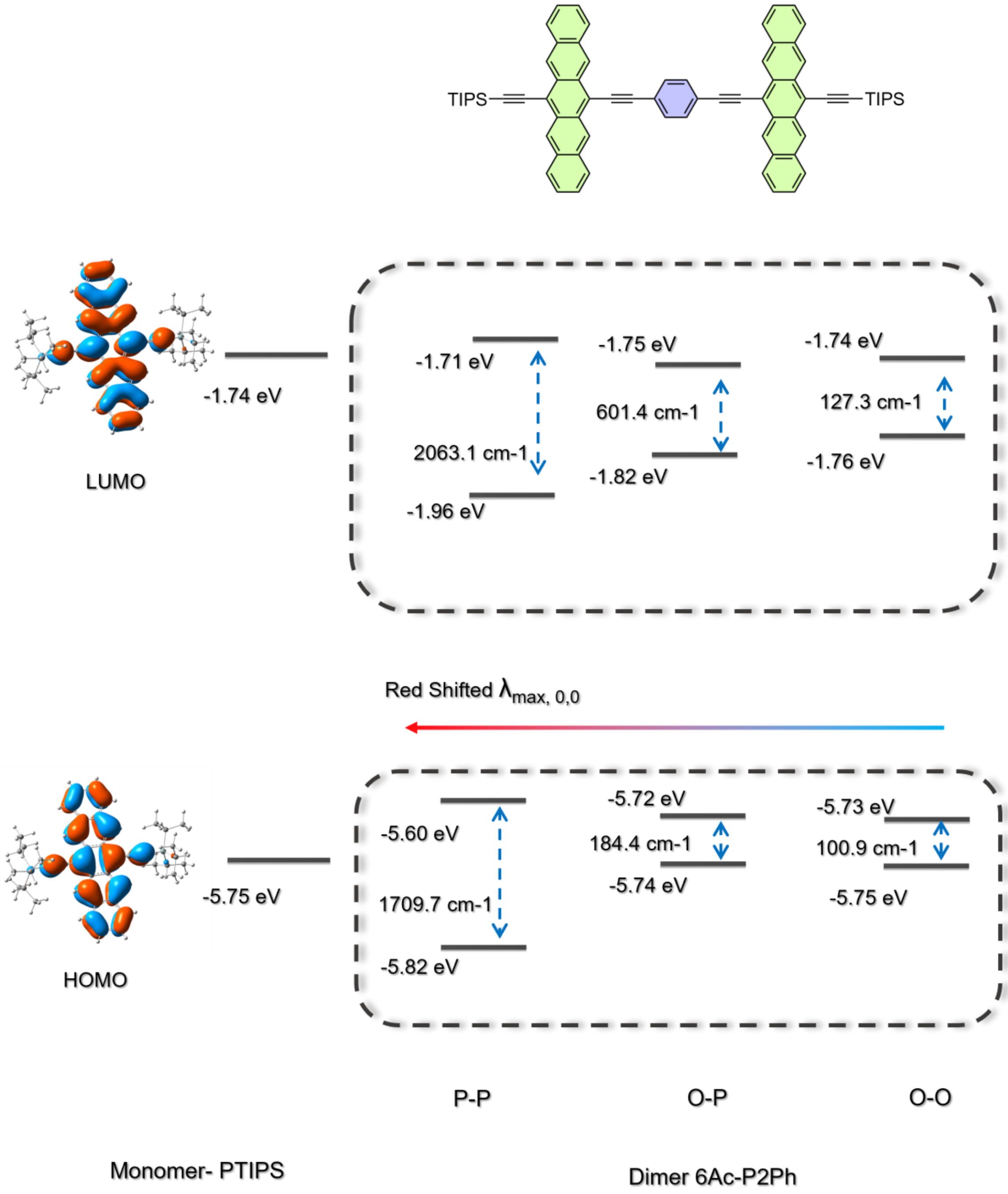

Figure S3.2.8 Energy-level splitting between LUMO+1 and LUMO (ΔE_{LUMO}) and HOMO and HOMO-1 (ΔE_{HOMO}) in rotational conformers (P-P, O-P, O-O) of **6Ac-P2Ph** dimer.

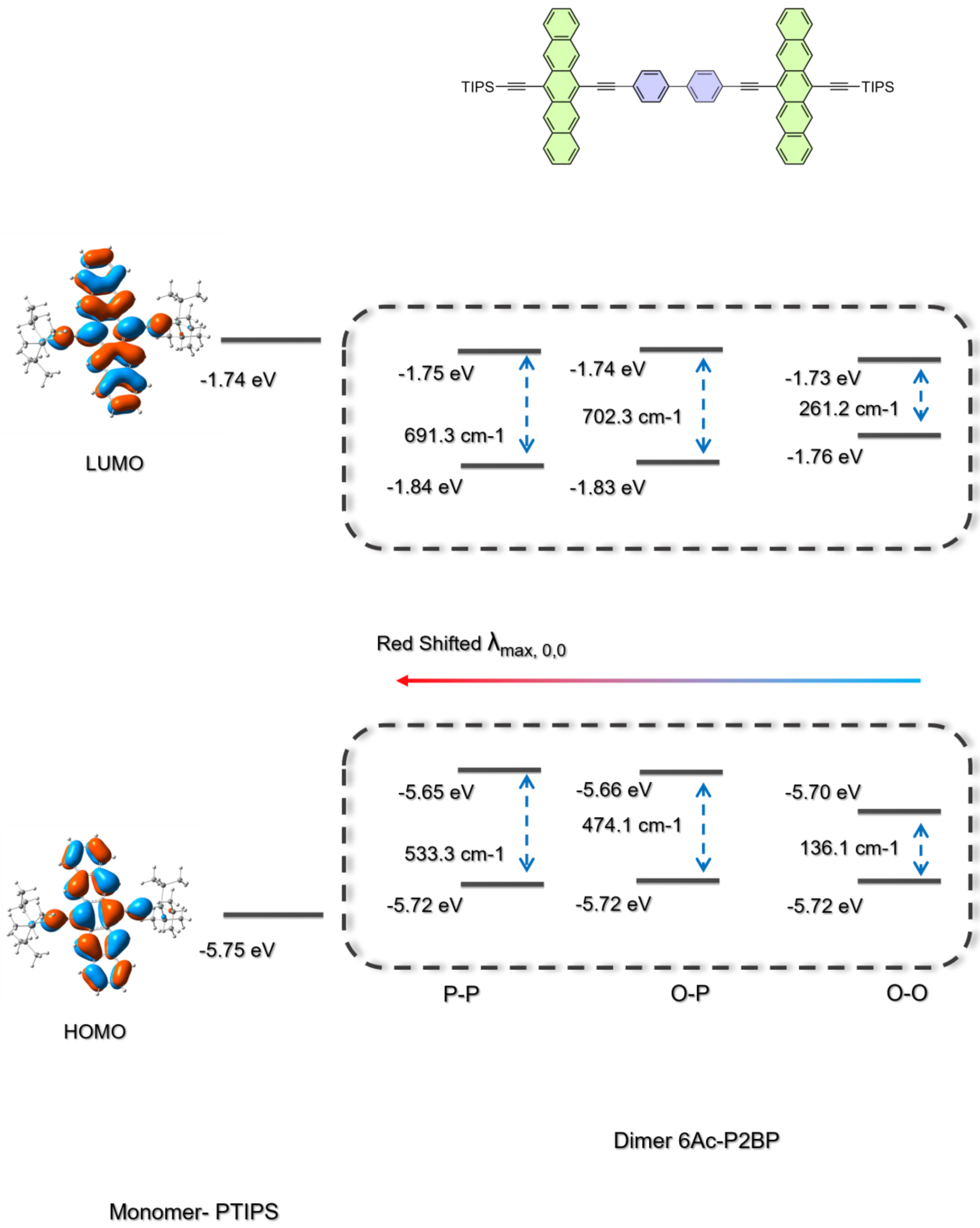

Figure S3.2.9 Energy-level splitting between LUMO+1 and LUMO (ΔE_{LUMO}) and HOMO and HOMO-1 (ΔE_{HOMO}) in rotational conformers (P-P, O-P, O-O) of **6Ac-P2BP** dimer.

3.3. Ground State Optimised Geometries

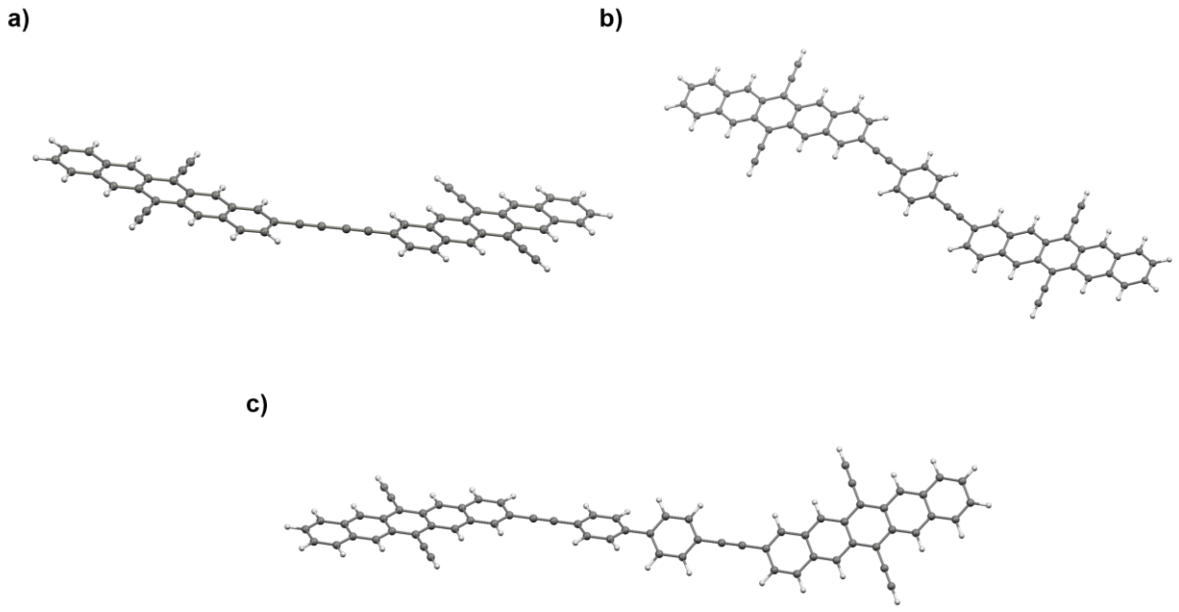

Figure S3.3.1. Ground state optimised geometries of **a) 2Ac-P2, b) 2Ac-P2Ph** and **c) 2Ac-P2BP**. 2Ac-P2 and 2Ac-P2Ph are completely planar but 2Ac-P2BP has a twist of 37^0 because of the biphenyl moiety.

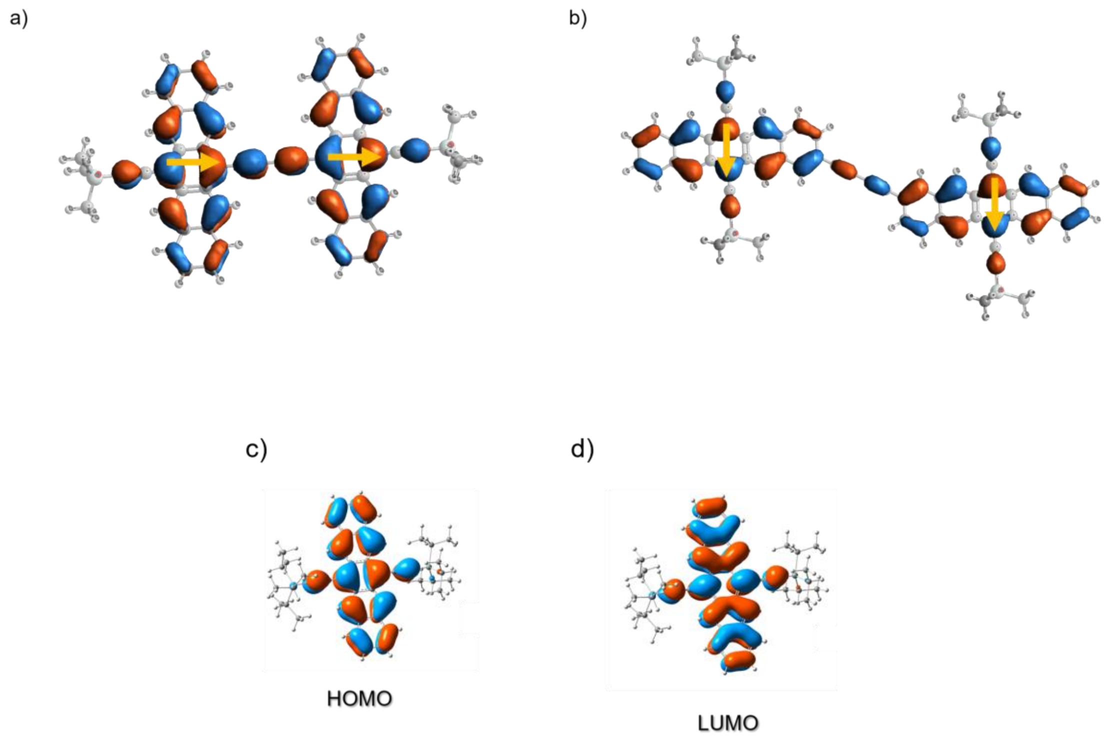

Figure S3.3.2. a) b) Alignment of $S_0 \rightarrow S_1$ transition dipole moment in 6,6'-linked and 2,2'-linked pentacene dimers giving rise to head to tail J type and slipped parallel H type coupling. c) d) HOMO and LUMO of P-TIPS showing the orbital density at both 6 and 2 carbon centres.

3.4. Triplet Density and Molecular Orbitals

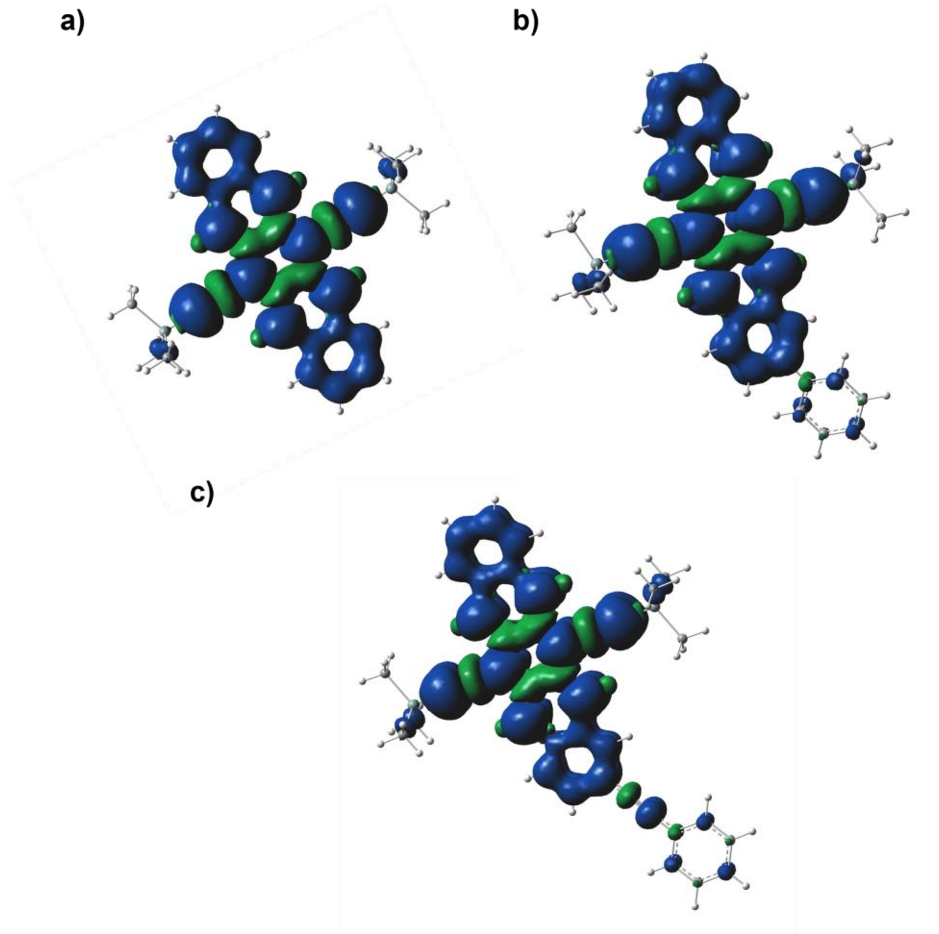

Figure S3.4.1. Triplet spin density distribution in **a) PTIPS, b) 2-PTIPS-Ph** and **c) 2Ac-PTIPS-Ph**.

LUMO

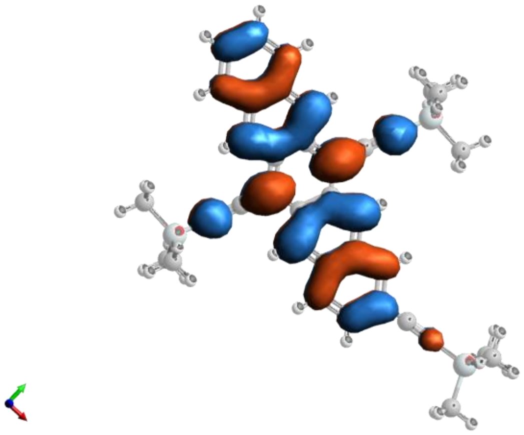

HOMO

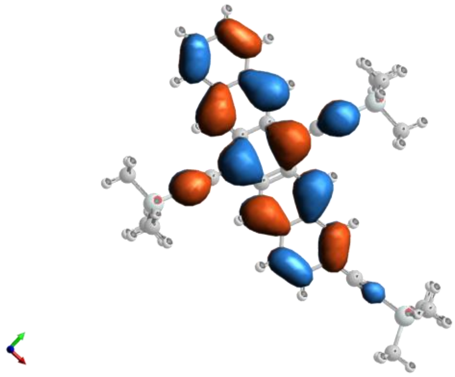

Figure S3.4.2. Frontier molecular orbitals of **2Ac-PTIPS.**

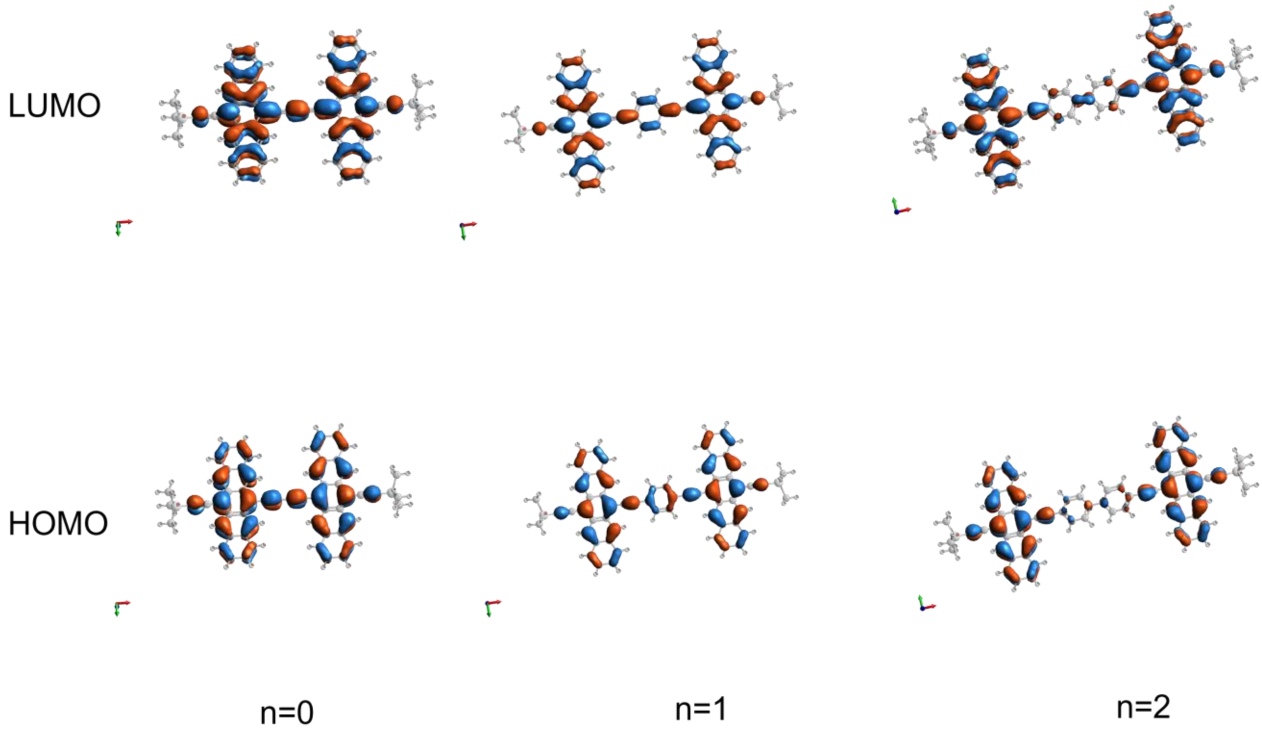

Figure S3.4.3. Frontier molecular orbitals of **6Ac-P2**, **6Ac-P2Ph** and **6Ac-P2BP**.

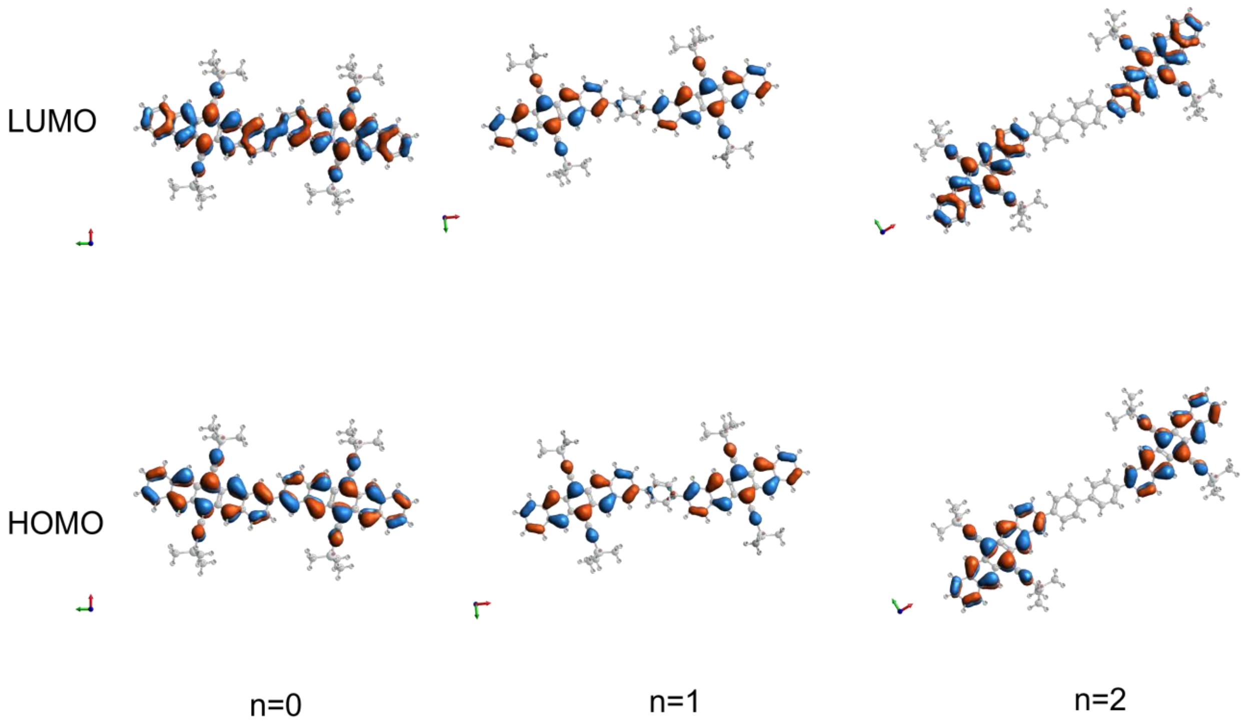

Figure S3.4.4. Frontier molecular orbitals of **2-P2**, **2-P2Ph** and **2-P2BP**.

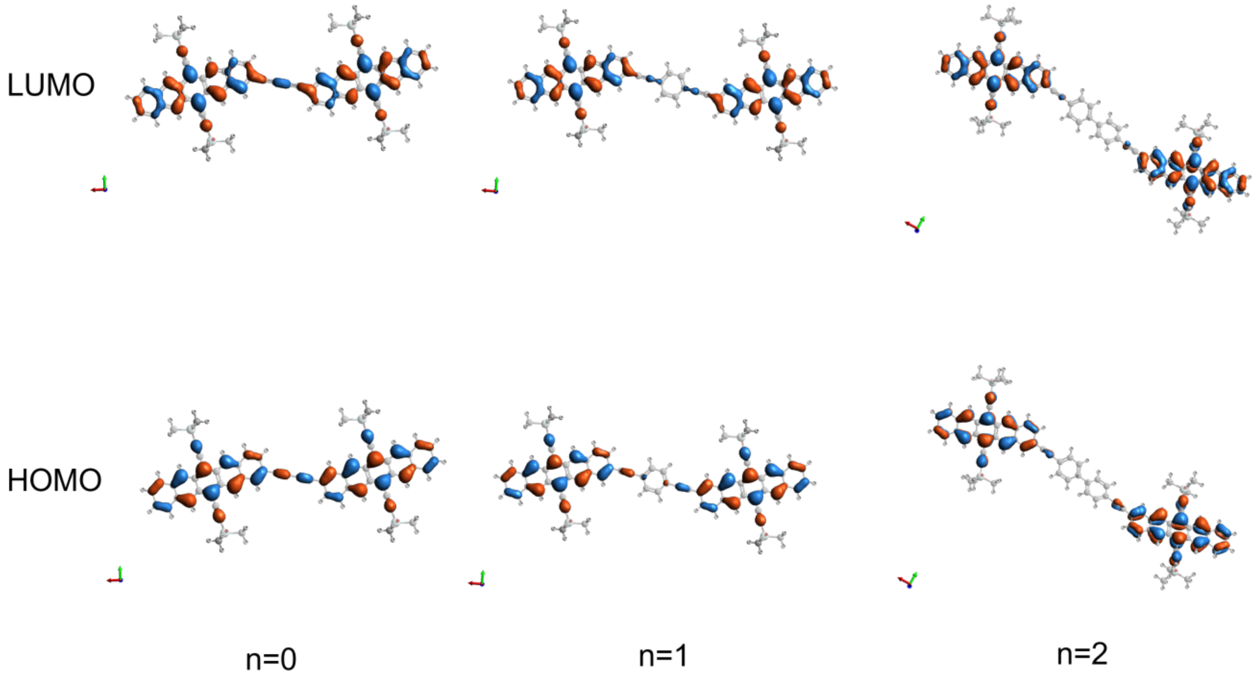

Figure S3.4.5. Frontier molecular orbitals of **2Ac-P2**, **2Ac-P2Ph** and **2Ac-P2BP**.

4.1. Steady State Measurements

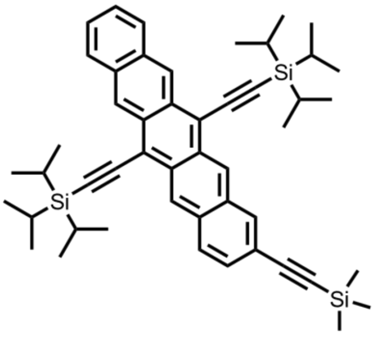

2Ac-PTIPS

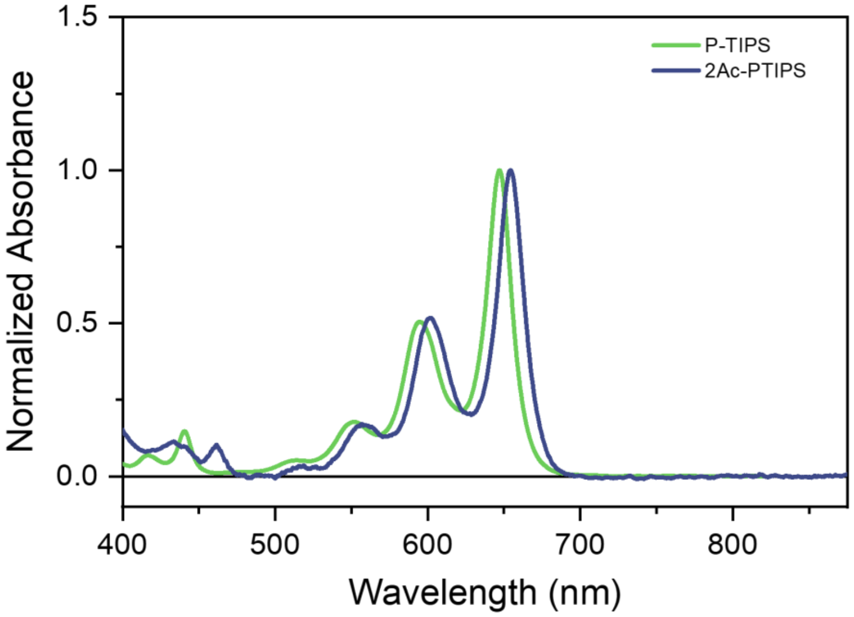

Figure S4.1.1. Structure (left) and steady state absoption (right) of reference monomer **2Ac-PTIPS** in comparison to **P-TIPS** monomer.

Compound		$\lambda_{max,\ 0\text{-}0}$ (nm)[a]
PTIPS		647
2Ac-PTIPS		654
6Ac-P	n=0	749
	n=1	679
	n=2	667
2-P	n=0	662
	n=1	657
	n=2	657
2Ac-P	n=0	662
	n=1	657
	n=2	657

Table S4.1.1. Positions and widths of 0–0 absorption peaks of 6Ac-P, 2-P and 2Ac-P dimers.

*Estimated from the second derivative analysis.

4.2. Transient Absorption Measurements

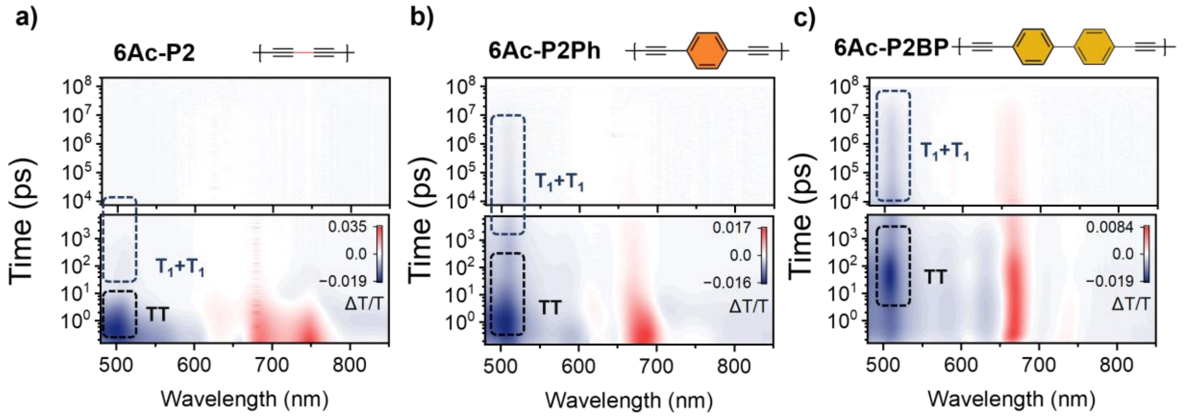

Figure S4.2.1. fs-nsTA contour maps of (a) **6Ac-P2**, (b) **6Ac-P2Ph**, and (c) **6Ac-P2BP** in chlorobenzene after photoexcitation at 0-1/0-2 vibronic absorption bands (fsTA - 680 nm (6Ac-P2), 620 nm (6Ac-P2Ph), 605 nm (6Ac-P2BP); nsTA - 620 nm).

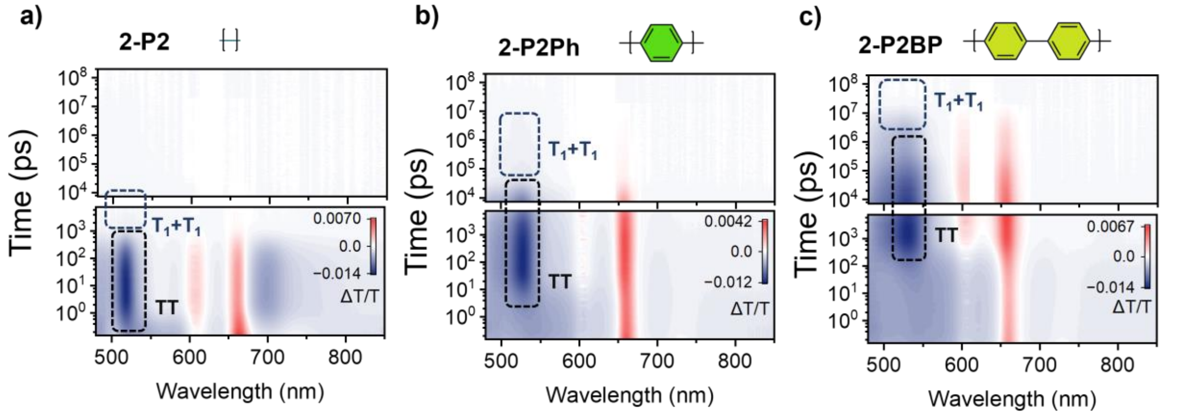

Figure S4.2.2. fs-nsTA contour maps of (a) **2-P2**, (b) **2-P2Ph**, and (c) **2-P2BP** in chlorobenzene after photoexcitation at 0-1 vibronic absorption bands (fsTA - 605 nm, nsTA - 620 nm).

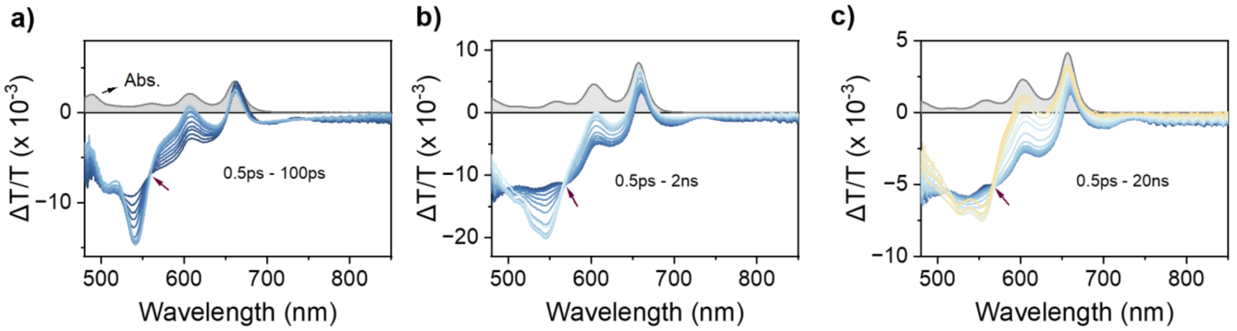

Figure S4.2.3. Isosbestic points in fsTA of (a) **2Ac-P2**, (b) **2Ac-P2Ph**, and (c) **2Ac-P2BP** indicated with maroon arrow. This implies direct population transfer between S_1 and ^{1}(TT) states during the mentioned time windows.

**6Ac-P**		
**6Ac-P2**	**6Ac-P2Ph**	**6Ac-P2BP**
• **Planar (no SF based on 750, 780 nm pump results):** *1.32 ps (± 0.046) (S_1 lifetime or ^{1}(TT) recombination)*<br>• **Twisted (no SF, reflected as S_1 spectra's decay):** *650-800 ps / 5.5 ns (± 0.097)*<br>• **Twisted (SF):** *IRF limited ^{1}(TT) formation/ 4ps (± 0.086) ^{1}(TT) recombination) / 77 ns (± 1.7) (^{m}(TT) recombination / spin evolution) / 8 μs (± 0.28) (free triplet)*	**Planar (SF):**<br>• *0.3 ps (± 0.1) rise, ^{1}(TT) formation*<br>• *3.1 ps (± 0.012) decay,(^{1}(TT) recombination or formation not clear)*<br>• *16.6 ps (± 0.044) (^{1}(TT) recombination)*<br>• *4-8 ns (S_1 decay)*<br>• *95-120 ns (^{m}(TT) recombination / spin evolution)*<br>• *23 μs (± 0.23) (free triplet)*	**Twisted (SF):**<br>• *15 ps rise (± 1.1) (^{1}(TT) formation)*<br>• *239 ps ^{1}(TT) (± 10.2) recombination*<br>• *13.4 ns (S_1 decay)*<br>• *188 ns (^{m}(TT) recombination / spin evolution)*<br>• *26 μs (± 0.22) (free triplet)*
**2-P**		
**2-P2 (BP0)**	**2-P2Ph (BP1)**	**2-P2BP (BP2)**
**Twisted (SF):**<br>• *0.93 ps (± 0.05) (^{1}(TT) formation)*<br>• *420-460 ps (^{1}(TT) recombination)*<br>• *15.0 ns (± 0.3) (S_1 decay)*<br>• *19 μs (± 2.3) (free triplet or triplet)*	**Twisted (SF):**<br>• *13 ps (± 0.3) (^{1}(TT) formation)*<br>• *14.3 ns (± 0.9) (^{1}(TT) recombination)*<br>• *228 ns (± 5.1) (^{m}(TT) recombination / spin evolution)*<br>• *24.7 μs (± 1.7) (free triplet or triplet)*	**Twisted (SF):**<br>• *323 ps (± 5.8) (^{1}(TT) formation)*<br>• *43 ns (± 9.4) (^{1}(TT) recombination)*<br>• *262 ns (± 8.5) (^{m}(TT) recombination / spin evolution)*<br>• *19.9 μs (± 0.7) (free triplet or triplet)*
**2Ac-P**		
**2Ac-P2**	**2Ac-P2Ph**	**2Ac-P2BP**
**Planar (SF):**<br>• *2.7 ps (± 0.25) (^{1}(TT) formation)*<br>• *20 ps (± 1.0) (^{1}(TT) formation)*<br>• *860 ps (± 10.2) (^{1}(TT) recombination)*<br>• *15.4 ns (± 0.26) (S_1 decay)*<br>• *26.5 μs (± 4.7) (free triplet or triplet)*	**Planar (SF):**<br>• *84 ps (± 3.6) (^{1}(TT) formation)*<br>• *18.2 ns (± 1.5) (^{1}(TT) recombination)*<br>• *122.5 ns (± 2.3) (^{m}(TT) recombination / spin evolution)*<br>• *28.0 μs (± 3.6) (free triplet or triplet)*	**Twisted (SF):**<br>• *2.0 ns (± 0.04) (^{1}(TT) formation)*<br>• *48.8 ns (± 3.0) (^{1}(TT) recombination)*<br>• *27.8 μs (± 1.9) (free triplet or triplet)*

Table S4.2.1. Multiexponential fitting results. S_1 decay refers to decay of the singlet excited state either via iSF or directly back to ground state. ^{m}TT recombination or spin evolution encompasses the full range of spin relaxation processes within the TT manifold since these states are indistinguishable in our optical measurement.

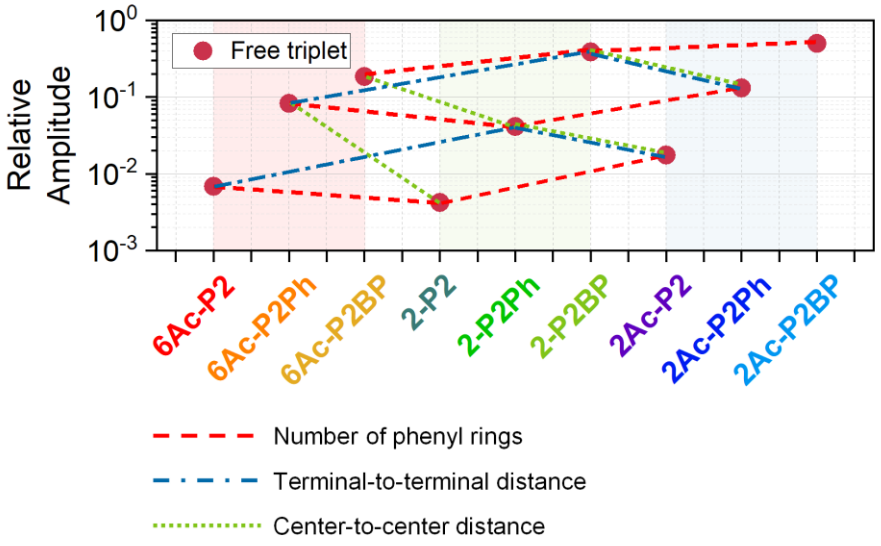

Figure S4.2.4. Comparison of relative amplitudes of free triplet decay in the kinetics of the three dimer classes based on the various interpretations of pentacene-to-pentacene interchromophore distances. Dimers having similar interpentacene distances are connected with dashed lines (number of phenyl rings: red, terminal-to-terminal: blue and center-to-center: green. Trends depend strongly on the interpretation highlighting the limitations of a simple chromophore-linker-chromophore based analysis.

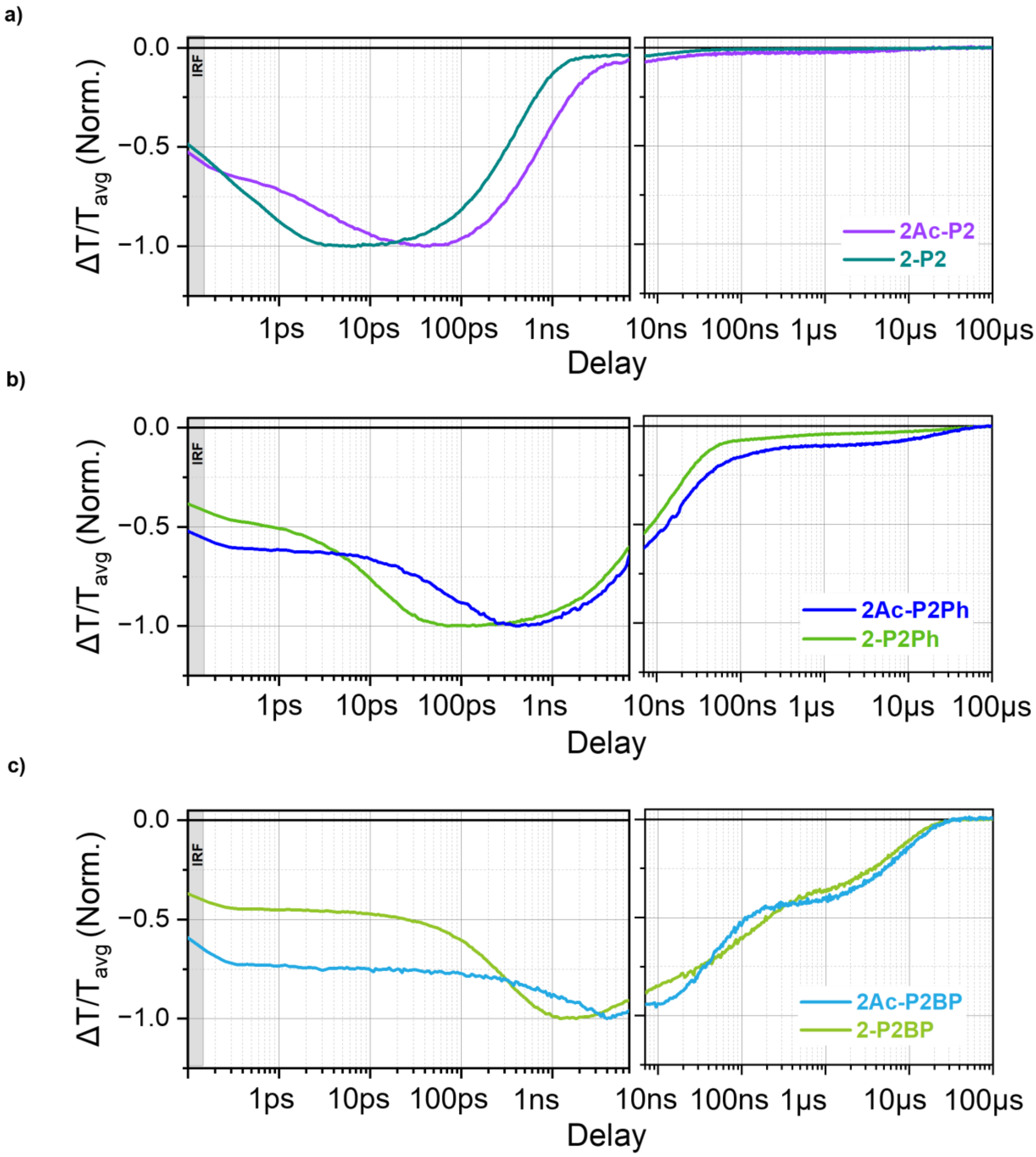

Figure S4.2.5. Comparison of normalized [1](TT) dynamics between 2Ac-P and 2-P dimers with a) n=0 b) n=1 and c) n=2 oligophenylene linkage after photoexcitation at respective 0-1 vibronic absorption bands.

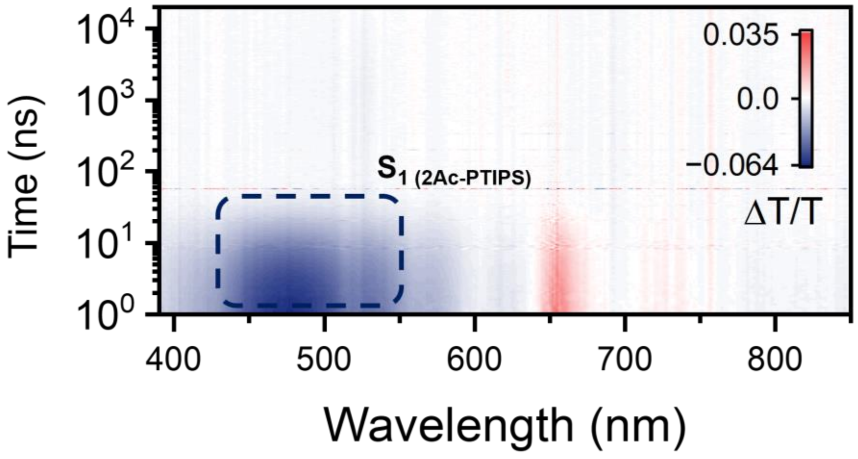

Figure S4.2.6. nsTA contour map of **2Ac-PTIPS** in chlorobenzene after photoexcitation at 0-1 vibronic absorption bands (620 nm).

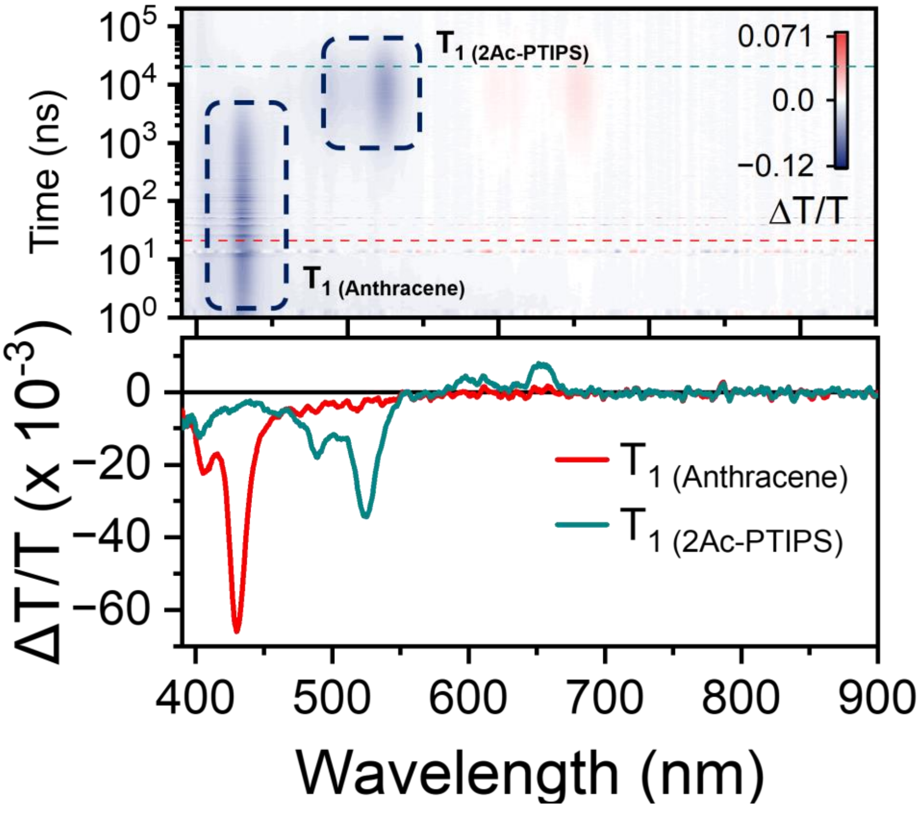

Figure S4.2.7. nsTA results demonstrating triplet sensitisation of **2Ac-PTIPS** using anthracene as triplet sensitiser in chlorobenzene after photoexcitation at 360 nm. Top and bottom panels show contour maps and TA spectra at selected delay times, respectively.

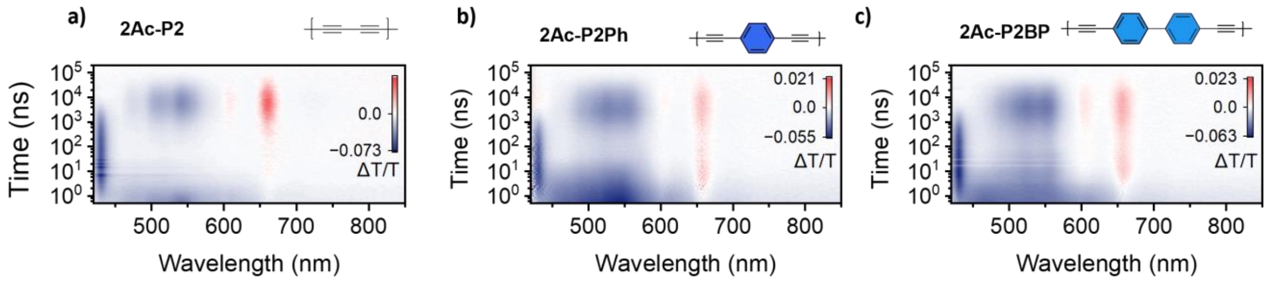

Figure S4.2.8. nsTA results demonstrating triplet sensitisation of **a) 2Ac-P2, b) 2Ac-P2Ph** and **c) 2Ac-P2BP** using anthracene as triplet sensitiser in chlorobenzene after photoexcitation at 360 nm.

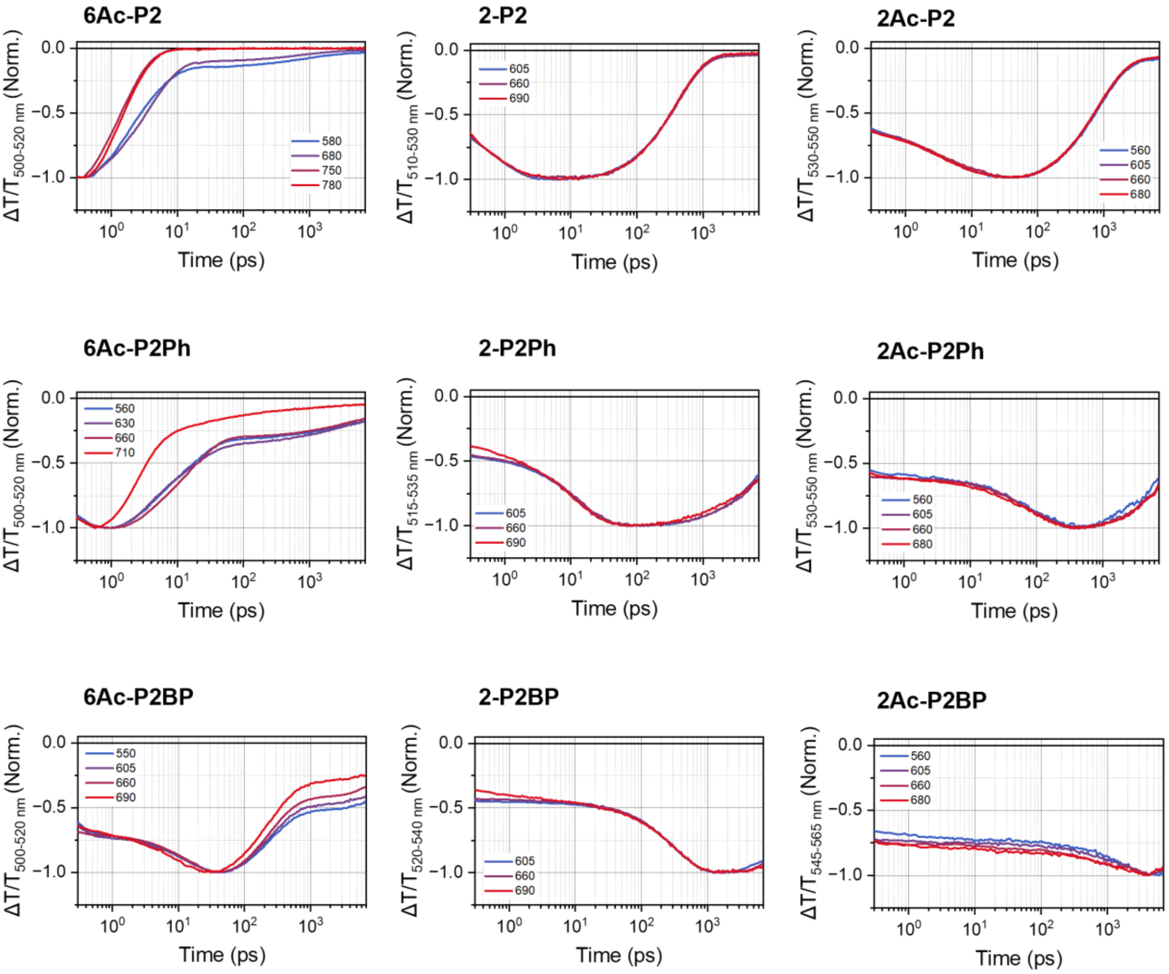

Figure S4.2.9. Heterogeneous iSF: Comparison of normalized kinetics in TT PIA regions among **6Ac-P** (left), **2-P** (middle) and **2Ac-P** (right) dimers at different excitation wavelengths. **6Ac-P** dimers exhibit strong excitation dependent iSF dynamics while both **2-P** and **2Ac-P** dimers exhibit iSF largely insensitive to excitation wavelength.

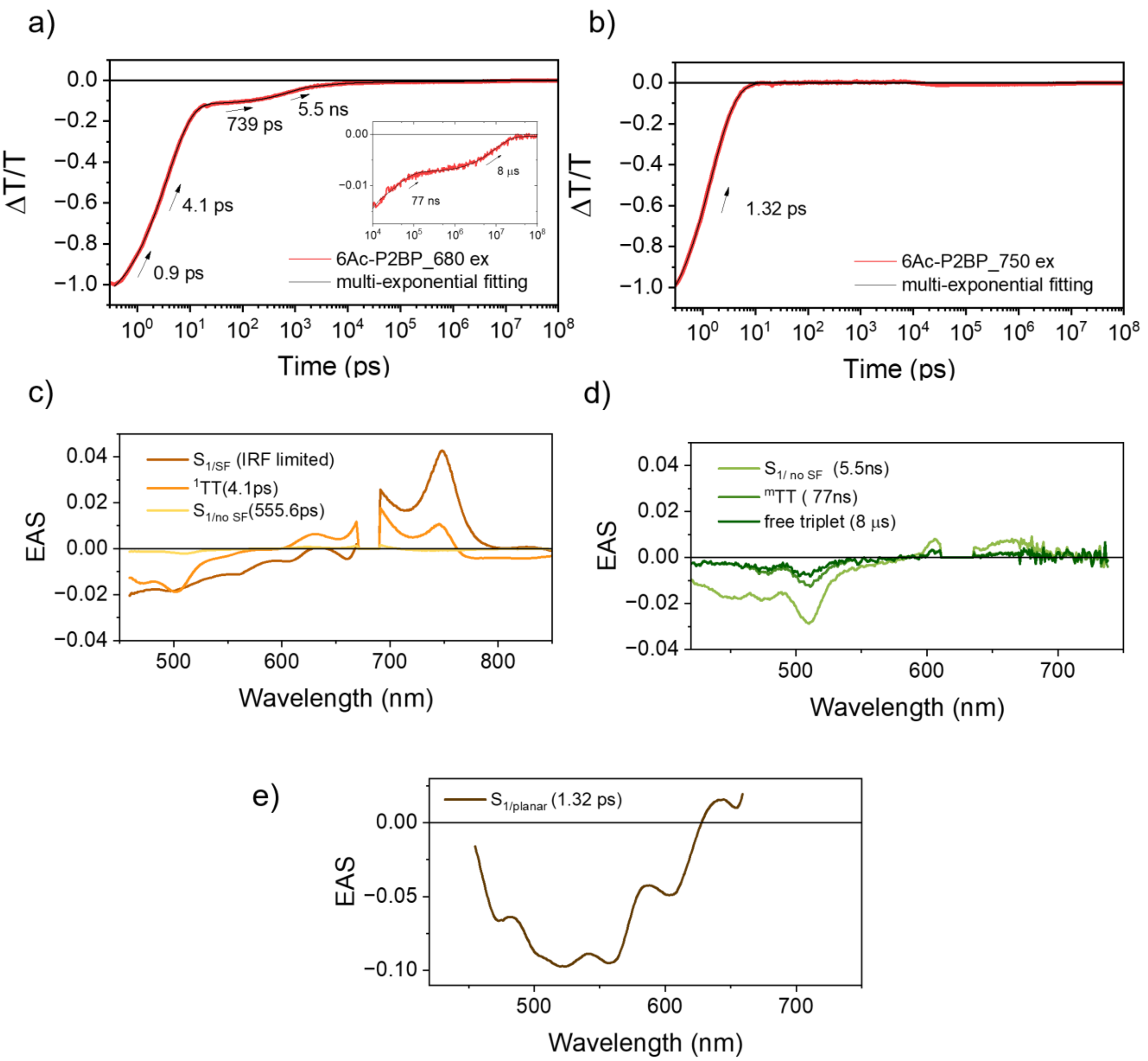

Figure S4.2.10. Kinetic analysis of (a) ^{1}TT PIA region ($\Delta T/T_{avg}$) with 680nm excitation in **6Ac-P2** and (b) S_1 PIA region with 750nm excitation using multiexponential fit. (c) fsTA and (d) nsTA global analysis showing evolution associated spectra (EAS) with 680nm excitation. (e) EAS with 750nm excitation. For more detailed analysis of 6Ac-P2 dimers refer to J. Phys. Chem. Lett. 2022, 13, 5094−5100.

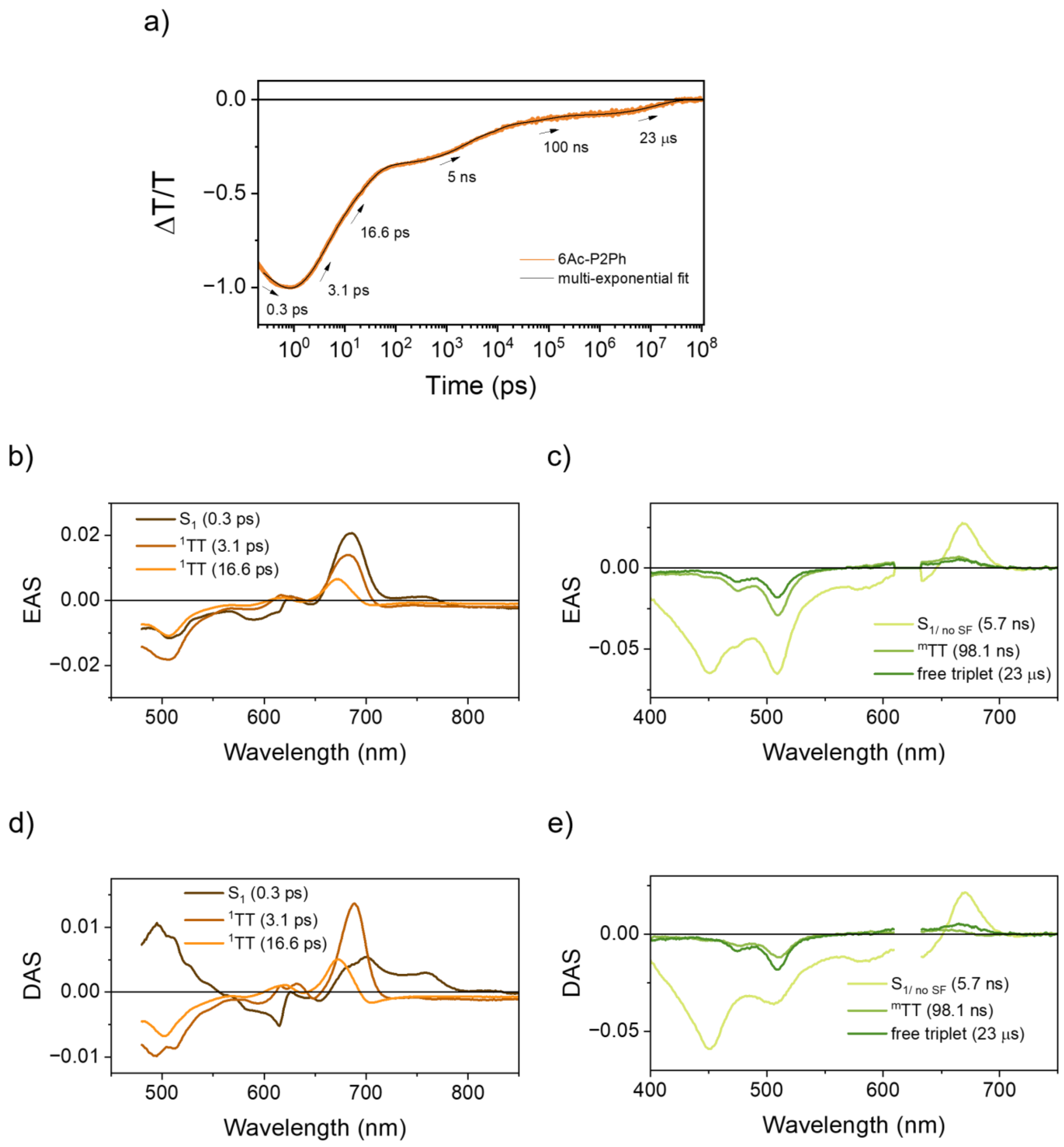

Figure S4.2.11. (a) Kinetic analysis of ^{1}TT PIA region ($\Delta T/T_{avg}$) in **6Ac-P2Ph** using multiexponential fit. (b) fsTA and (c) nsTA EAS from global analysis. (d) fsTA and (e) nsTA decay associated spectra (DAS) from global analysis. $S_{1/no\ SF}$ corresponds to SI subpopulation that does not undergo singlet fission. For more detailed analysis of 6Ac-P2Ph dimers refer to J. Am. Chem. Soc. 2023, 145, 20883−20896.

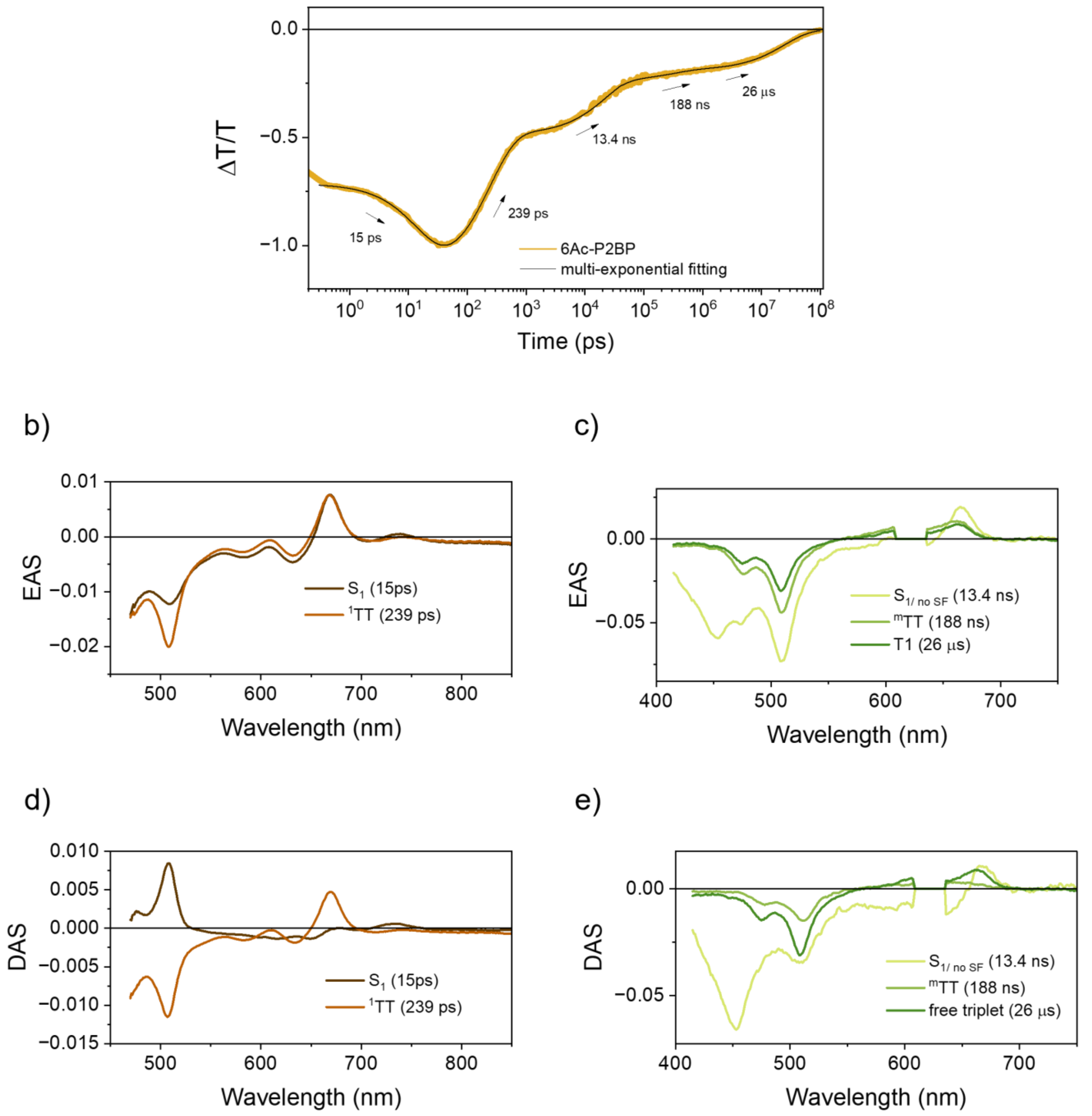

Figure S4.2.12. (a) Kinetic analysis of ^{1}TT PIA region ($\Delta T/T_{avg}$) in **6Ac-P2BP** using multiexponential fit. (b) fsTA and (c) nsTA EAS from global analysis. (d) fsTA and (e) nsTA DAS from global analysis. $S_{1/no\ SF}$ corresponds to SI subpopulation that does not undergo singlet fission. For more detailed analysis of 6Ac-P2BP dimers refer to J. Am. Chem. Soc. 2023, 145, 20883−20896.

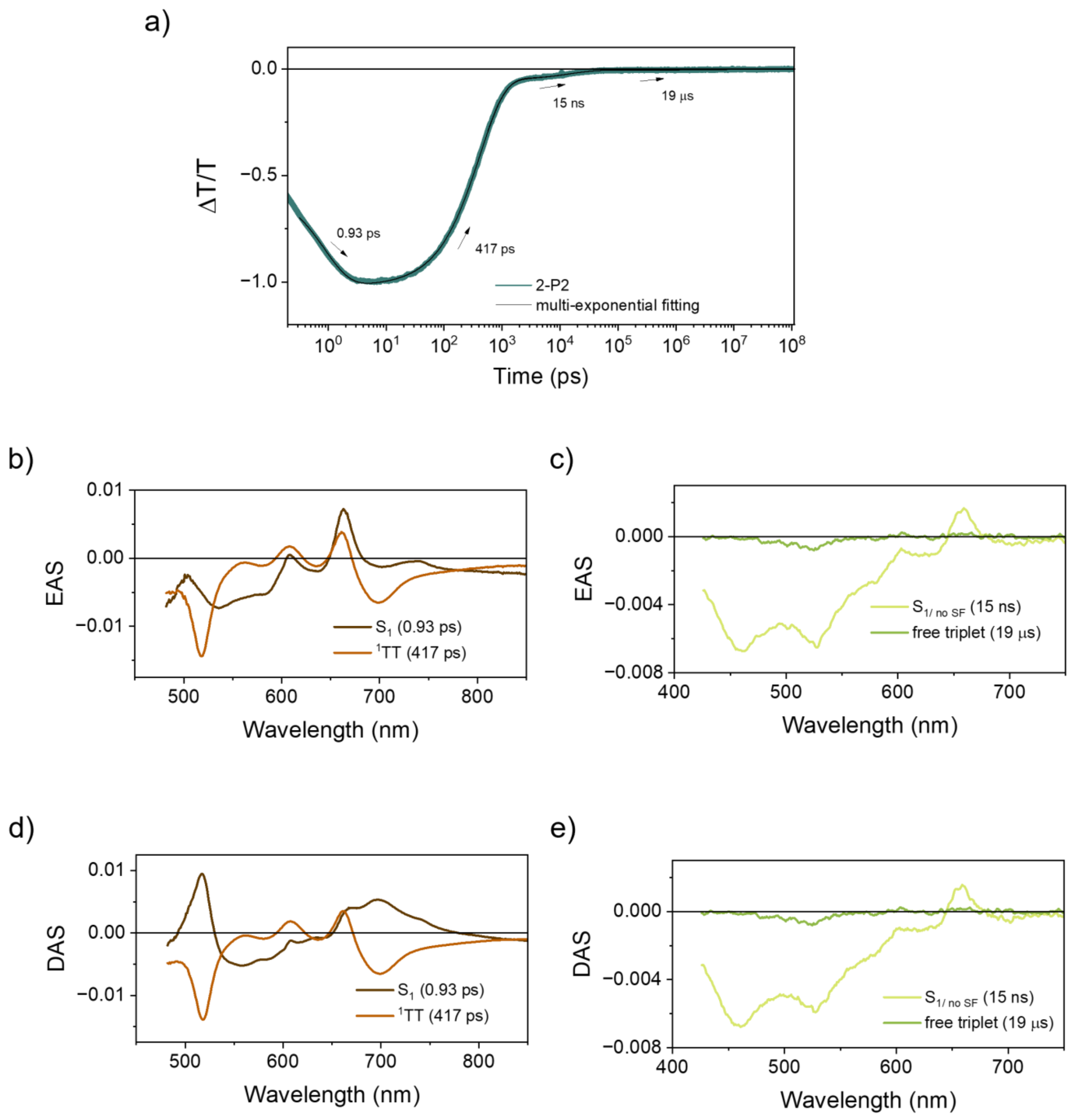

Figure S4.2.13. (a) Kinetic analysis of ^{1}TT PIA region ($\Delta T/T_{avg}$) in **2-P2** using multiexponential fit. (b) fsTA and (c) nsTA EAS from global analysis. (d) fsTA and (e) nsTA DAS from global analysis. $S_{1/no\ SF}$ corresponds to SI subpopulation that does not undergo singlet fission.

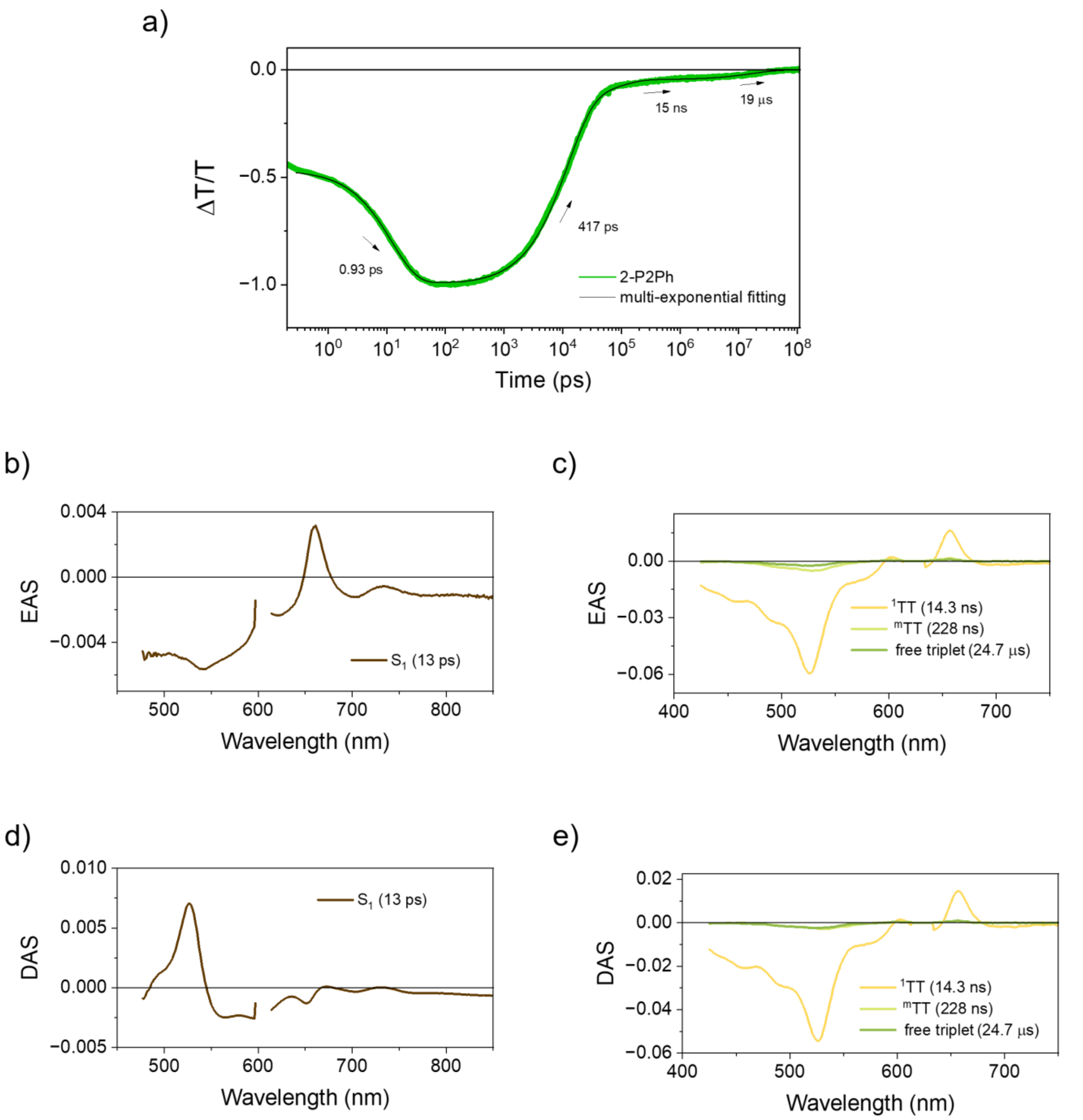

Figure S4.2.14. (a) Kinetic analysis of ^{1}TT PIA region ($\Delta T/T_{avg}$) in **2-P2Ph** using multiexponential fit. (b) fsTA and (c) nsTA EAS from global analysis. (d) fsTA and (e) nsTA DAS from global analysis.

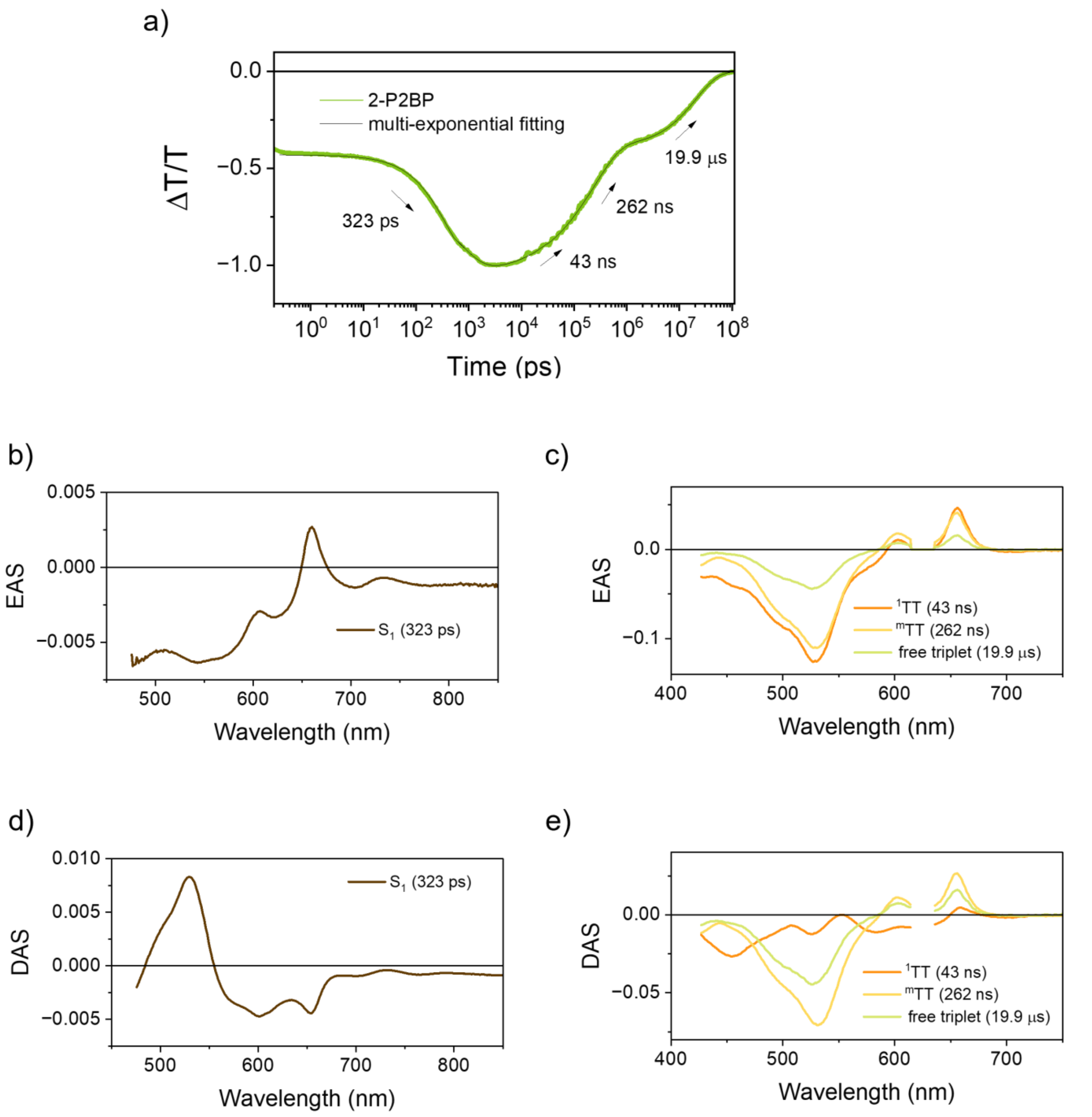

Figure S4.2.15. (a) Kinetic analysis of ^{1}TT PIA region ($\Delta T/T_{avg}$) in **2-P2BP** using multiexponential fit. (b) fsTA and (c) nsTA EAS from global analysis. (d) fsTA and (e) nsTA DAS from global analysis.

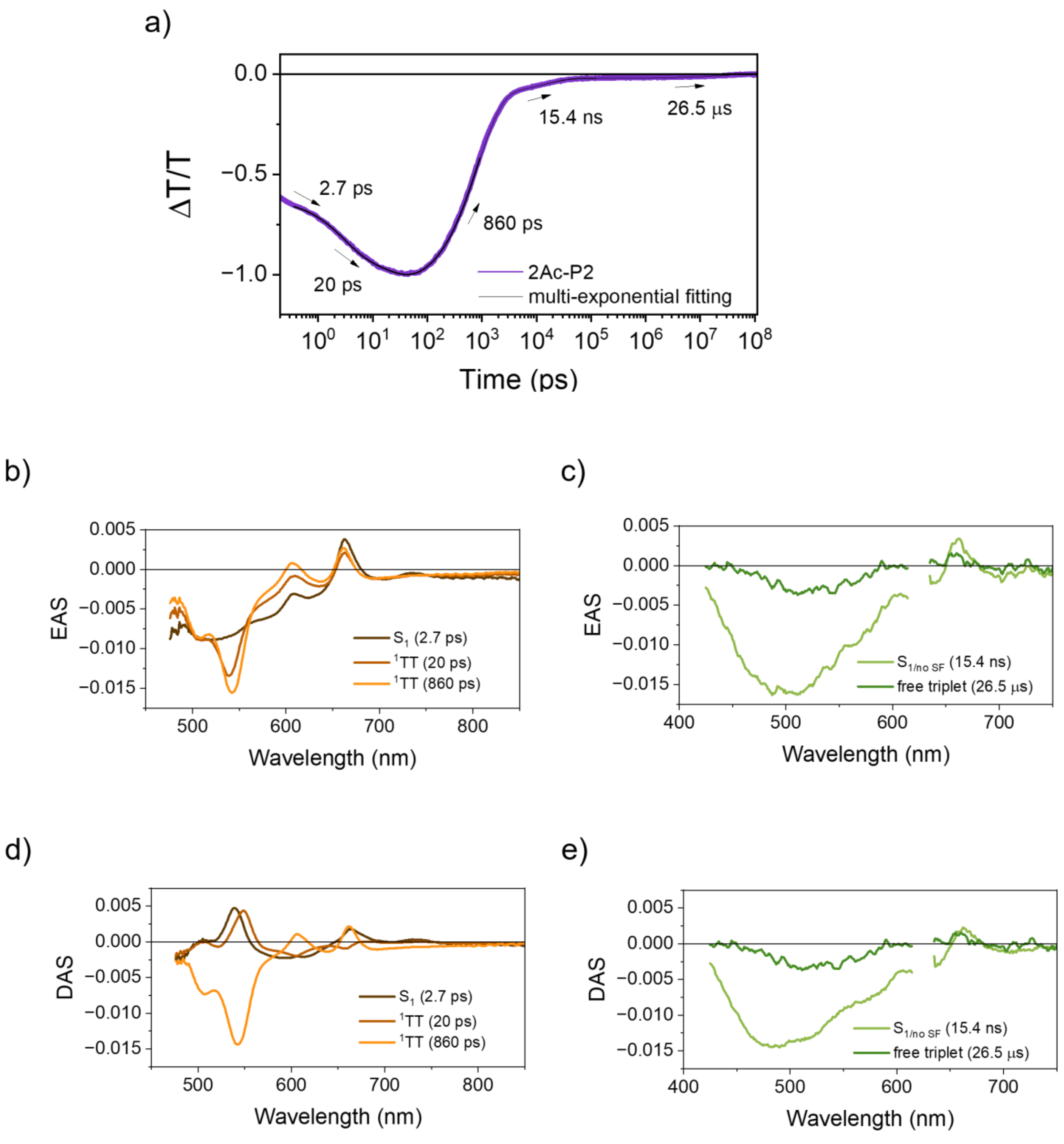

Figure S4.2.16. (a) Kinetic analysis of ^{1}TT PIA region ($\Delta T/T_{avg}$) in **2Ac-P2** using multiexponential fit. (b) fsTA and (c) nsTA EAS from global analysis. (d) fsTA and (e) nsTA DAS from global analysis. $S_{1/no\ SF}$ corresponds to SI subpopulation that does not undergo singlet fission.

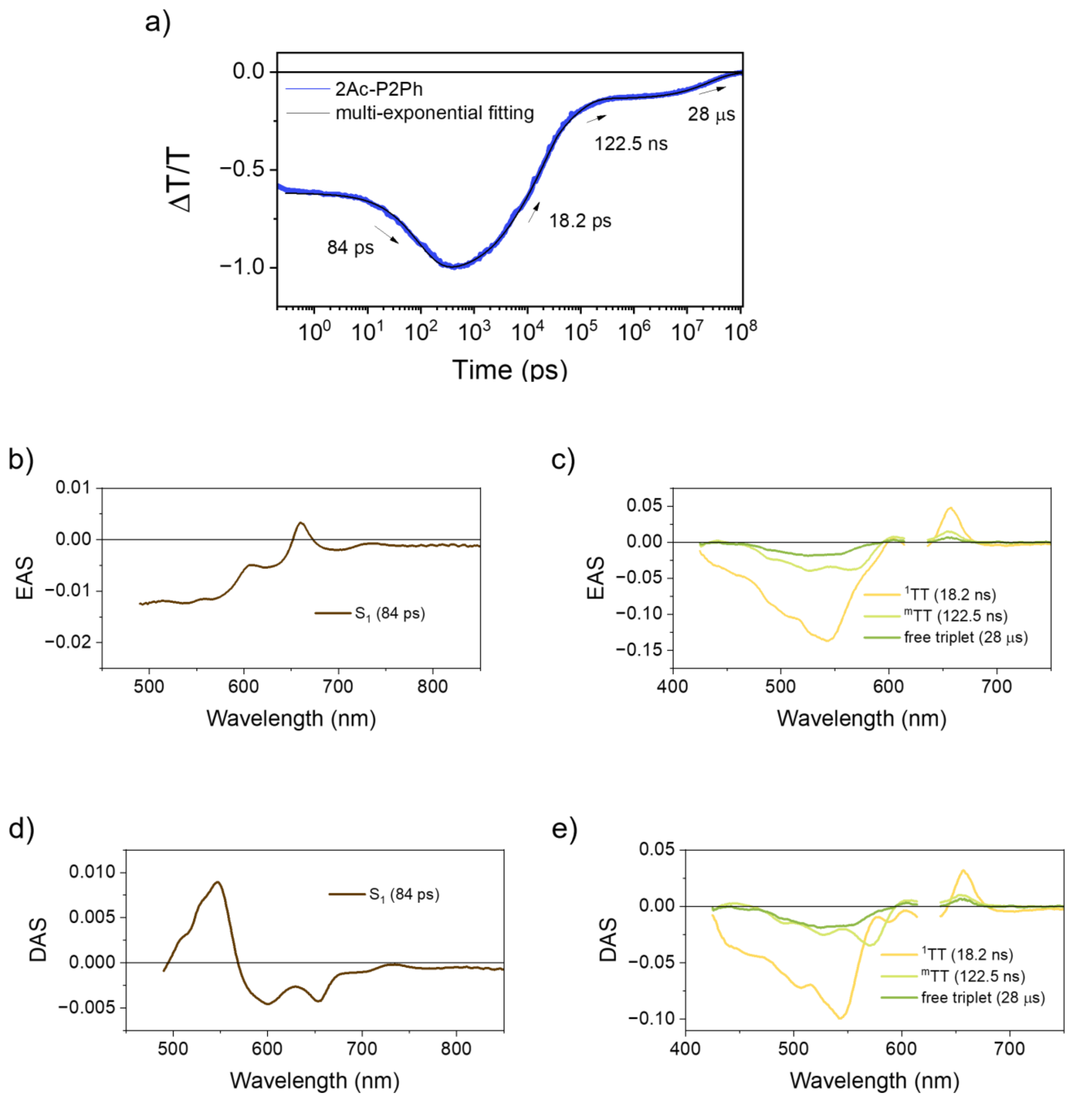

Figure S4.2.17. (a) Kinetic analysis of ^{1}TT PIA region ($\Delta T/T_{avg}$) in **2Ac-P2Ph** using multiexponential fit. (b) fsTA and (c) nsTA EAS from global analysis. (d) fsTA and (e) nsTA DAS from global analysis.

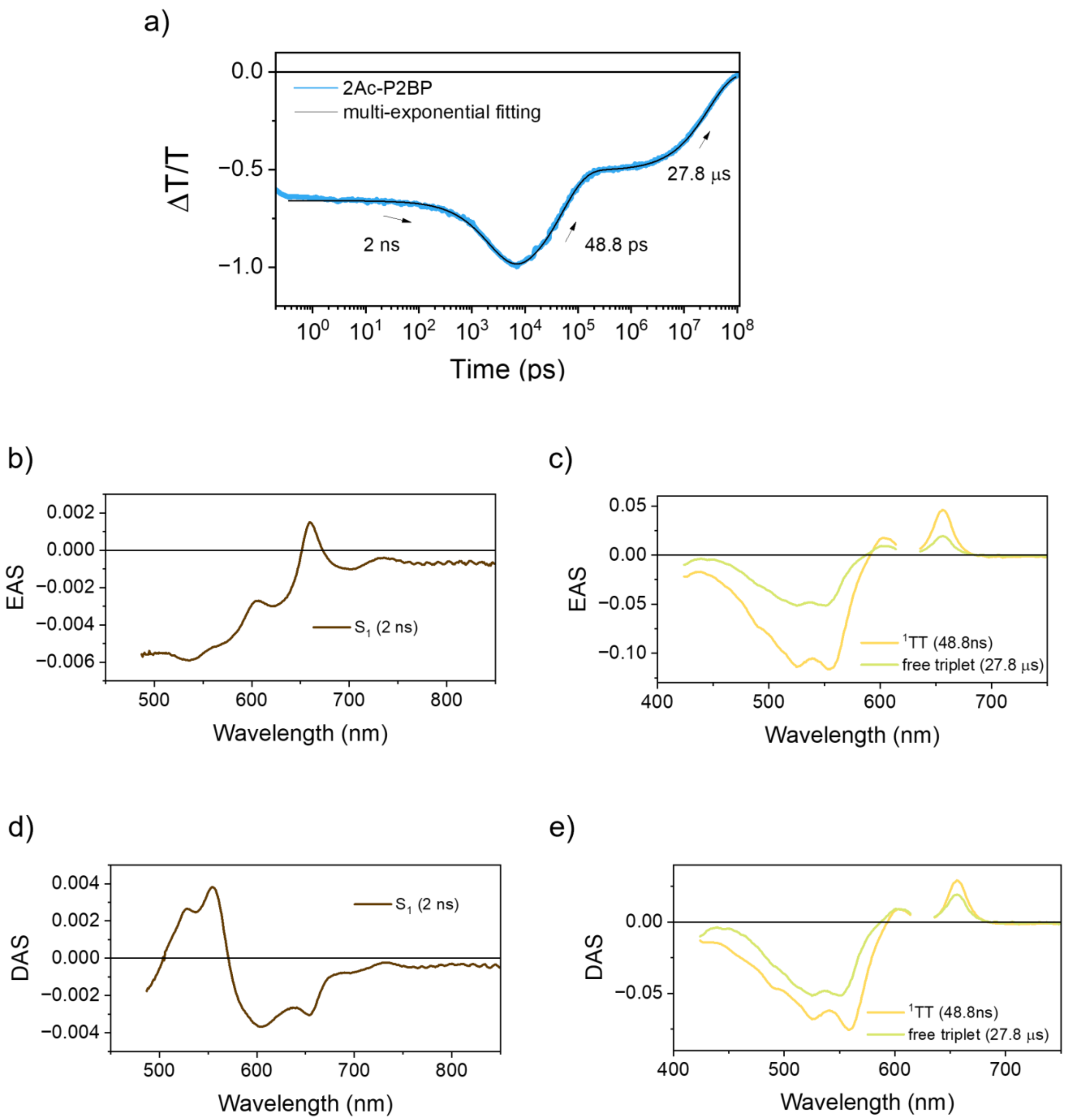

Figure S4.2.18. (a) Kinetic analysis of ^{1}TT PIA region ($\Delta T/T_{avg}$) in **2Ac-P2BP** using multiexponential fit. (b) fsTA and (c) nsTA EAS from global analysis. (d) fsTA and (e) nsTA DAS from global analysis.

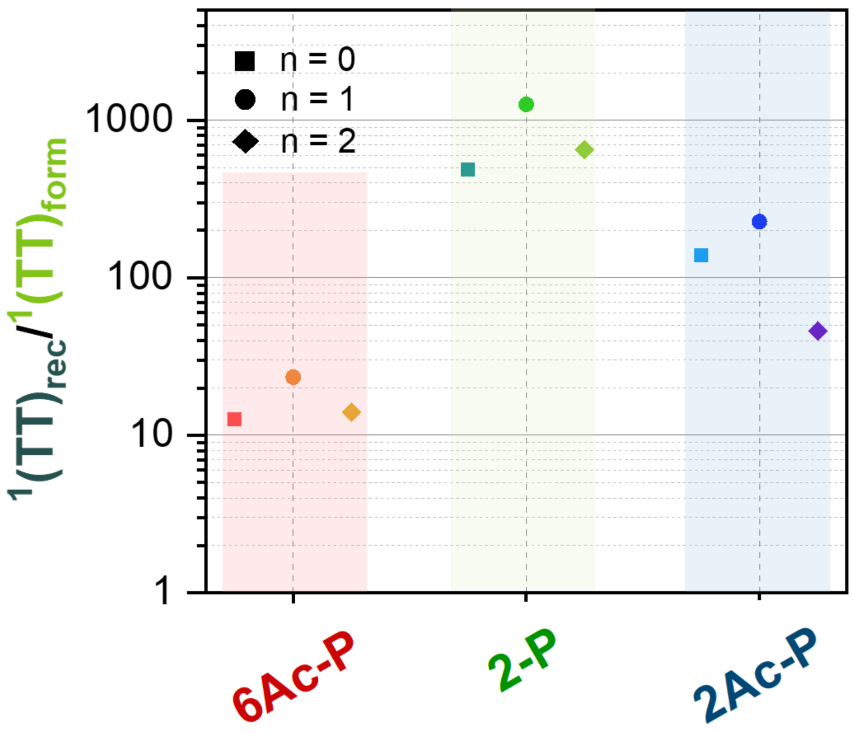

Figure S4.2.19. Ratio of $^1TT_{rec}$ to $^1TT_{form}$ in the different dimer classes shows both the **6Ac-P** and **2Ac-P** dimers have enhanced $^1TT_{rec}$ relative to its $^1TT_{form}$ as compared to the **2-P** dimers. For the ratio we have used the primary $^1TT_{rec}$ and $^1TT_{form}$ lifetimes.

5. ^{1}H, ^{13}C NMR Spectra and MALDI-TOF MS:

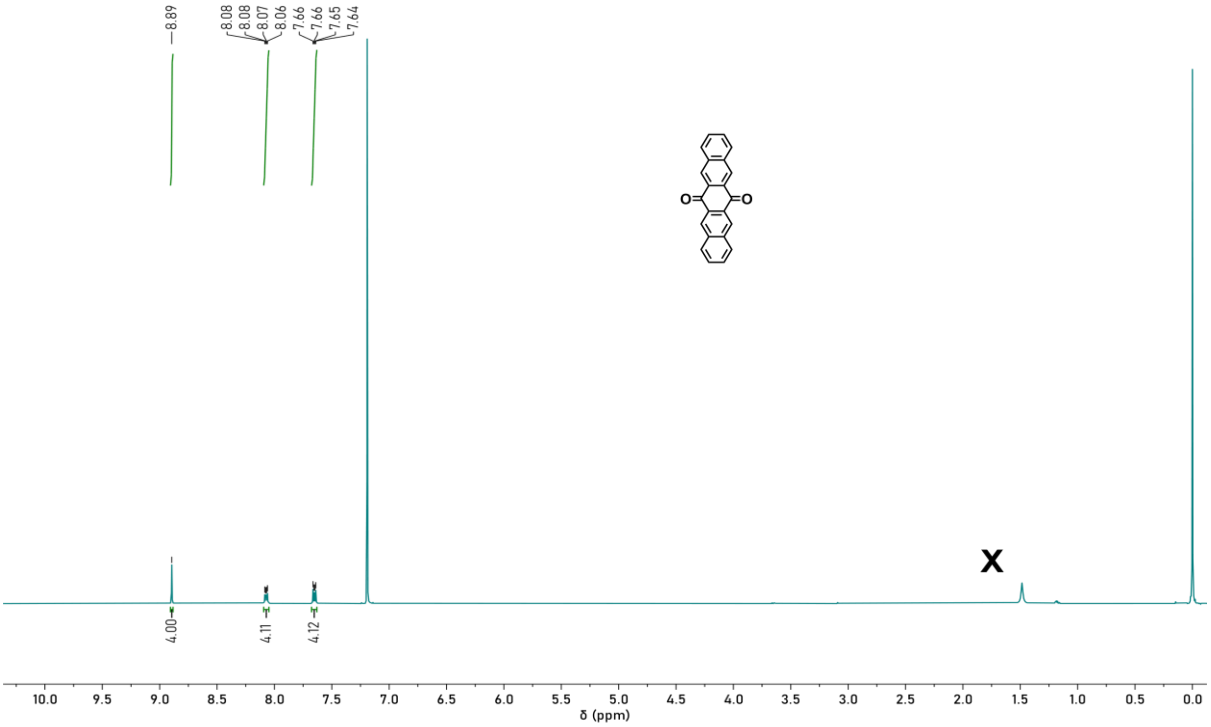

Figure S5.1. *^{1}H NMR spectrum of 8a in $CDCl_3$ [x = H_2O]*

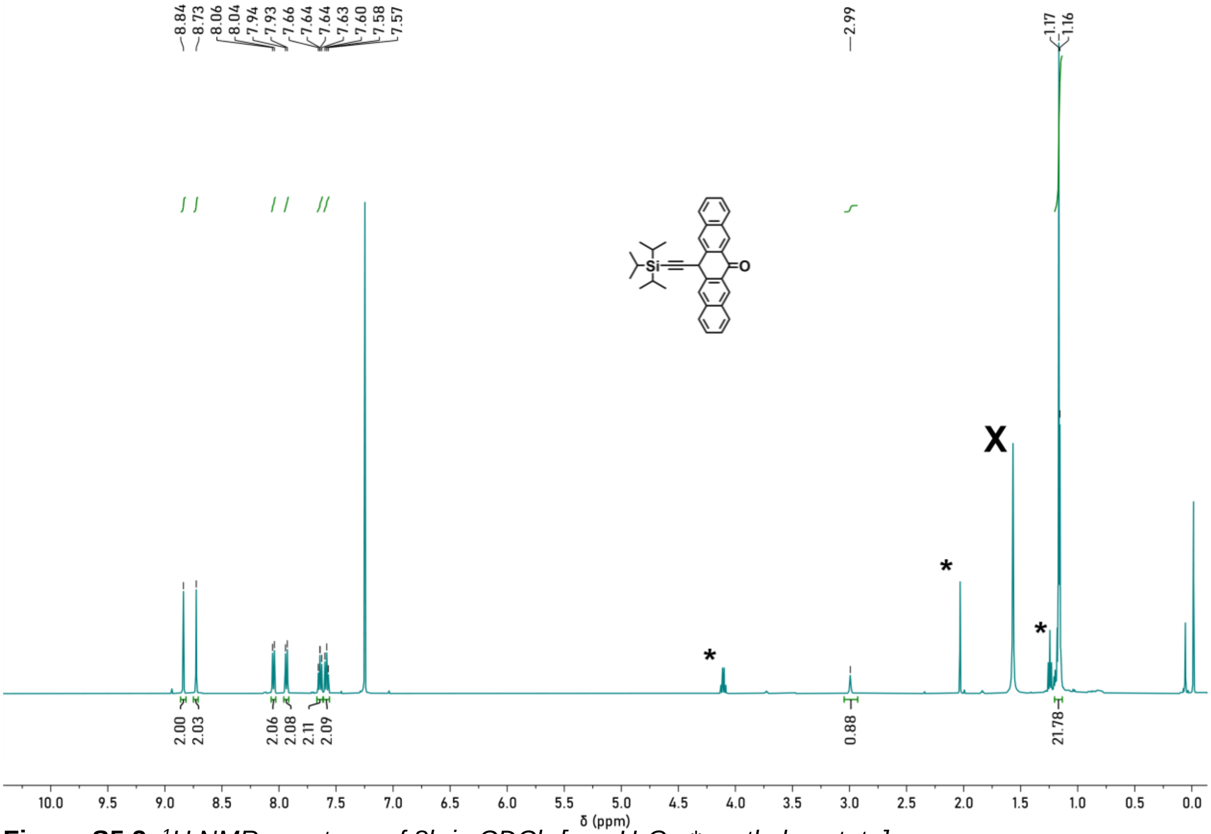

Figure S5.2. *^{1}H NMR spectrum of 8b in $CDCl_3$ [x = H_2O ; * = ethyl acetate]*

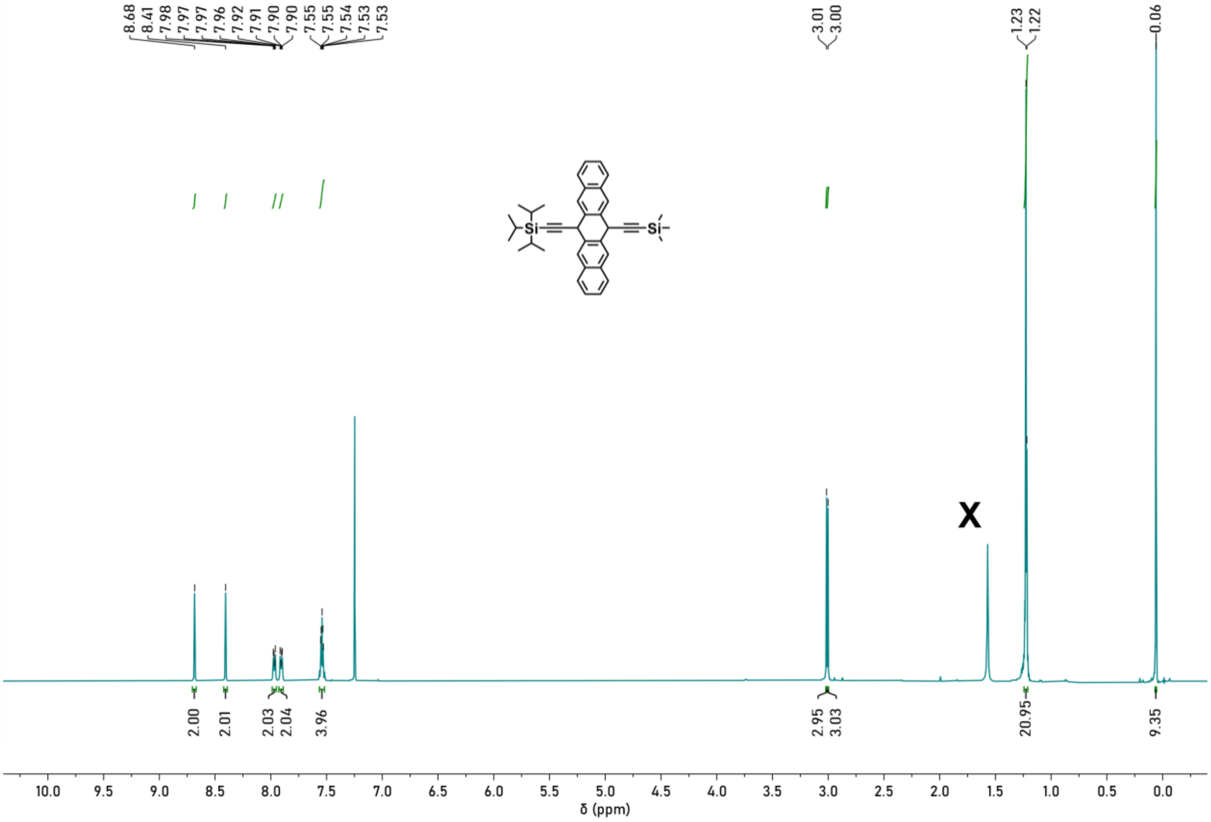

Figure S5.3. *^{1}H NMR spectrum of 8c in $CDCl_3$ [x = H_2O]*

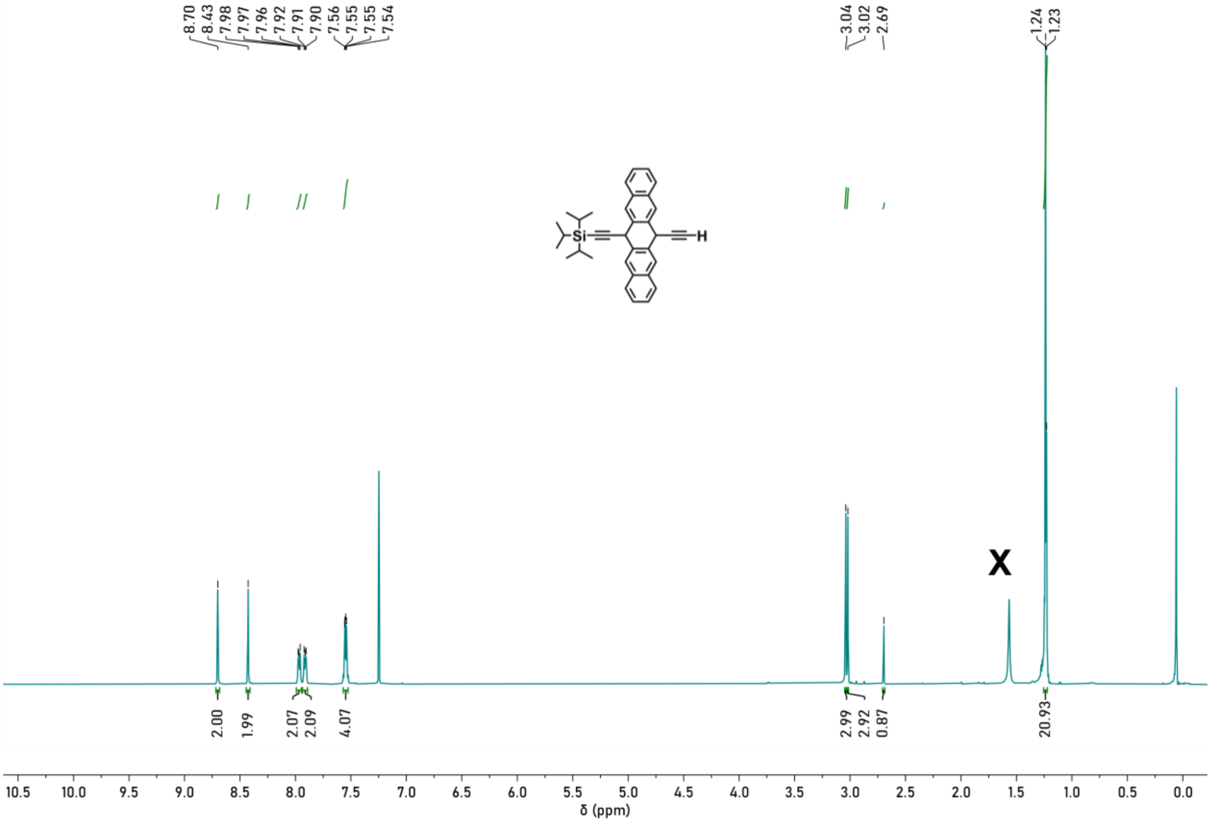

Figure S5.4. *^{1}H NMR spectrum of 6 in $CDCl_3$ [x = H_2O]*

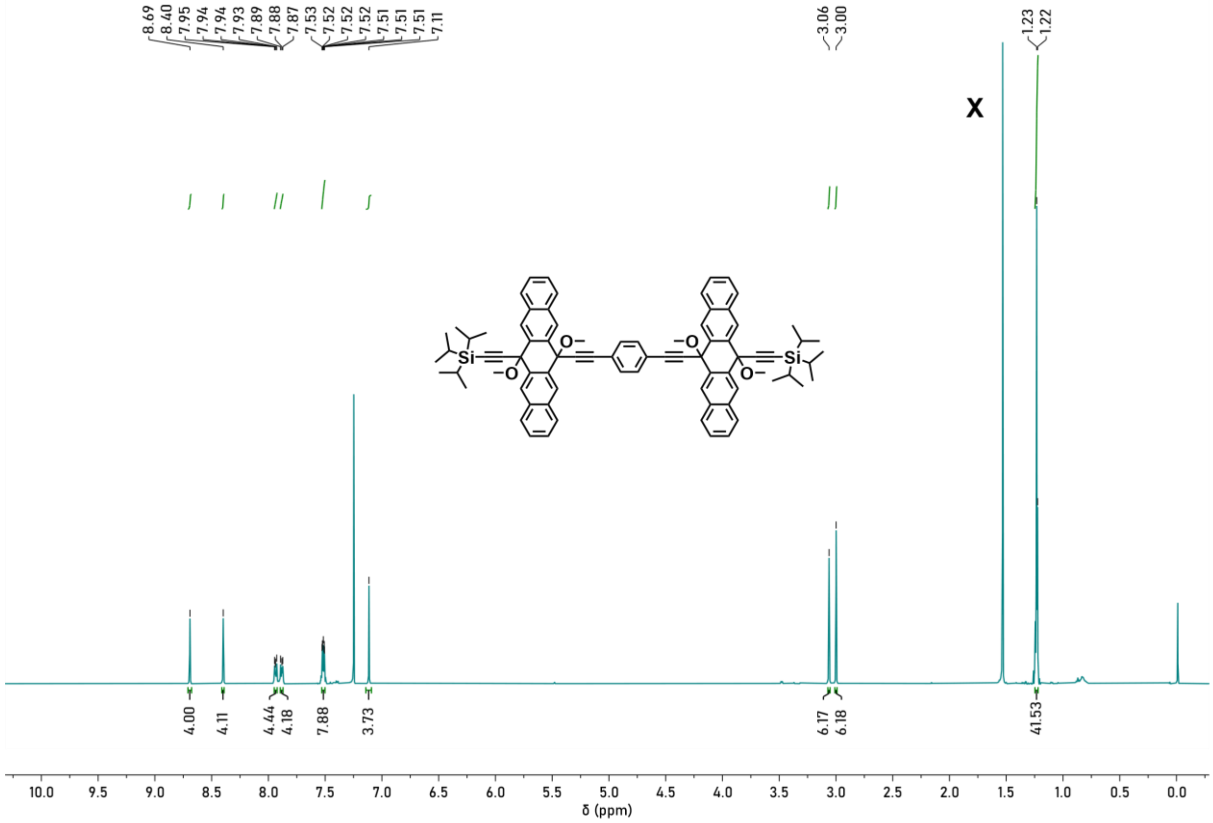

Figure S5.5. *^{1}H NMR spectrum of 7a(n=1) in $CDCl_3$ [x = H_2O]*

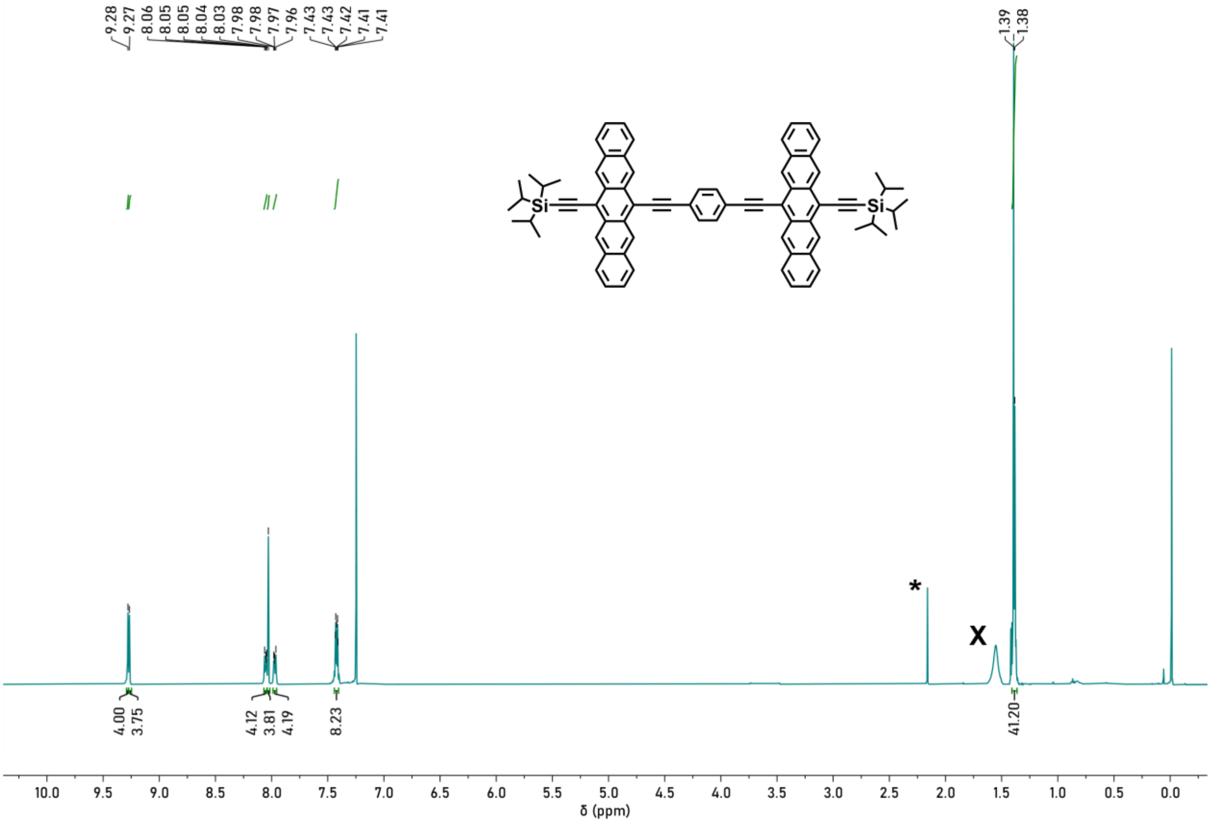

Figure S5.6. *^{1}H NMR spectrum of **6Ac-P2Ph** in $CDCl_3$ [x = H_2O ; * = acetone]*

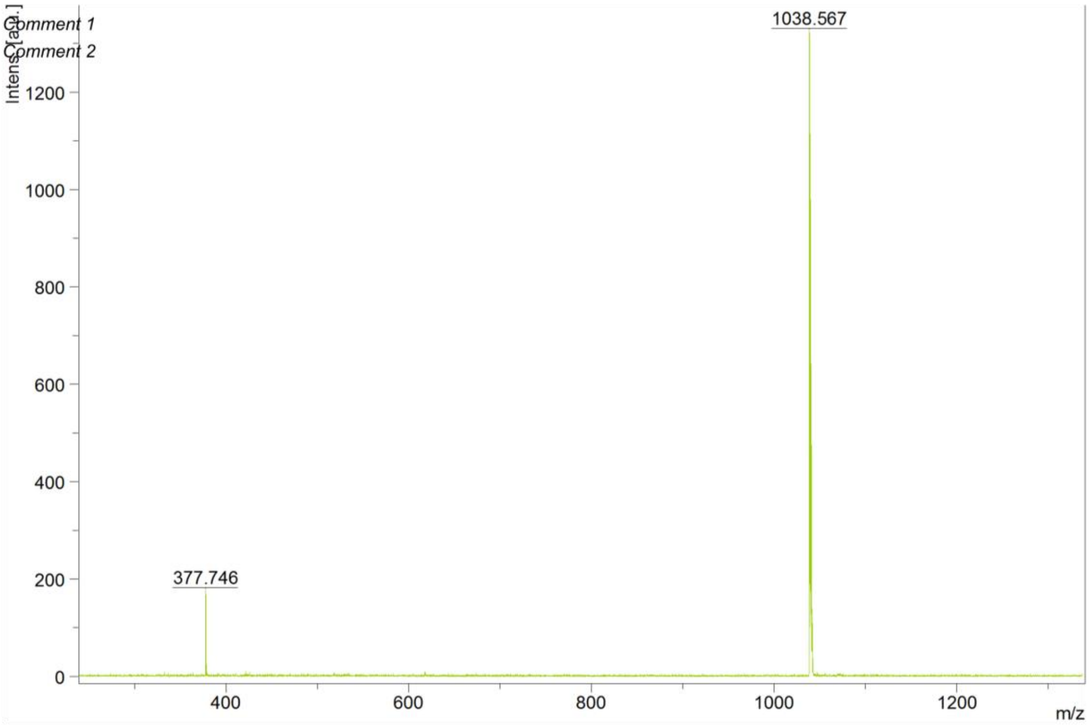

Acquisition Parameter

Date of acquisition	2020-02-05T16:35:19.208+05:30
Acquisition method name	D:\Methods\flexControlMethods\LP_700-2000_Da.par
Aquisition operation mode	Linear
Voltage polarity	POS
Number of shots	500
Name of spectrum used for calibration	
Calibration reference list used	PeptideCalibStandard mono

Instrument Info

User	JNCASR
Instrument	ATS-00704
Instrument type	autoflex

Bruker Daltonics flexAnalysis printed: 2/26/2020 4:55:31 PM

Figure S5.7. *MALDI-MS of* ***6Ac-P2Ph***

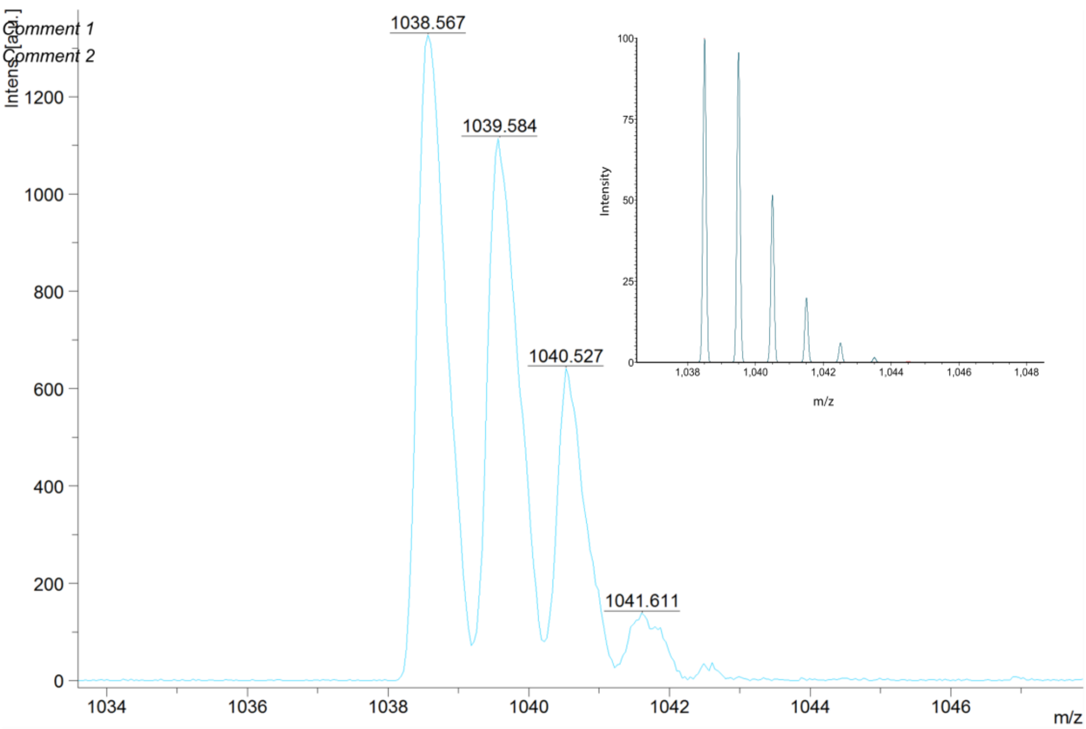

m/z	S/N	Quality Fac.	Res.	Intens.	Area
1038.567				1327	
1039.584	160		720	1115	1637
1040.527				642	
1041.611				138	

Acquisition Parameter

Date of acquisition	2020-02-05T16:35:19.208+05:30
Acquisition method name	D:\Methods\flexControlMethods\LP_700-2000_Da.par
Aquisition operation mode	Linear
Voltage polarity	POS
Number of shots	500
Name of spectrum used for calibration	
Calibration reference list used	PeptideCalibStandard mono

Instrument Info

User	JNCASR
Instrument	ATS-00704
Instrument type	autoflex

Bruker Daltonics flexAnalysis printed: 7/8/2022 4:48:22 PM

Figure S5.8. *MALDI-MS isotopic distribution of* ***6Ac-P2Ph****, (M^+). Simulated isotopic distribution of 3f (inset).*

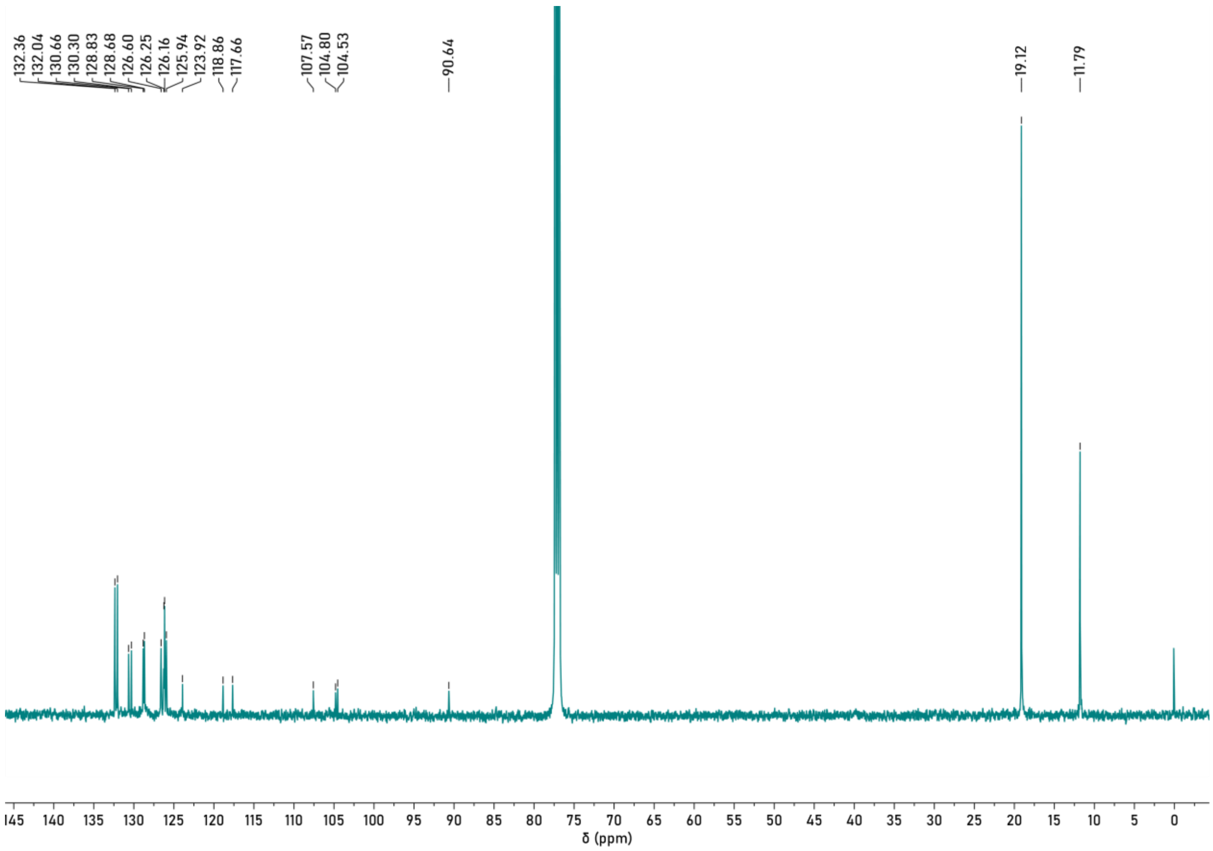

Figure S5.9. *^{13}C NMR spectrum of* ***6Ac-P2Ph*** *in $CDCl_3$*

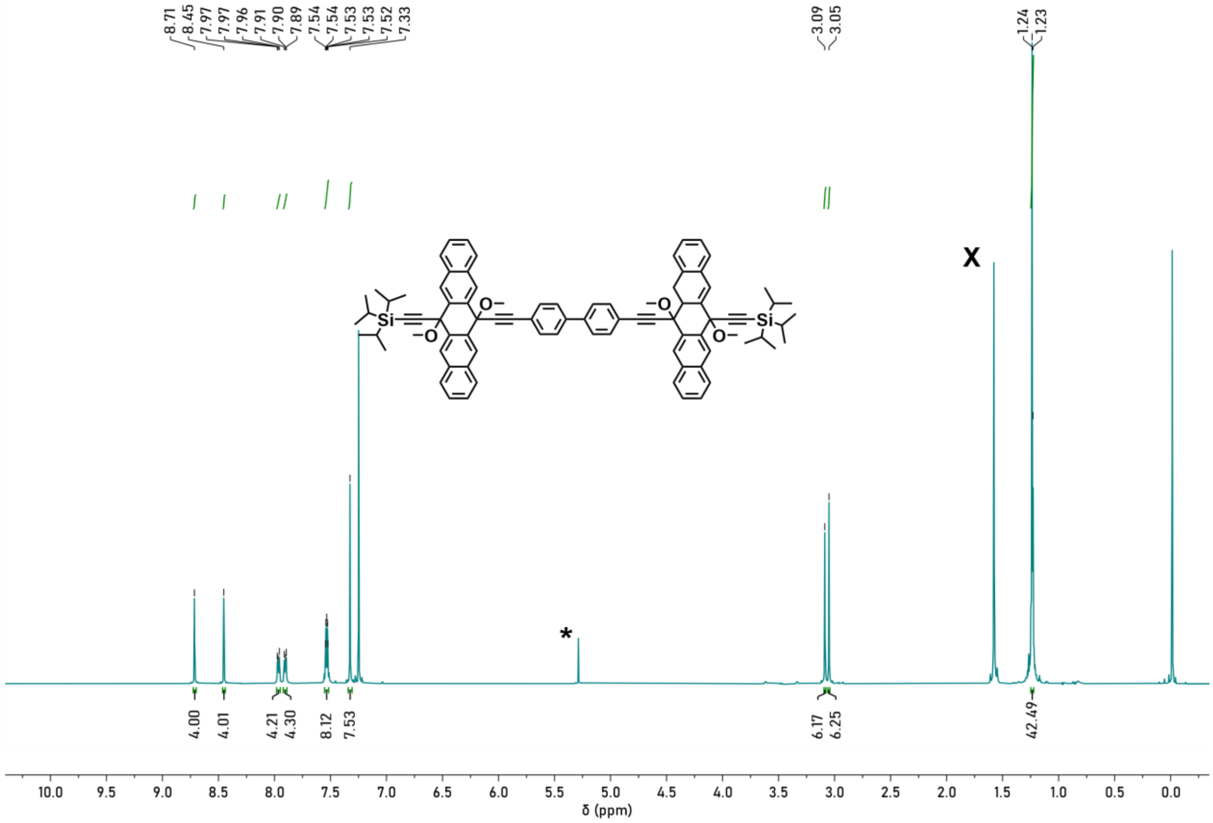

Figure S5.10. *^{1}H NMR spectrum of* ***7a (n=2)*** *in $CDCl_3$ [x = H_2O; * = dichloromethane]*

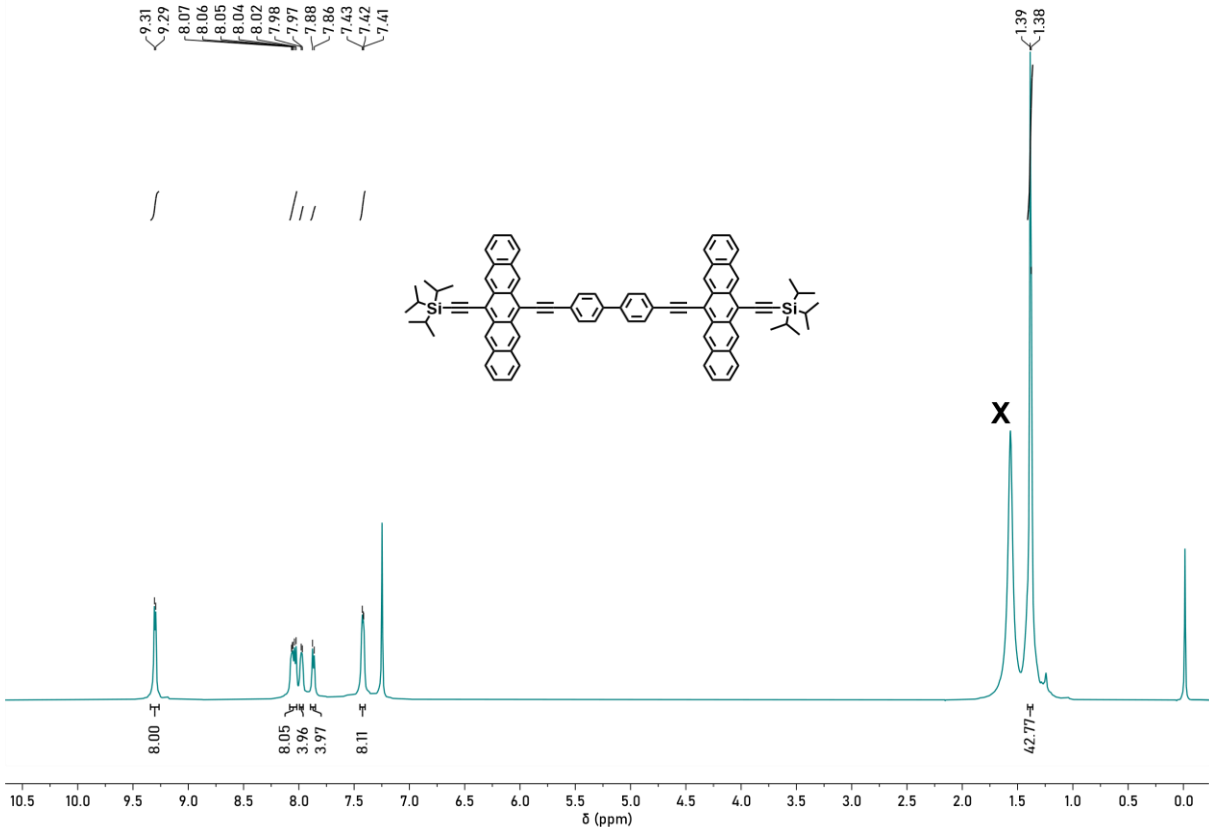

Figure S5.11. *^{1}H NMR spectrum of* ***6Ac-P2BP*** *in $CDCl_3$ [x = H_2O]*

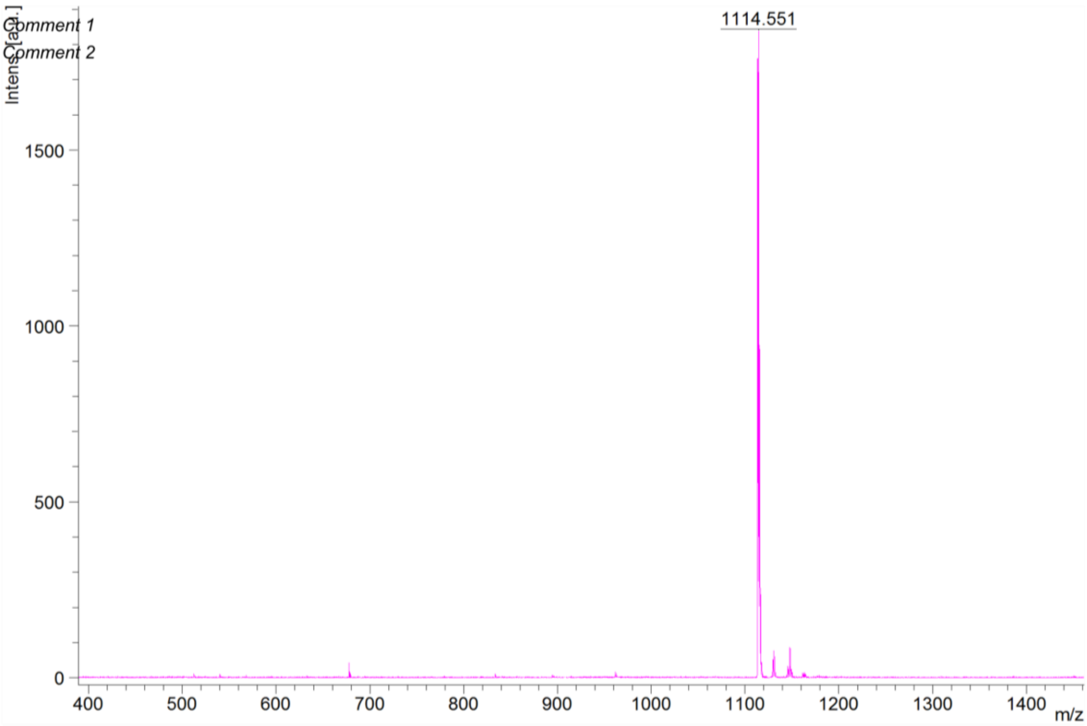

Acquisition Parameter

Date of acquisition	2022-05-12T17:17:06.574+02:00
Acquisition method name	D:\Methods\flexControlMethods\LP_700-2000_Da.par
Aquisition operation mode	Linear
Voltage polarity	POS
Number of shots	2000
Name of spectrum used for calibration	
Calibration reference list used	PeptideCalibStandard mono

Instrument Info

User	JNC
Instrument	FLEX-PC
Instrument type	autoflex

Bruker Daltonics flexAnalysis printed: 5/12/2022 5:44:15 PM

Figure S5.12. *MALDI-MS of **6Ac-P2BP***

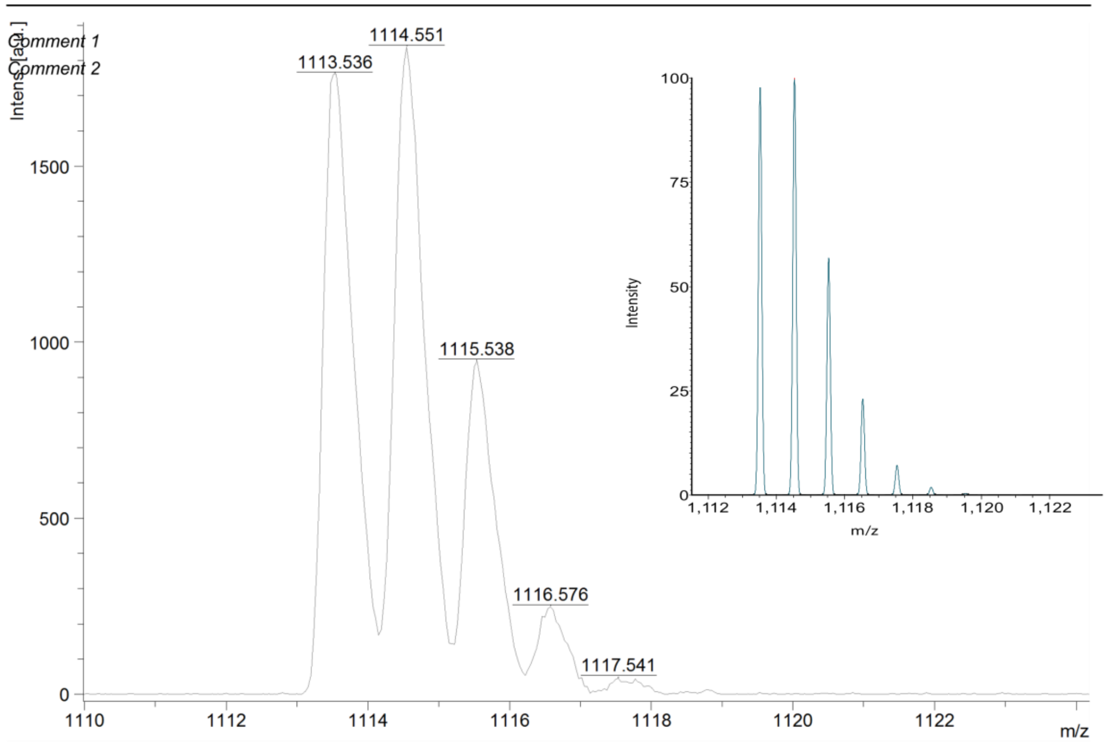

m/z	S/N	Quality Fac.	Res.	Intens.	Area
1113.536				1761	
1114.551	188		504	1838	2456
1115.538				944	
1116.576				248	
1117.541				43.4	

Acquisition Parameter

Date of acquisition	2022-05-12T17:17:06.574+02:00
Acquisition method name	D:\Methods\flexControlMethods\LP_700-2000_Da.par
Aquisition operation mode	Linear
Voltage polarity	POS
Number of shots	2000
Name of spectrum used for calibration	
Calibration reference list used	PeptideCalibStandard mono

Instrument Info

User	JNC
Instrument	FLEX-PC

Bruker Daltonics flexAnalysis printed: 7/8/2022 4:50:39 PM

Figure S5.13. *MALDI-MS isotopic distribution of* ***6Ac-P2BP****, ([M-H]$^+$). Simulated isotopic distribution of 3a (inset).*

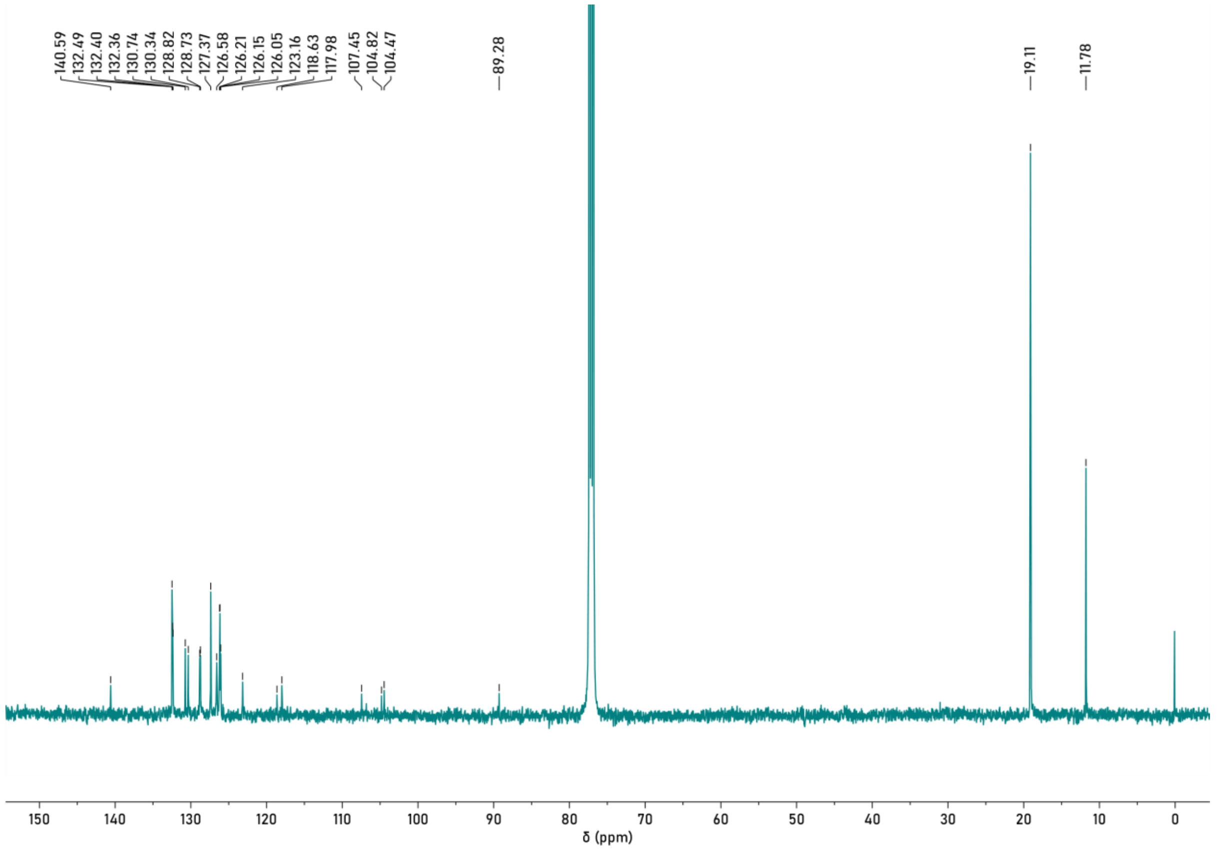

Figure S5.14. *^{13}C NMR spectrum of* ***6Ac-P2BP*** *in $CDCl_3$*

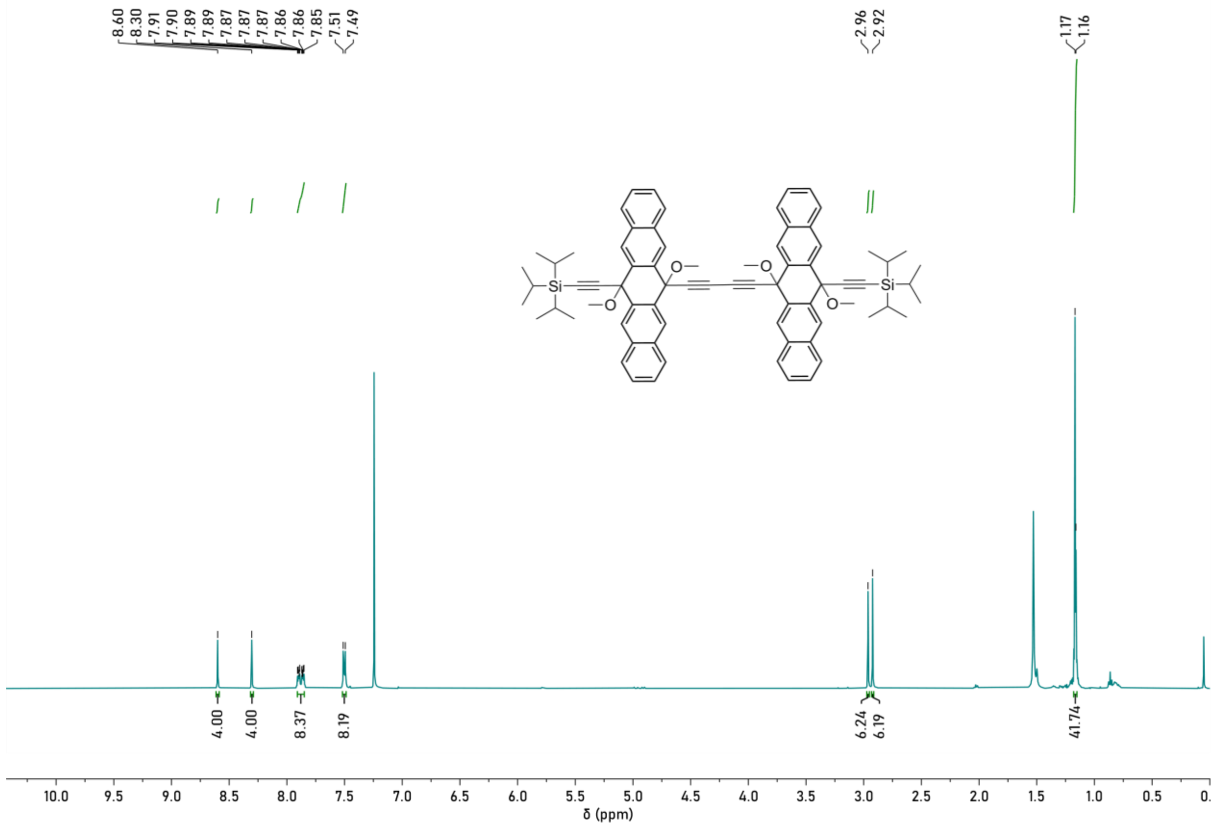

Figure S5.15. *^{1}H NMR spectrum of **7b(n=0)** in $CDCl_3$ [x = H_2O]*

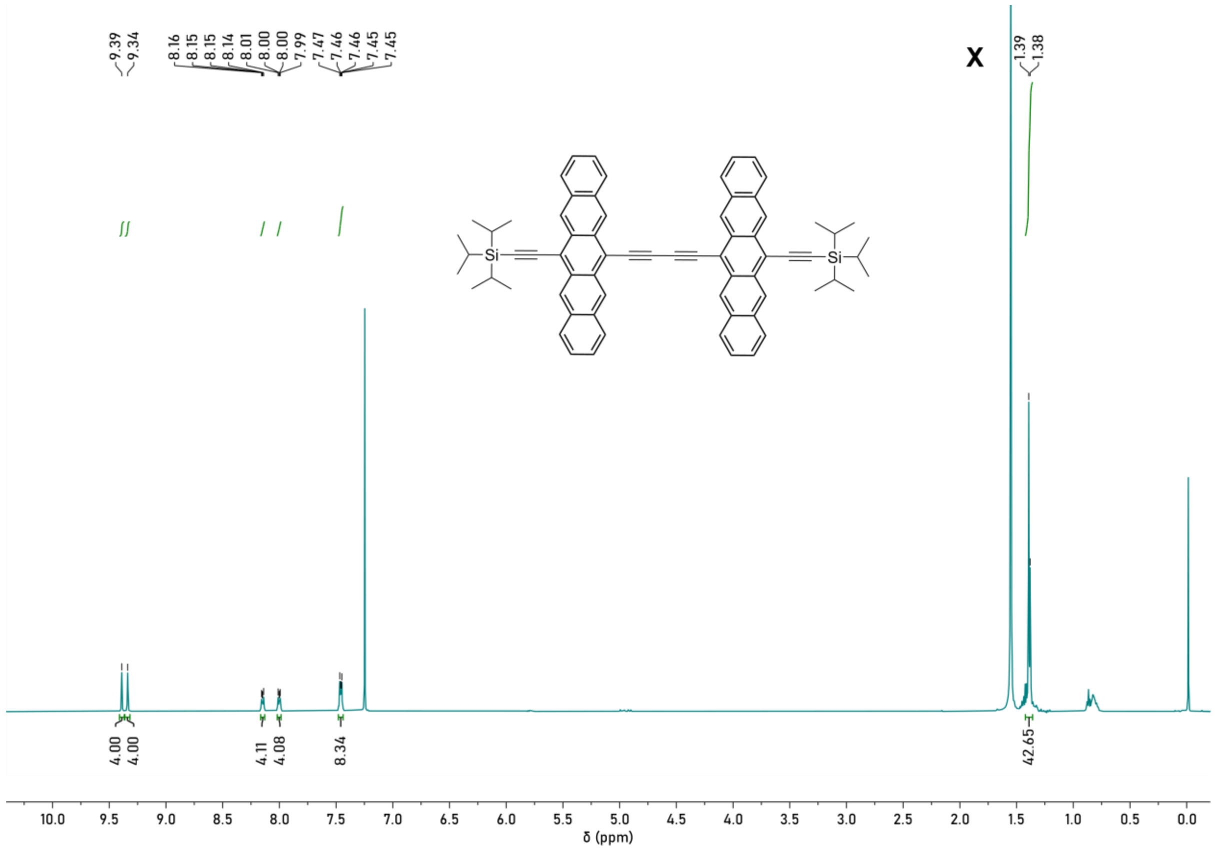

Figure S5.16. *^{1}H NMR spectrum of **6Ac-P2** in $CDCl_3$ [x = H_2O]*

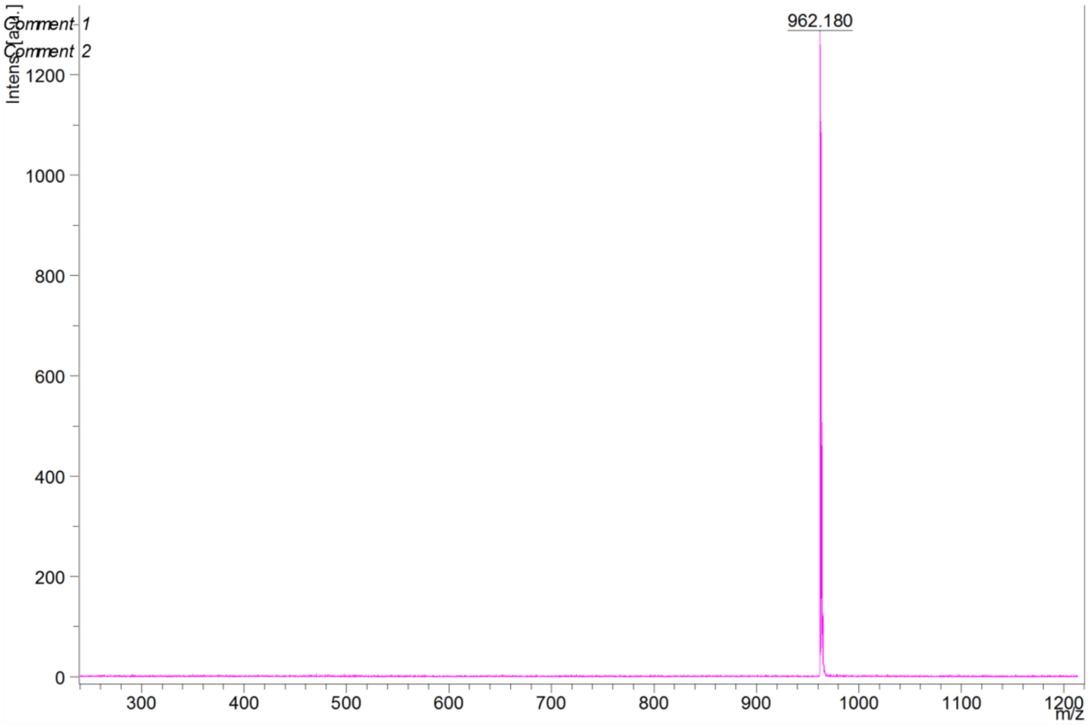

Acquisition Parameter

Date of acquisition	2020-02-05T16:31:12.520+05:30
Acquisition method name	D:\Methods\flexControlMethods\LP_700-2000_Da.par
Aquisition operation mode	Linear
Voltage polarity	POS
Number of shots	500
Name of spectrumused for calibration	
Calibration reference list used	PeptideCalibStandard mono

Instrument Info

User	JNCASR
Instrument	ATS-00704
Instrument type	autoflex

Bruker Daltonics flexAnalysis printed: 2/26/2020 4:53:37 PM

Figure S5.17. *MALDI-MS of **6Ac-P2***

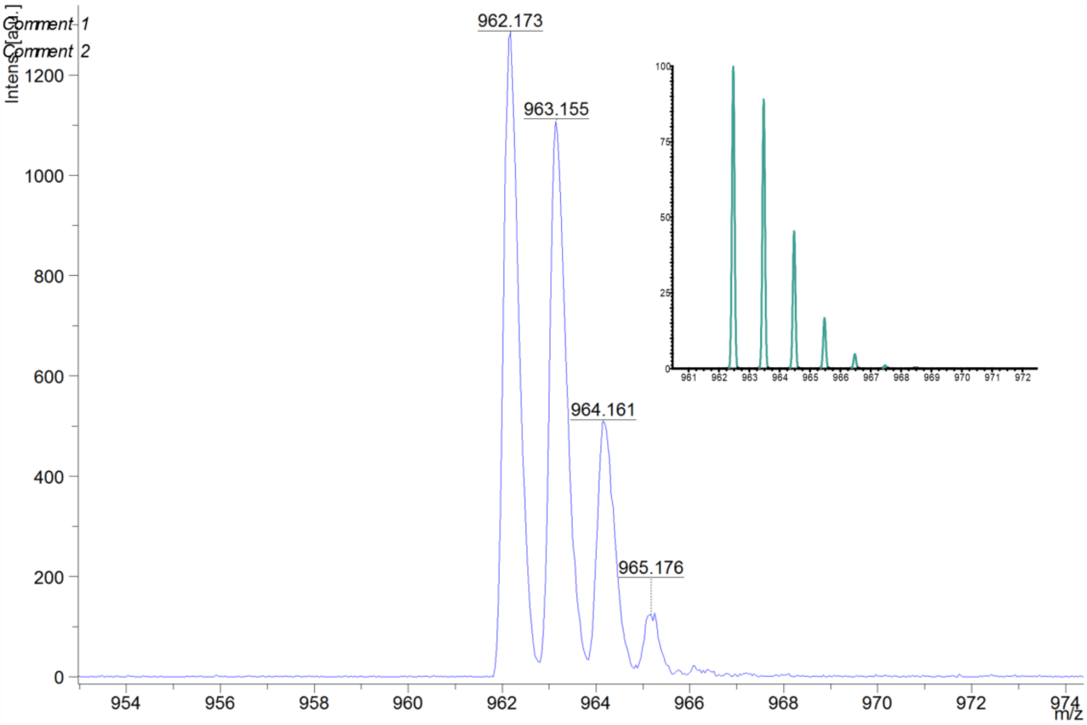

m/z	S/N	Quality Fac.	Res.	Intens.	Area
962.173				1282	
963.155	158		717	1108	1272
964.161				507	
965.176				123	

Acquisition Parameter

Date of acquisition	2020-02-05T16:31:12.520+05:30
Acquisition method name	D:\Methods\flexControlMethods\LP_700-2000_Da.par
Aquisition operation mode	Linear
Voltage polarity	POS
Number of shots	500
Name of spectrumused for calibration	
Calibration reference list used	PeptideCalibStandard mono

Instrument Info

User	JNCASR
Instrument	ATS-00704
Instrument type	autoflex

Bruker Daltonics flexAnalysis printed: 7/8/2022 4:43:42 PM

Figure S5.18. *MALDI-MS isotopic distribution of **6Ac-P2**, (M⁺). Simulated isotopic distribution of 3r (inset).*

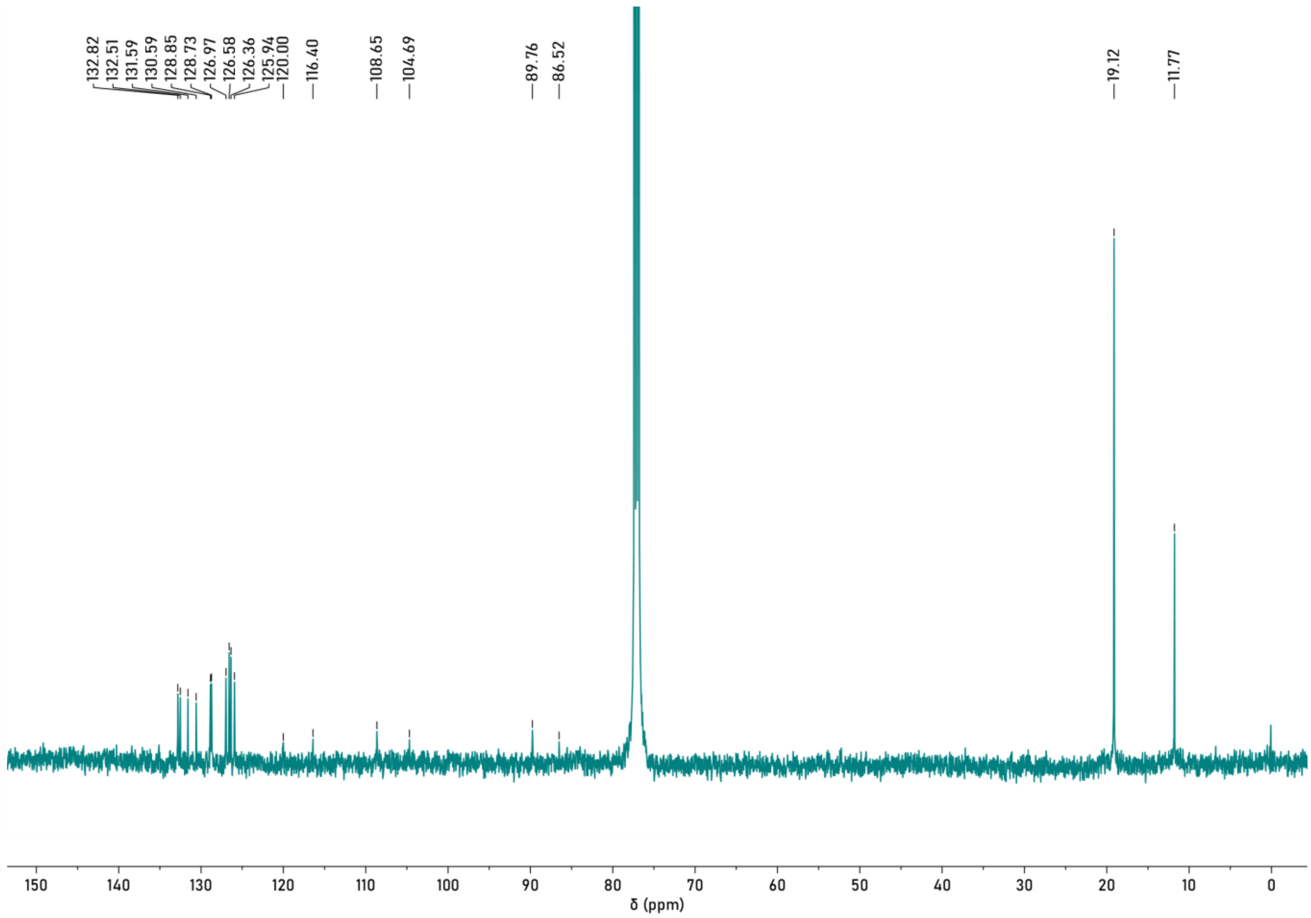

Figure S5.19. *^{13}C NMR spectrum of **6Ac-P2** in $CDCl_3$*

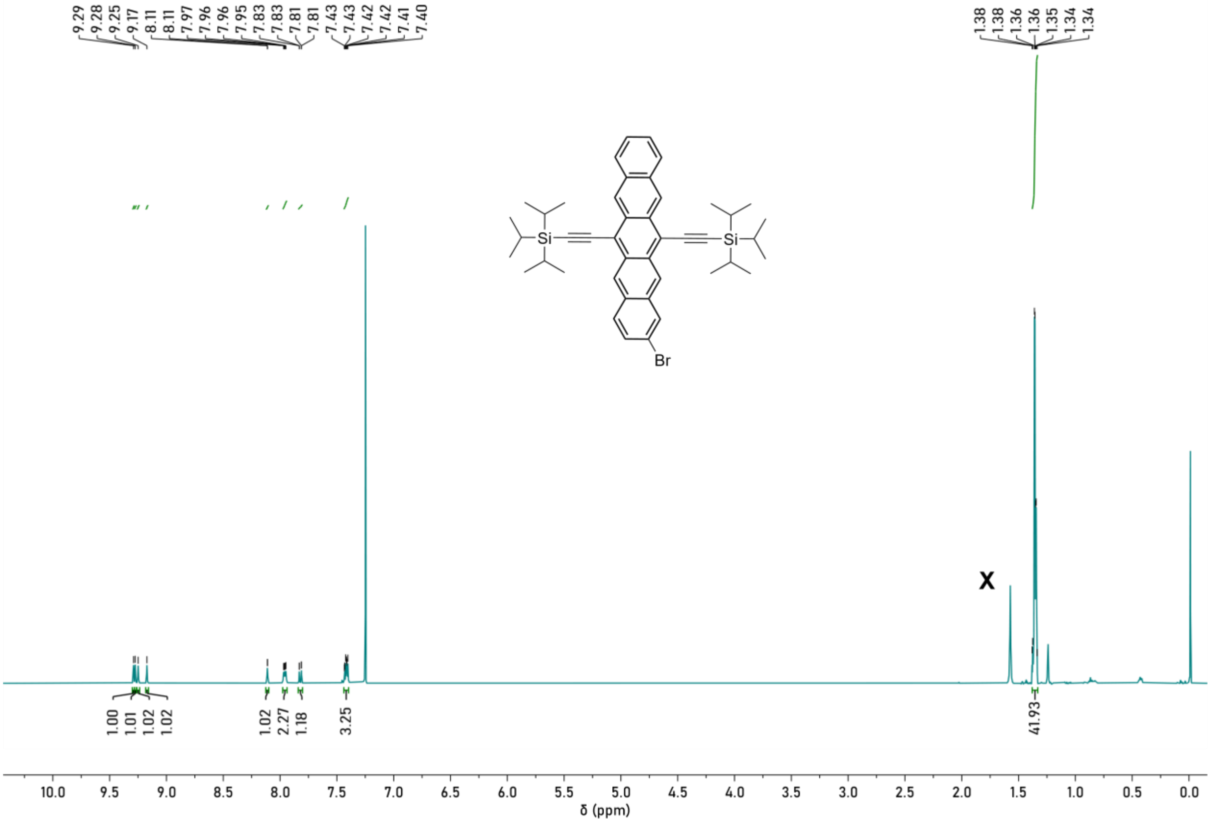

Figure S5.20. ^{1}H *NMR spectrum of **1** in $CDCl_3$ [x = H_2O]*

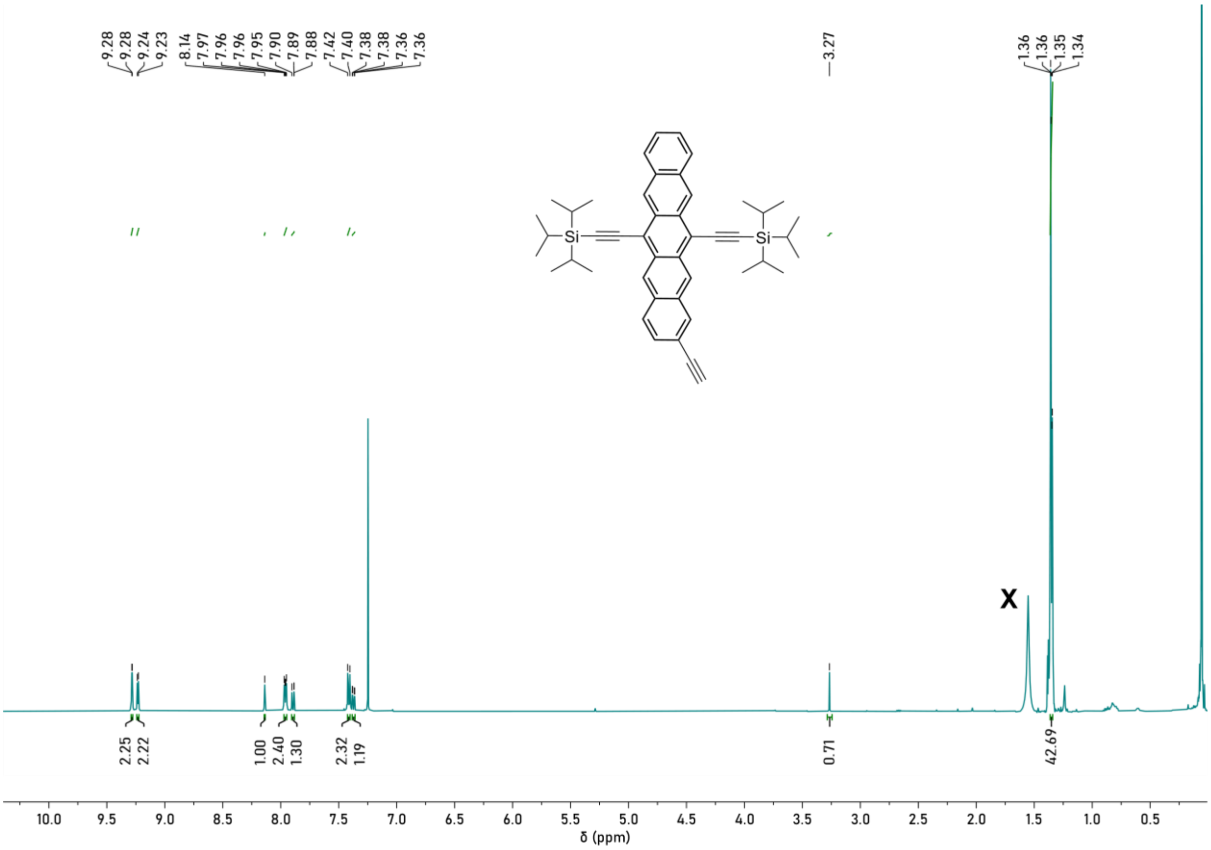

Figure S5.21. ^{1}H *NMR spectrum of **2** in $CDCl_3$ [x = H_2O]*

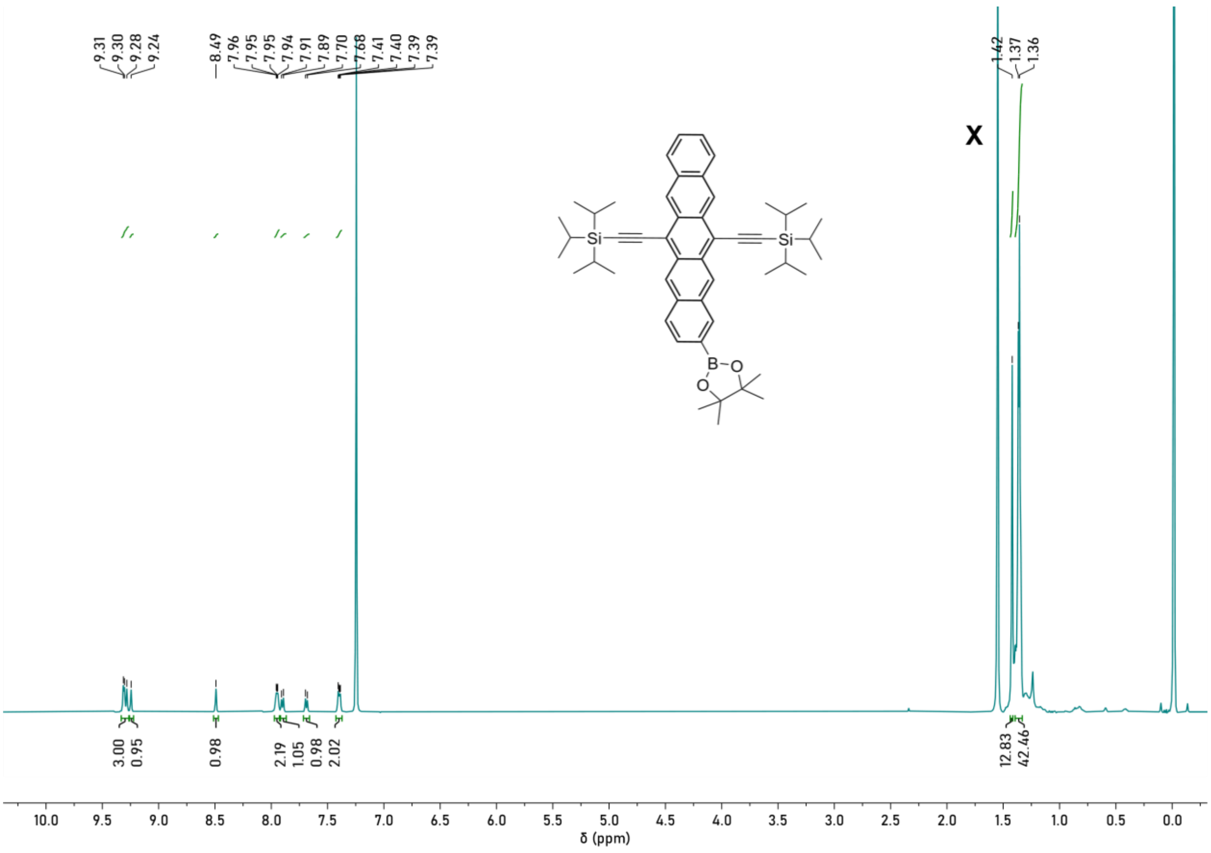

Figure S5.22. *^{1}H NMR spectrum of **3** in $CDCl_3$ [x = H_2O]*

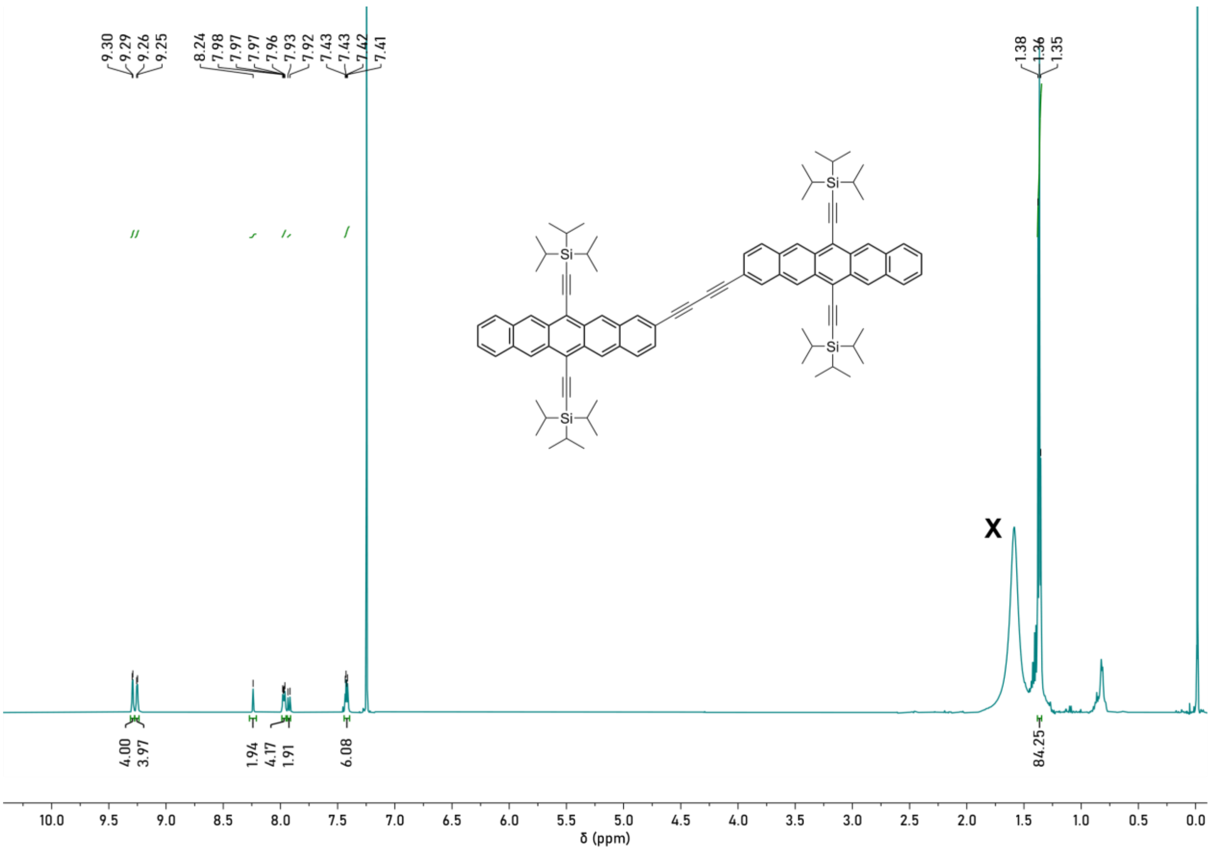

Figure S5.23. *^{1}H NMR spectrum of* ***2Ac-P2*** *in $CDCl_3$ [x = H_2O]*

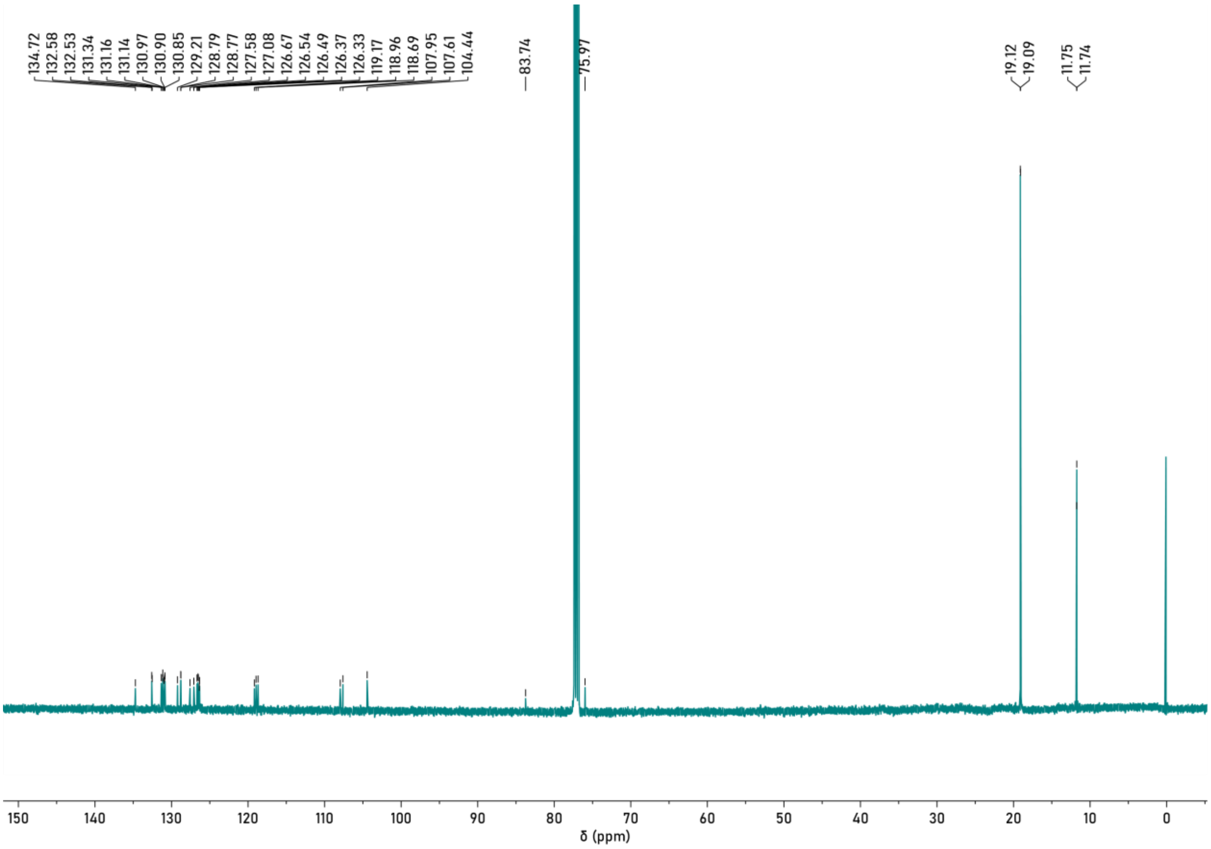

Figure S5.24. *^{13}C NMR spectrum of* ***2Ac-P2*** *in $CDCl_3$*

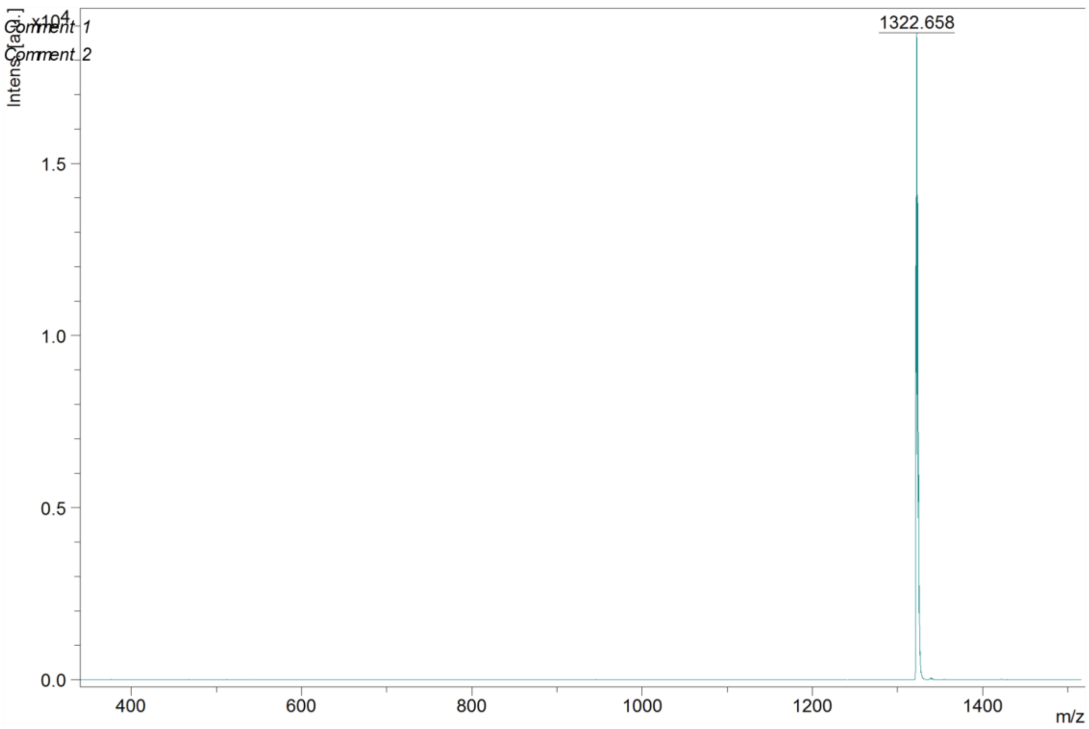

Acquisition Parameter

Date of acquisition	2022-05-12T17:17:34.326+02:00
Acquisition method name	D:\Methods\flexControlMethods\LP_700-2000_Da.par
Aquisition operation mode	Linear
Voltage polarity	POS
Number of shots	2000
Name of spectrum used for calibration	
Calibration reference list used	PeptideCalibStandard mono

Instrument Info

User	JNC
Instrument	FLEX-PC
Instrument type	autoflex

Bruker Daltonics flexAnalysis printed: 5/12/2022 5:45:01 PM

Figure S5.25. *MALDI-MS of* ***2Ac-P2***

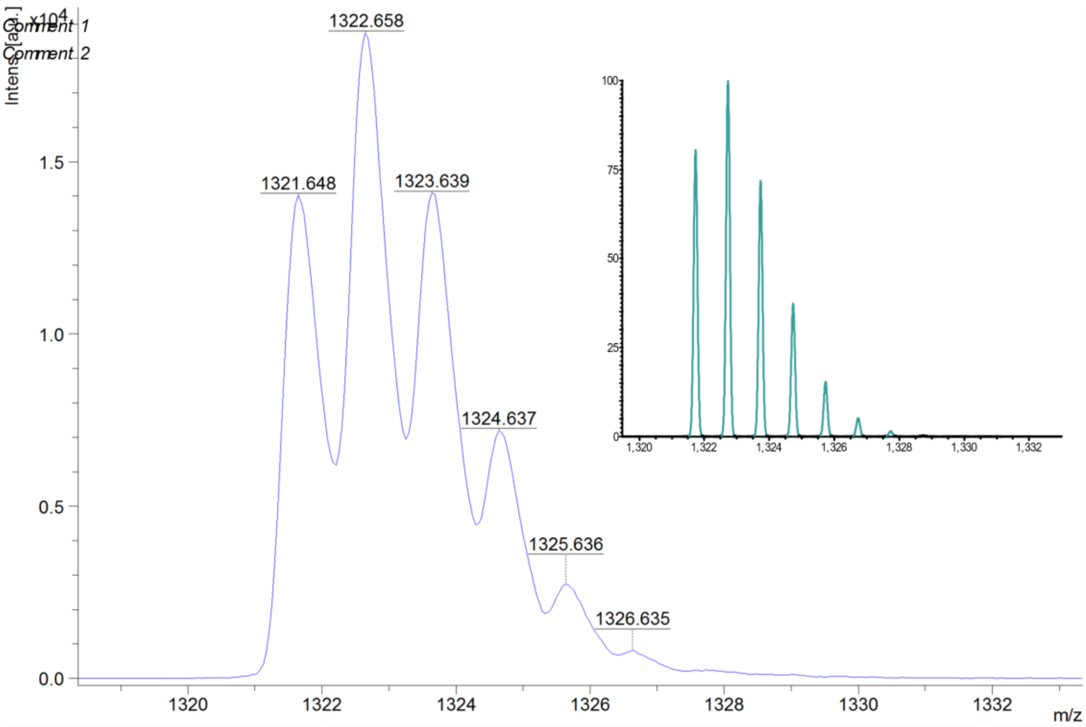

m/z	S/N	Quality Fac.	Res.	Intens.	Area
1321.648				14003	
1322.658	1201		530	18738	40275
1323.639				14088	
1324.637				7193	
1325.636				2741	
1326.635				823	

Acquisition Parameter

Date of acquisition	2022-05-12T17:17:34.326+02:00
Acquisition method name	D:\Methods\flexControlMethods\LP_700-2000_Da.par
Aquisition operation mode	Linear
Voltage polarity	POS
Number of shots	2000
Name of spectrum used for calibration	
Calibration reference list used	PeptideCalibStandard mono

Instrument Info

User	JNC
Instrument	FLEX-PC
Instrument type	autoflex

Bruker Daltonics flexAnalysis printed: 11/16/2022 8:56:41 AM

Figure S5.26. *MALDI-MS isotopic distribution of **2Ac-P2**, ([M-H]$^+$). Simulated isotopic distribution (inset).*

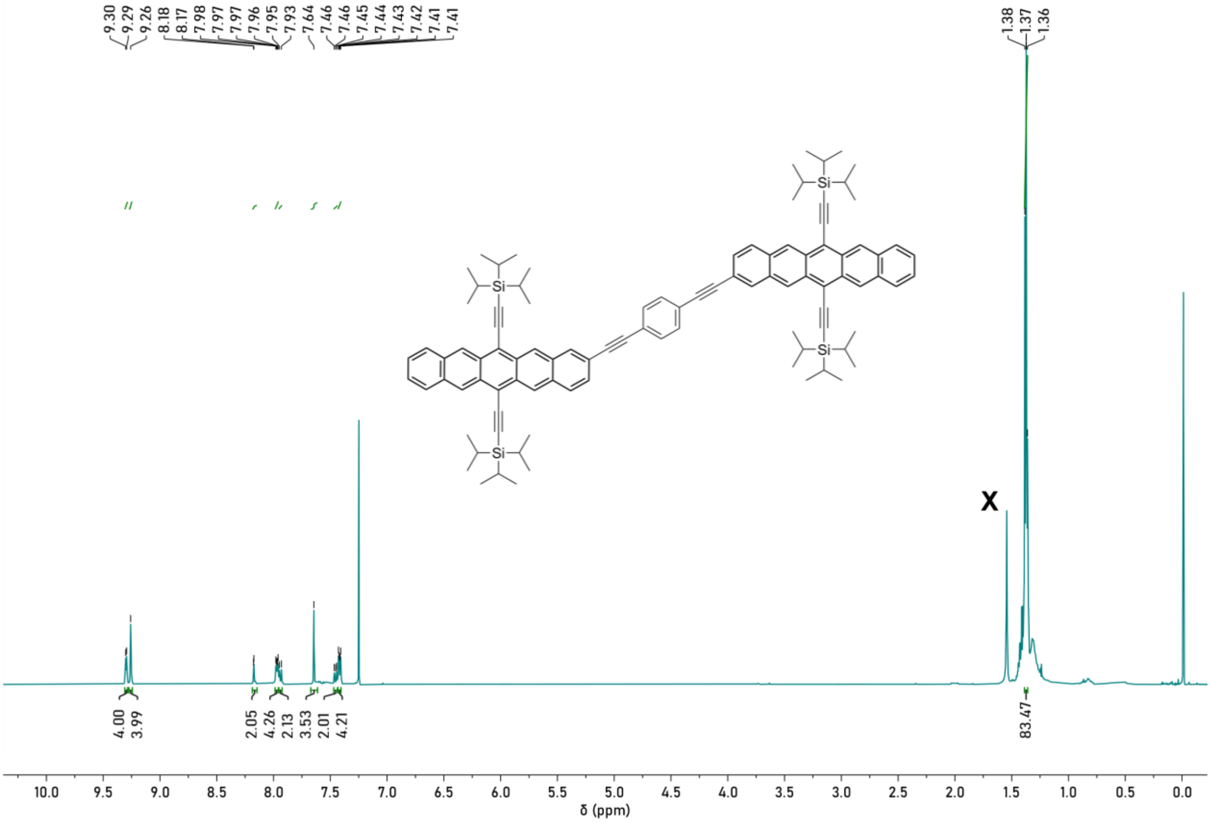

Figure S5.27. *^{1}H NMR spectrum of **2Ac-P2Ph** in $CDCl_3$ [x = H_2O]*

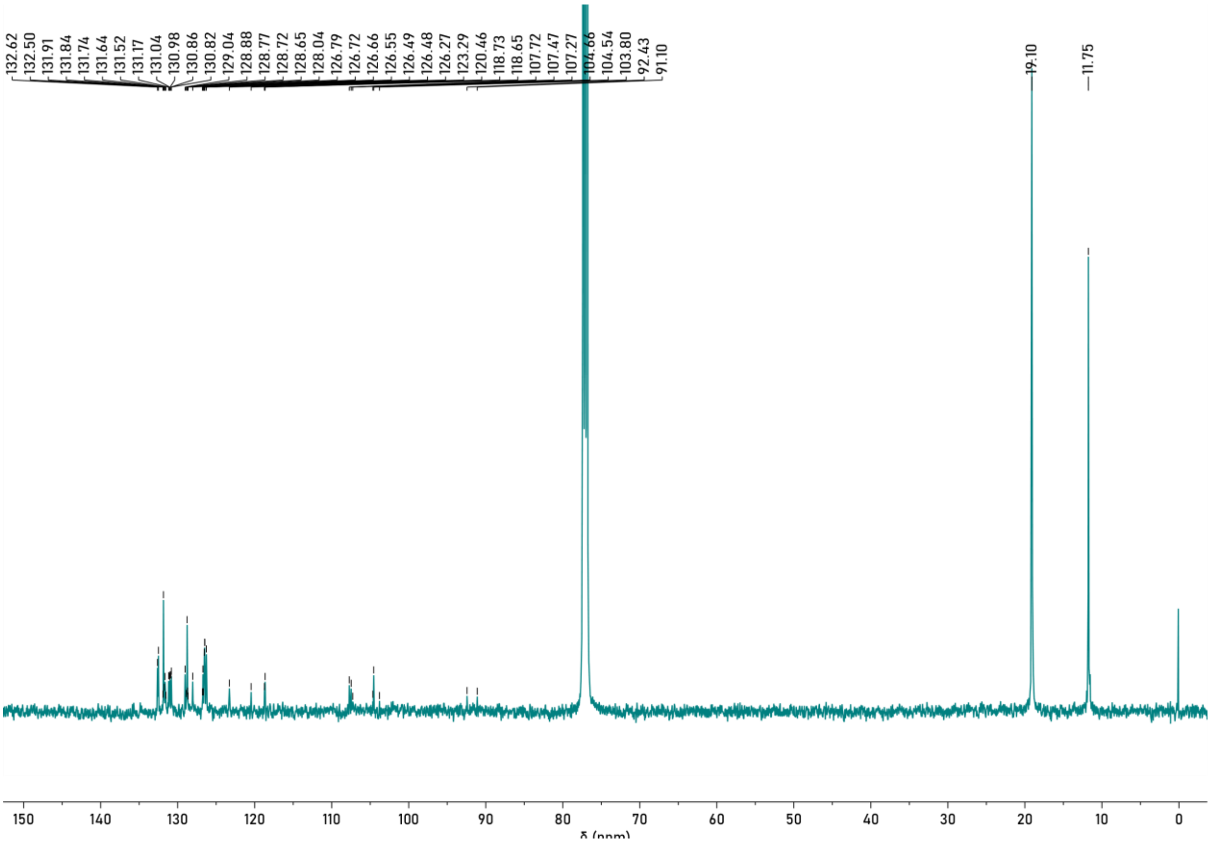

Figure S5.28. *^{13}C NMR spectrum of **2Ac-P2Ph** in $CDCl_3$*

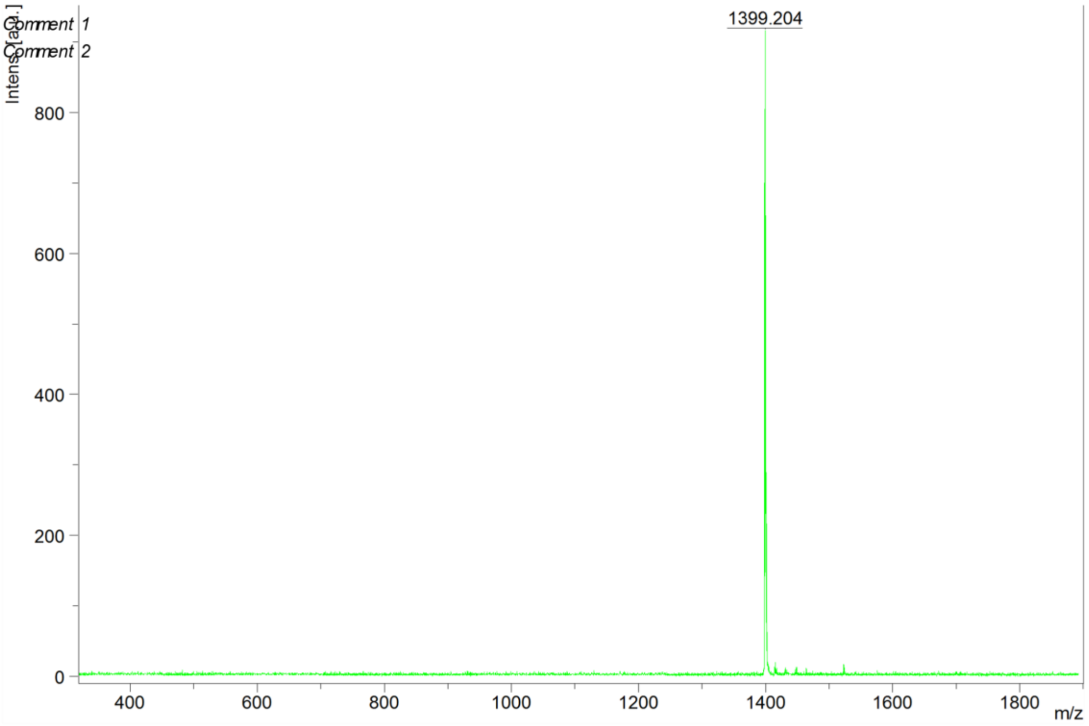

Acquisition Parameter

Date of acquisition	2022-02-01T17:05:45.737+05:30
Acquisition method name	D:\Methods\flexControlMethods\LP_700-2000_Da.par
Aquisition operation mode	Linear
Voltage polarity	POS
Number of shots	2000
Name of spectrumused for calibration	
Calibration reference list used	PeptideCalibStandard mono

Instrument Info

User	JNCASR
Instrument	ATS-00704
Instrument type	autoflex

Bruker Daltonics flexAnalysis printed: 2/1/2022 5:13:22 PM

Figure S5.29. *MALDI-MS of* ***2Ac-P2Ph***

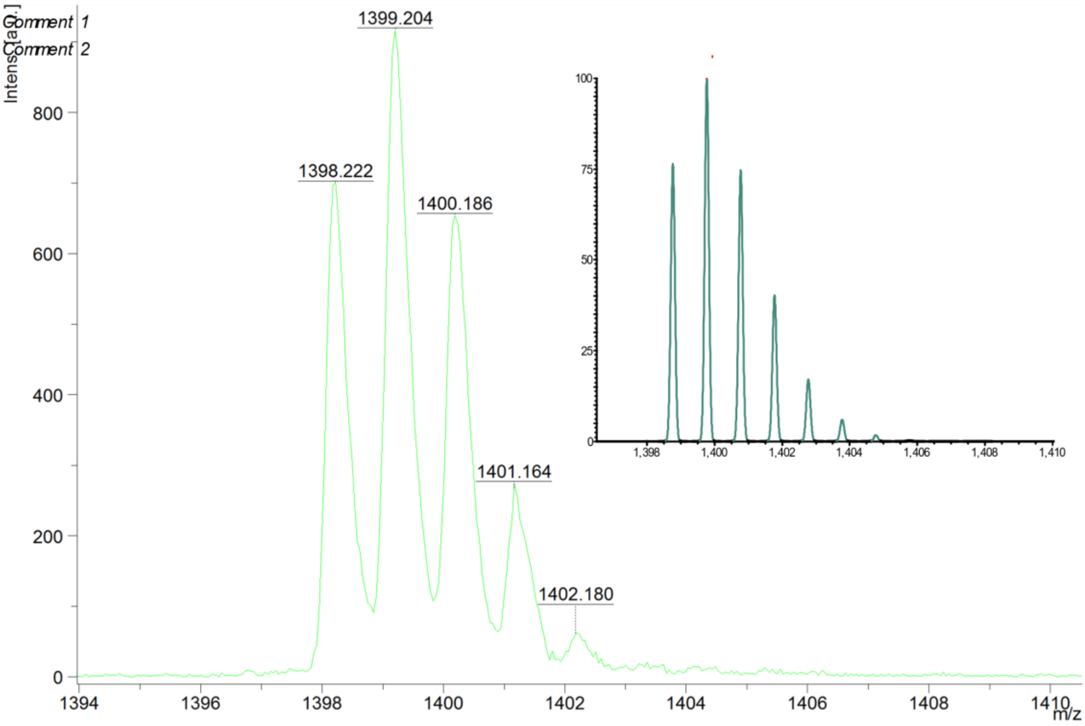

m/z	S/N	Quality Fac.	Res.	Intens.	Area
1398.222				700	
1399.204	135		608	917	1314
1400.186				654	
1401.164				267	
1402.180				60.4	

Acquisition Parameter

Date of acquisition	2022-02-01T17:05:45.737+05:30
Acquisition method name	D:\Methods\flexControlMethods\LP_700-2000_Da.par
Aquisition operation mode	Linear
Voltage polarity	POS
Number of shots	2000
Name of spectrum used for calibration	
Calibration reference list used	PeptideCalibStandard mono

Instrument Info

User	JNCASR
Instrument	ATS-00704
Instrument type	autoflex

Figure S5.30. *MALDI-MS isotopic distribution of **2Ac-P2Ph** (M+). Simulated isotopic distribution (inset).*

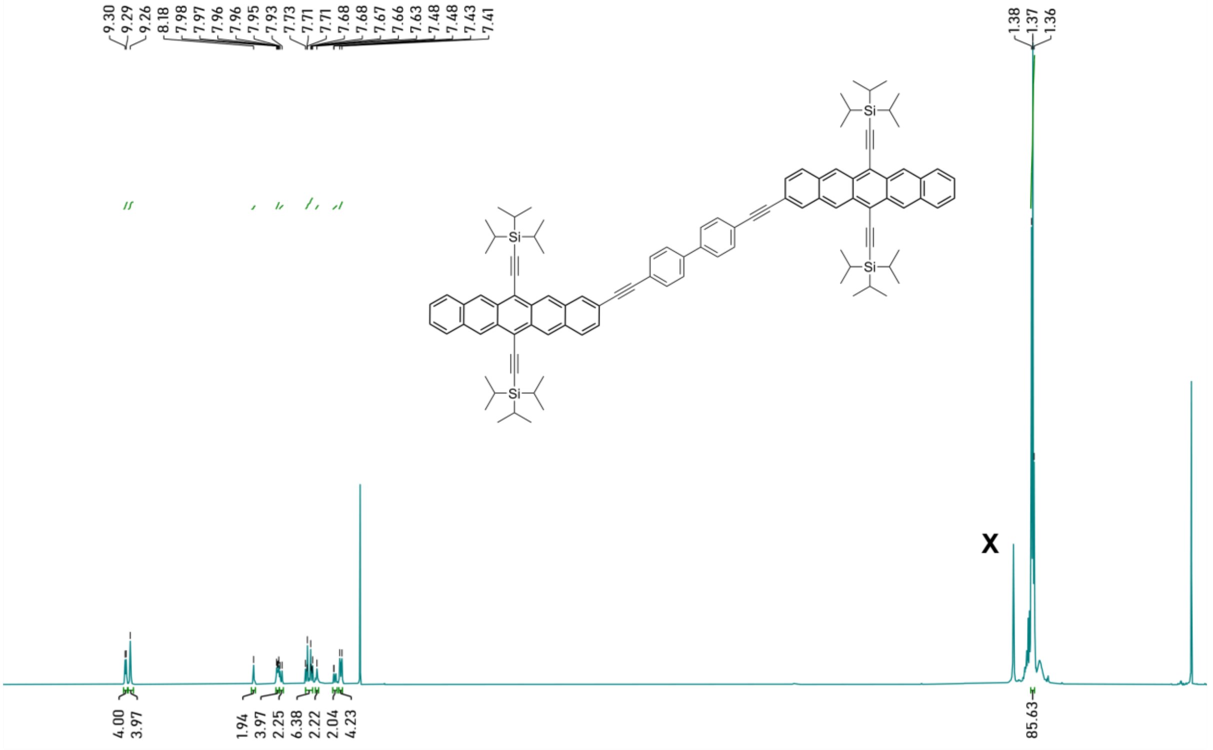

Figure S5.31. ^{1}H *NMR spectrum of* ***2Ac-P2BP*** *in* $CDCl_3$ *[x =* H_2O*]*

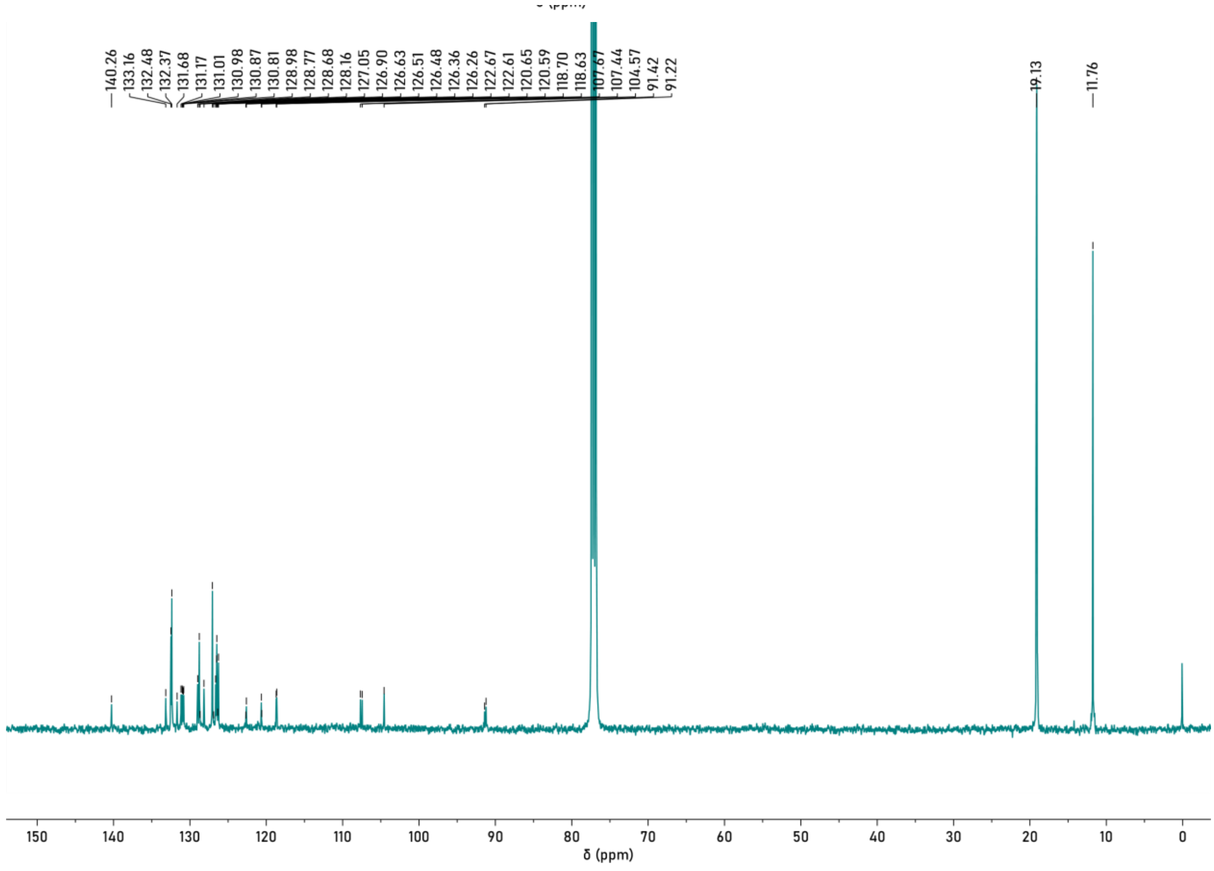

Figure S5.32. ^{13}C *NMR spectrum of* ***2Ac-P2BP*** *in* $CDCl_3$

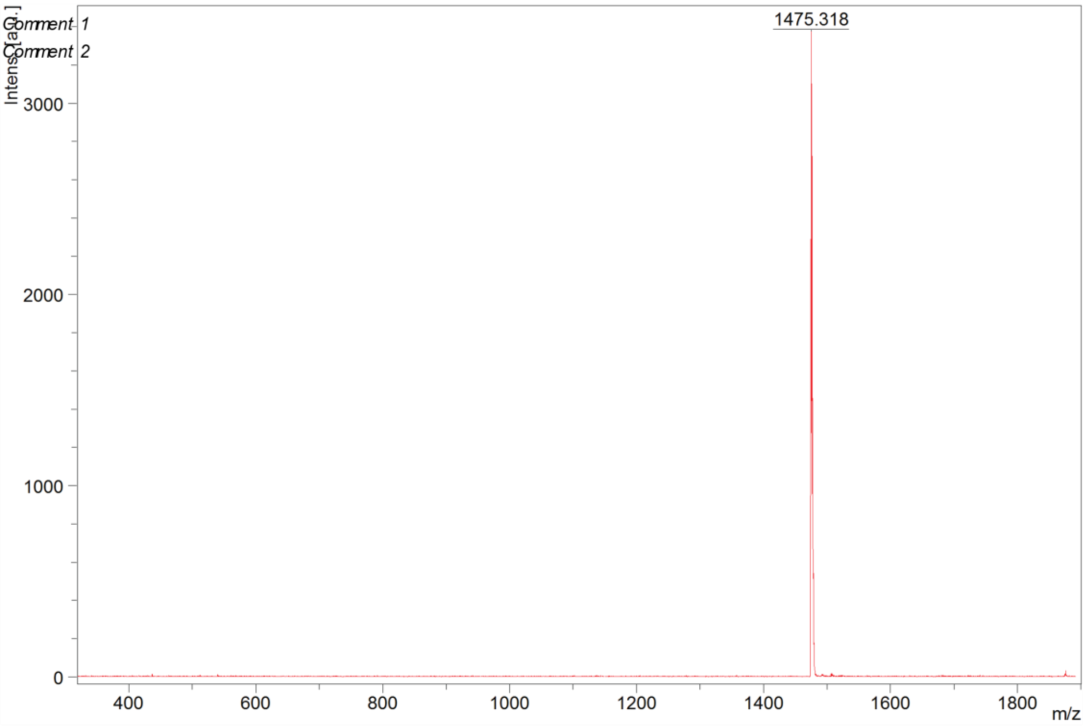

Acquisition Parameter

Date of acquisition	2022-02-01T17:06:39.729+05:30
Acquisition method name	D:\Methods\flexControlMethods\LP_700-2000_Da.par
Aquisition operation mode	Linear
Voltage polarity	POS
Number of shots	2000
Name of spectrum used for calibration	
Calibration reference list used	PeptideCalibStandard mono

Instrument Info

User	JNCASR
Instrument	ATS-00704
Instrument type	autoflex

Bruker Daltonics flexAnalysis printed: 2/1/2022 5:13:11 PM

Figure S5.33. *MALDI-MS of* ***2Ac-P2BP***

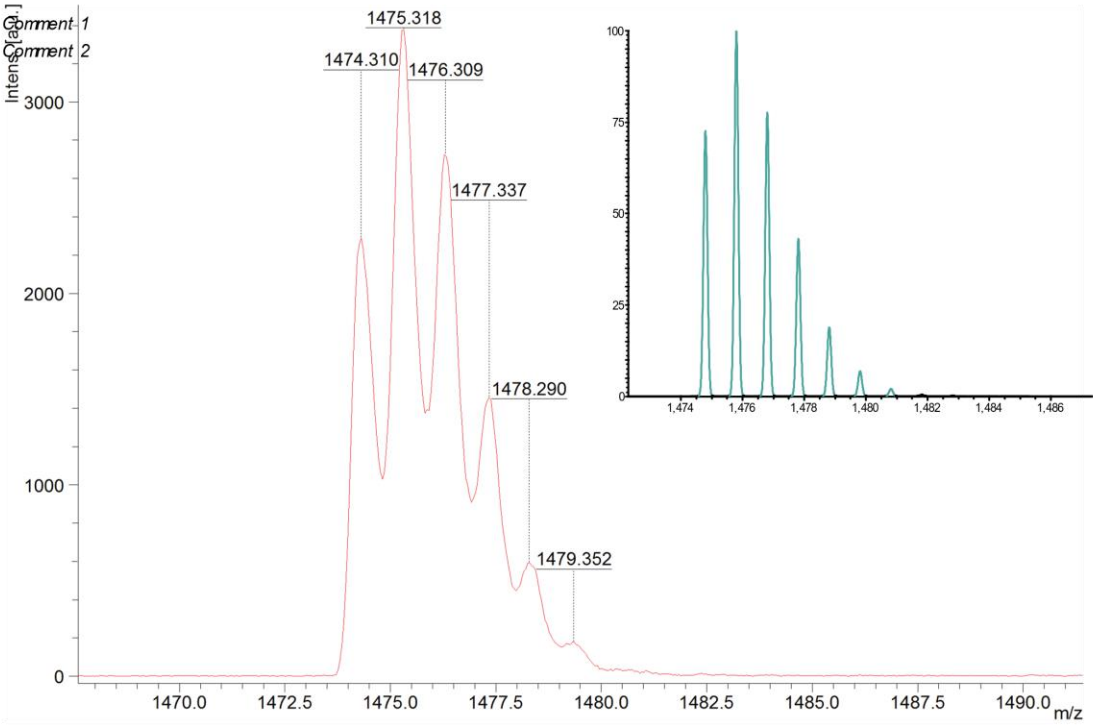

m/z	S/N	Quality Fac.	Res.	Intens.	Area
1474.310				2290	
1475.318				3377	
1476.309	398		597	2727	7374
1477.337				1457	
1478.290				592	
1479.352				178	

Acquisition Parameter

Date of acquisition	2022-02-01T17:06:39.729+05:30
Acquisition method name	D:\Methods\flexControlMethods\LP_700-2000_Da.par
Aquisition operation mode	Linear
Voltage polarity	POS
Number of shots	2000
Name of spectrumused for calibration	
Calibration reference list used	PeptideCalibStandard mono

Instrument Info

User	JNCASR
Instrument	ATS-00704
Instrument type	autoflex

Bruker Daltonics flexAnalysis printed: 11/16/2022 8:54:08 AM

Figure S5.34. *MALDI-MS isotopic distribution of* ***2Ac-P2BP****, (M^+). Simulated isotopic distribution (inset).*

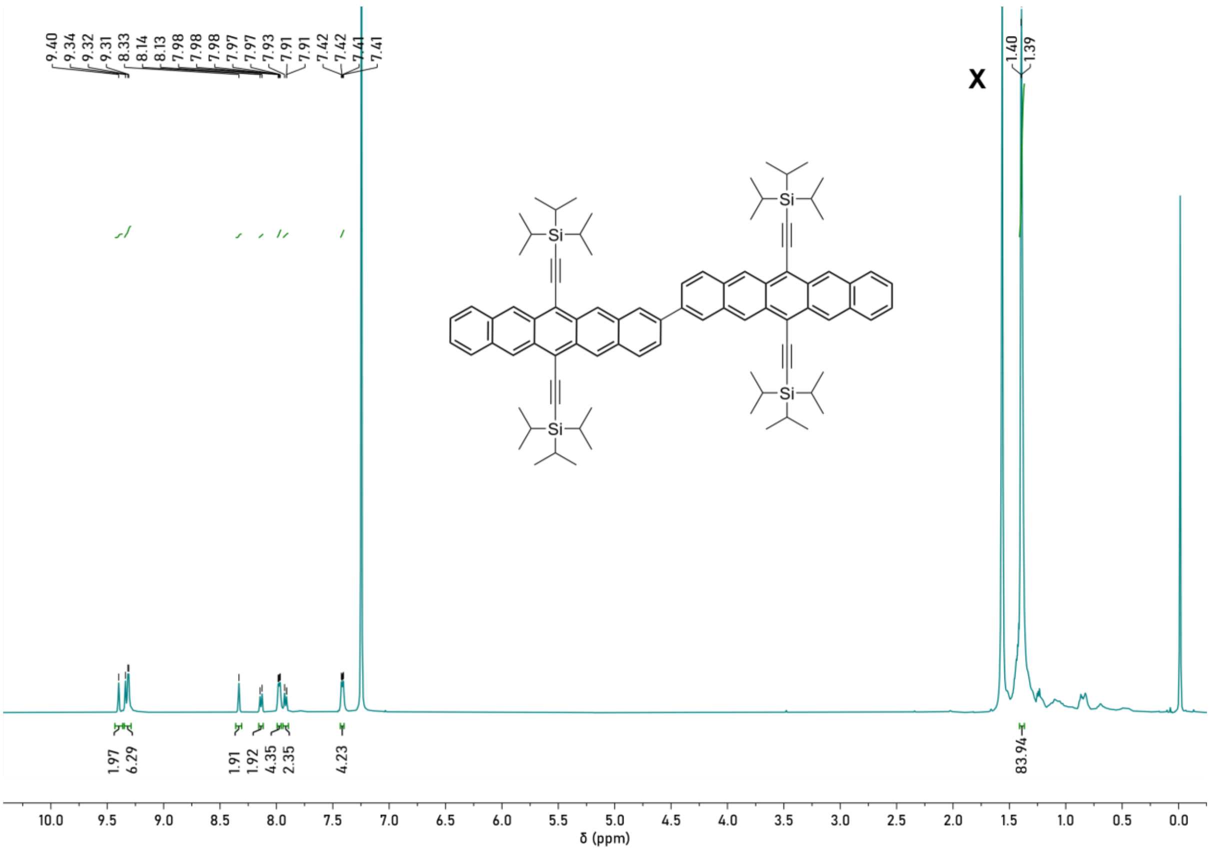

Figure S5.35. *^1H NMR spectrum of **2-P2** in $CDCl_3$ [x = H_2O]*

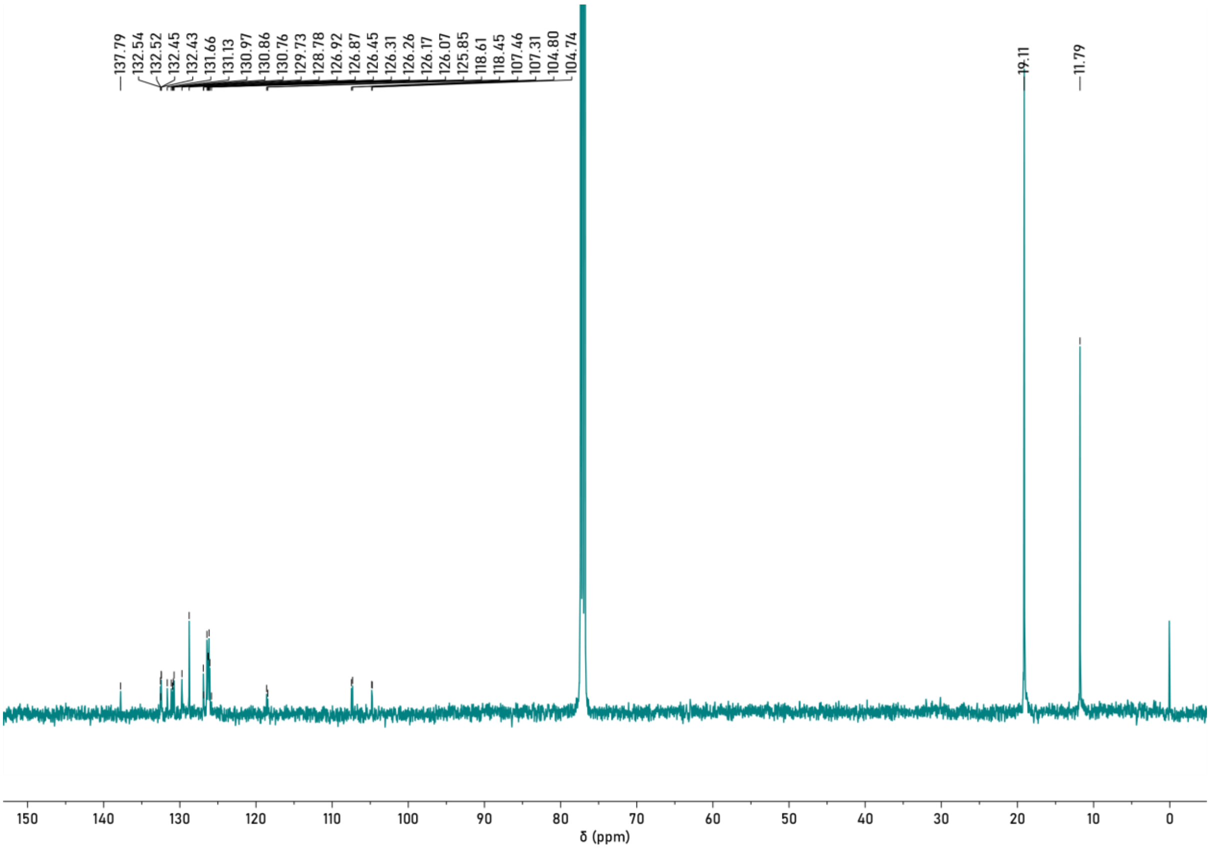

Figure S5.36. *^{13}C NMR spectrum of **2-P2** in $CDCl_3$*

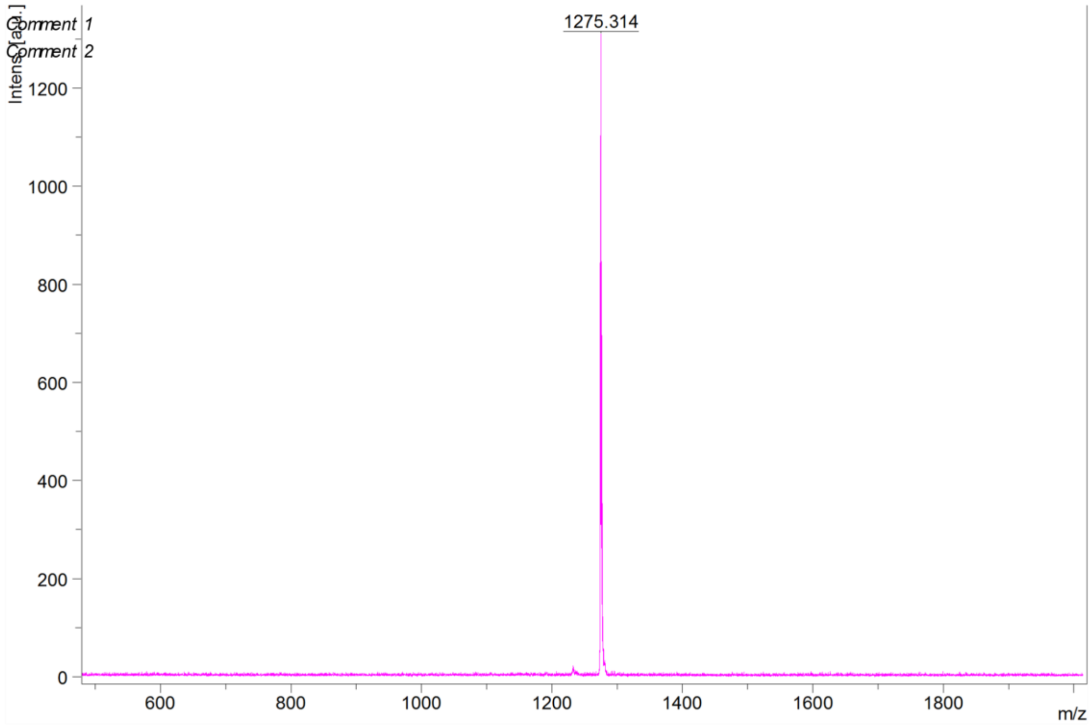

Acquisition Parameter

Date of acquisition	2021-09-28T12:55:11.141+05:30
Acquisition method name	D:\Methods\flexControlMethods\LP_700-2000_Da.par
Aquisition operation mode	Linear
Voltage polarity	POS
Number of shots	2000
Name of spectrum used for calibration	
Calibration reference list used	PeptideCalibStandard mono

Instrument Info

User	JNCASR
Instrument	ATS-00704
Instrument type	autoflex

Bruker Daltonics flexAnalysis printed: 9/28/2021 5:14:28 PM

Figure S5.37. *MALDI-MS of **2-P2***

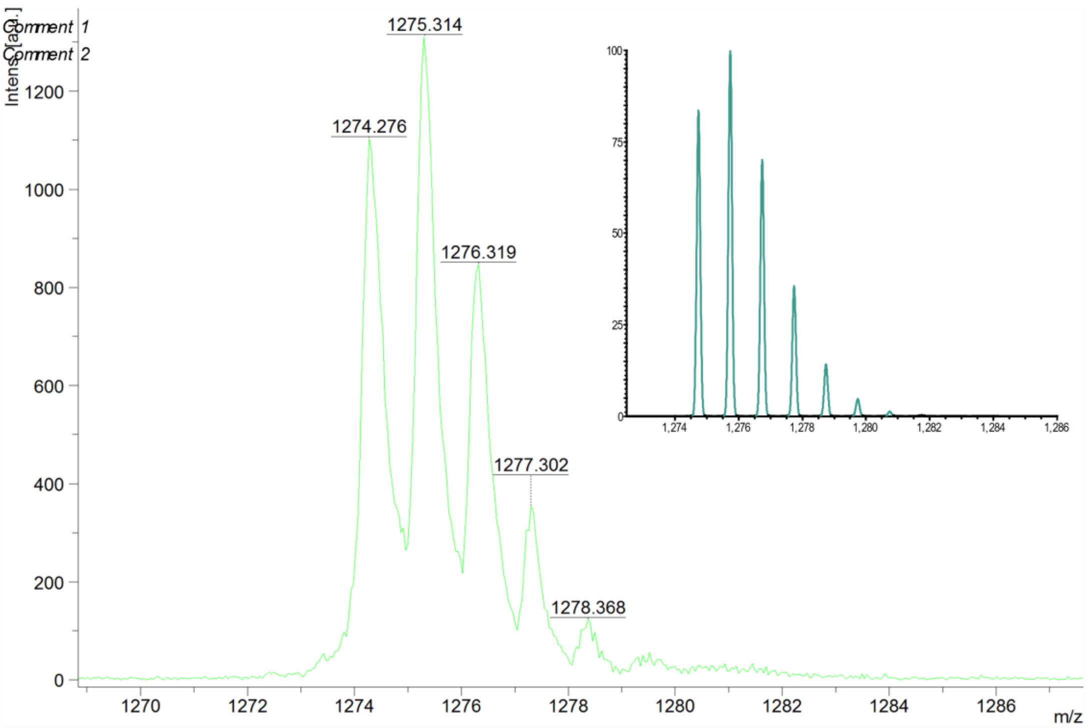

m/z	S/N	Quality Fac.	Res.	Intens.	Area
1274.276				1103	
1275.314	180		554	1311	2098
1276.319				846	
1277.302				353	
1278.368				123	

Acquisition Parameter

Date of acquisition	2021-09-28T12:55:11.141+05:30
Acquisition method name	D:\Methods\flexControlMethods\LP_700-2000_Da.par
Aquisition operation mode	Linear
Voltage polarity	POS
Number of shots	2000
Name of spectrum used for calibration	
Calibration reference list used	PeptideCalibStandard mono

Instrument Info

User	JNCASR
Instrument	ATS-00704
Instrument type	autoflex

Bruker Daltonics flexAnalysis printed: 9/5/2022 5:43:30 PM

Figure S5.38. *MALDI-MS isotopic distribution of **2-P2**, (M^+). Simulated isotopic distribution (inset).*

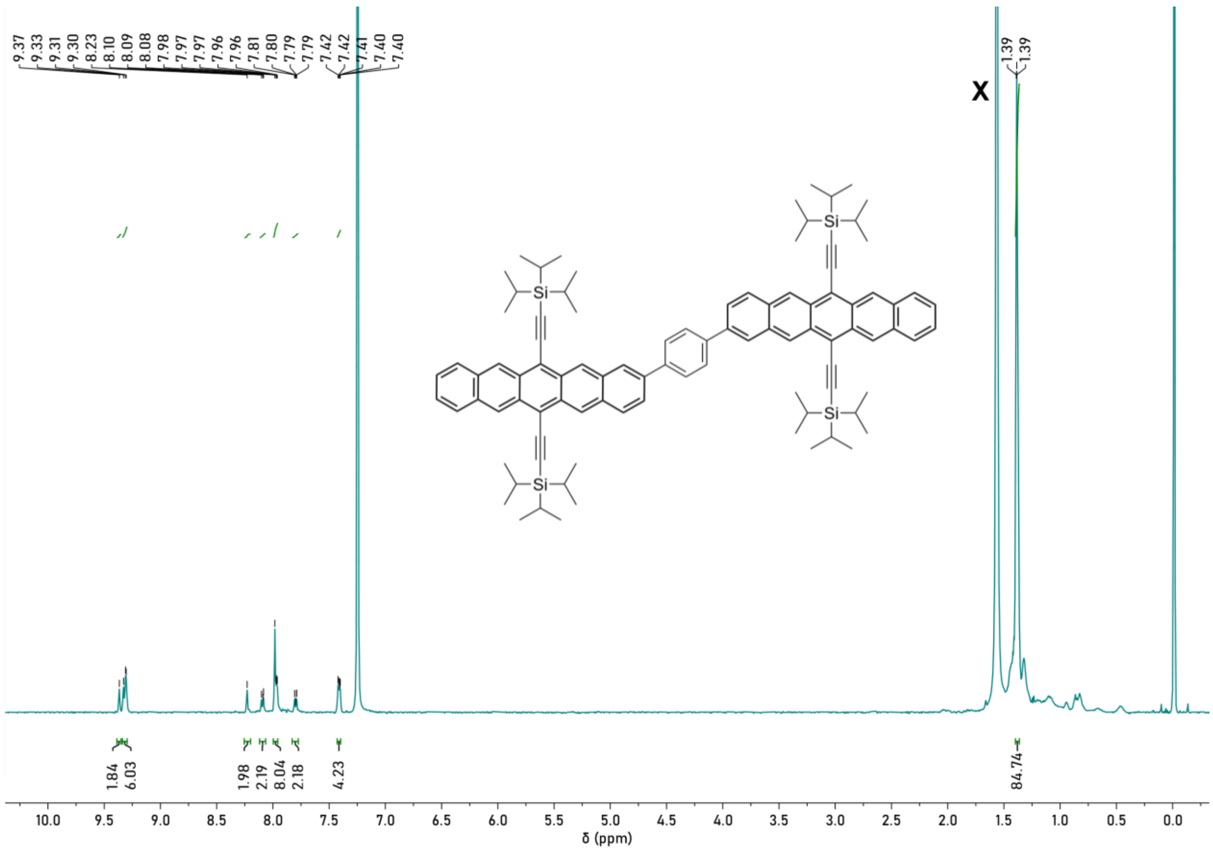

Figure S5.39. *^{1}H NMR spectrum of **2-P2Ph** in $CDCl_3$ [x = H_2O]*

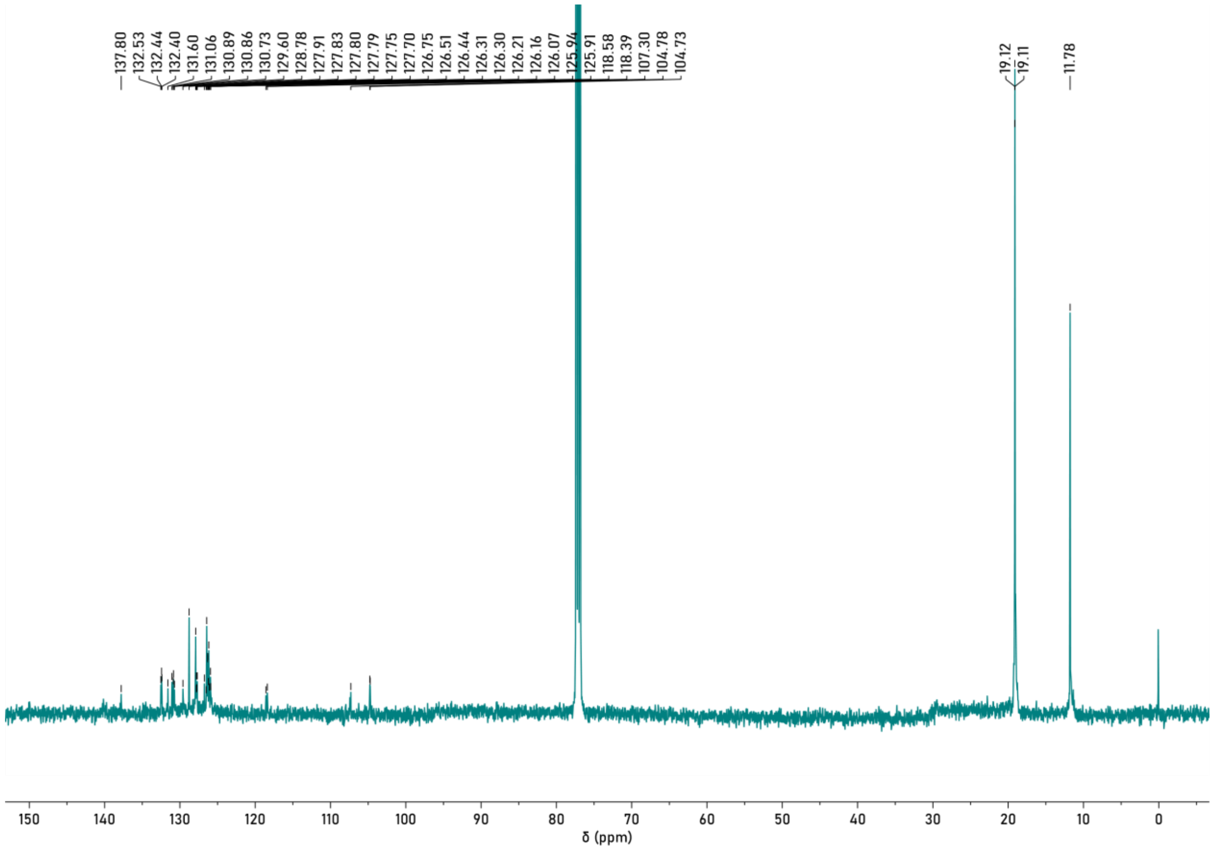

Figure S5.40. *^{13}C NMR spectrum of **2-P2Ph** in $CDCl_3$*

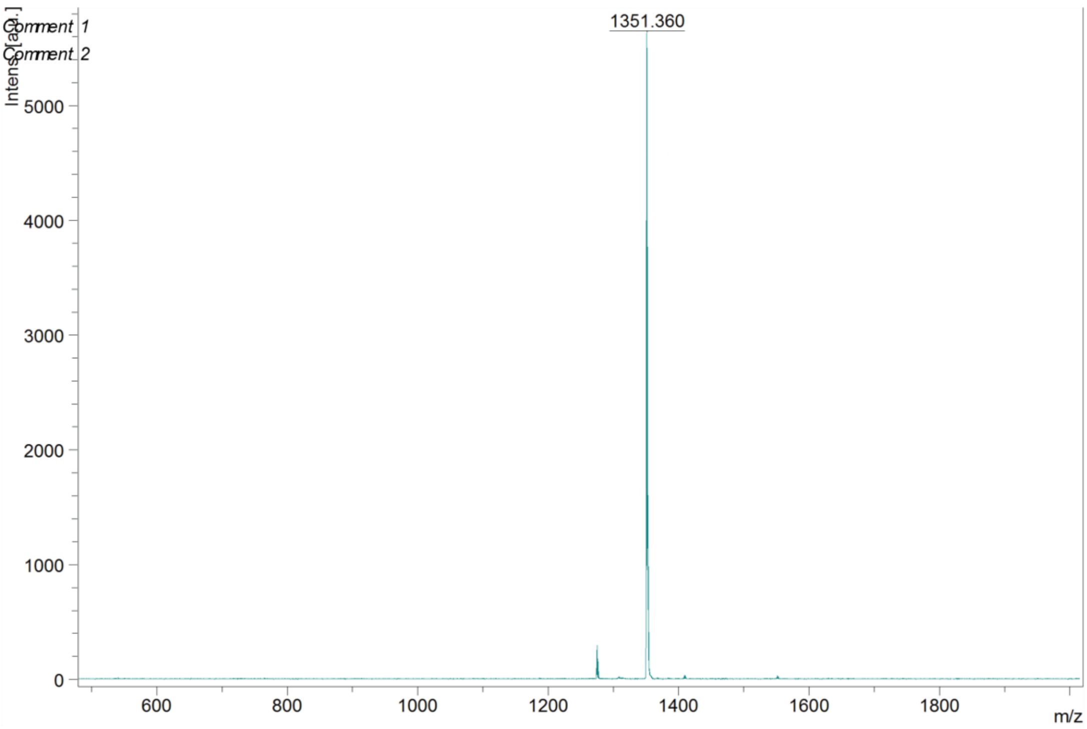

Acquisition Parameter

Date of acquisition	2021-09-28T12:55:26.813+05:30
Acquisition method name	D:\Methods\flexControlMethods\LP_700-2000_Da.par
Aquisition operation mode	Linear
Voltage polarity	POS
Number of shots	2000
Name of spectrum used for calibration	
Calibration reference list used	PeptideCalibStandard mono

Instrument Info

User	JNCASR
Instrument	ATS-00704
Instrument type	autoflex

Bruker Daltonics flexAnalysis printed: 9/28/2021 5:15:39 PM

Figure S5.41. *MALDI-MS of **2-P2Ph***

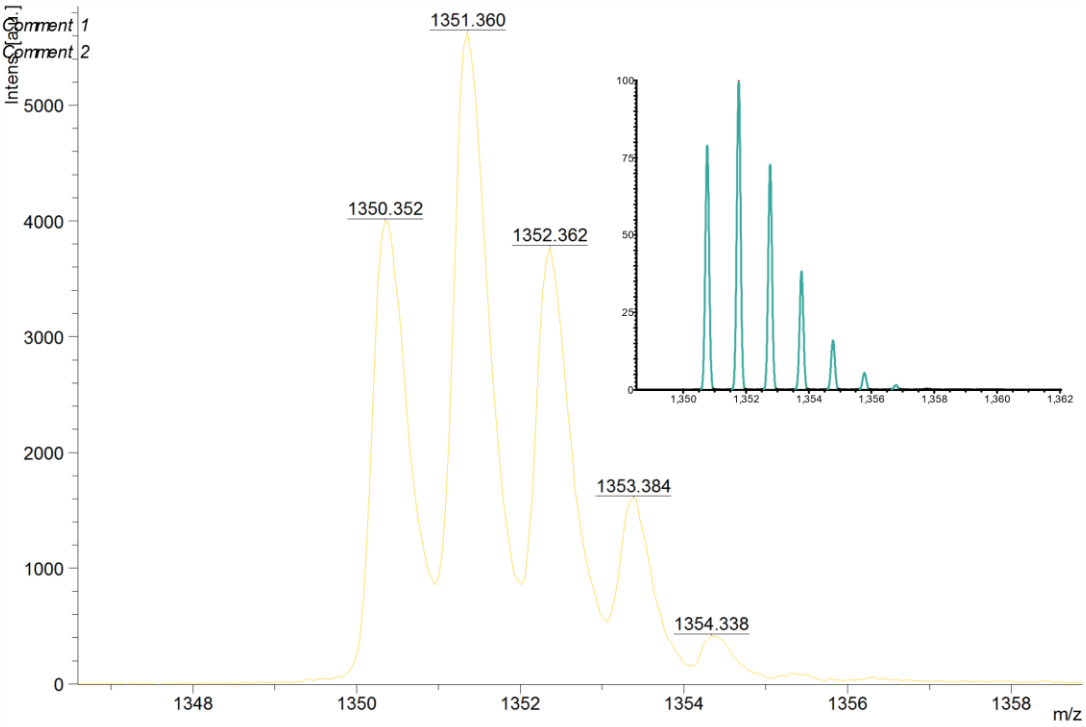

m/z	S/N	Quality Fac.	Res.	Intens.	Area
1350.352				3998	
1351.360	678		586	5632	8432
1352.362				3771	
1353.384				1605	
1354.338				413	

Acquisition Parameter

Date of acquisition	2021-09-28T12:55:26.813+05:30
Acquisition method name	D:\Methods\flexControlMethods\LP_700-2000_Da.par
Aquisition operation mode	Linear
Voltage polarity	POS
Number of shots	2000
Name of spectrum used for calibration	
Calibration reference list used	PeptideCalibStandard mono

Instrument Info

User	JNCASR
Instrument	ATS-00704
Instrument type	autoflex

Bruker Daltonics flexAnalysis printed: 9/5/2022 5:45:40 PM

Figure S5.42. *MALDI-MS isotopic distribution of **2-P2Ph**, (M^+). Simulated isotopic distribution (inset).*

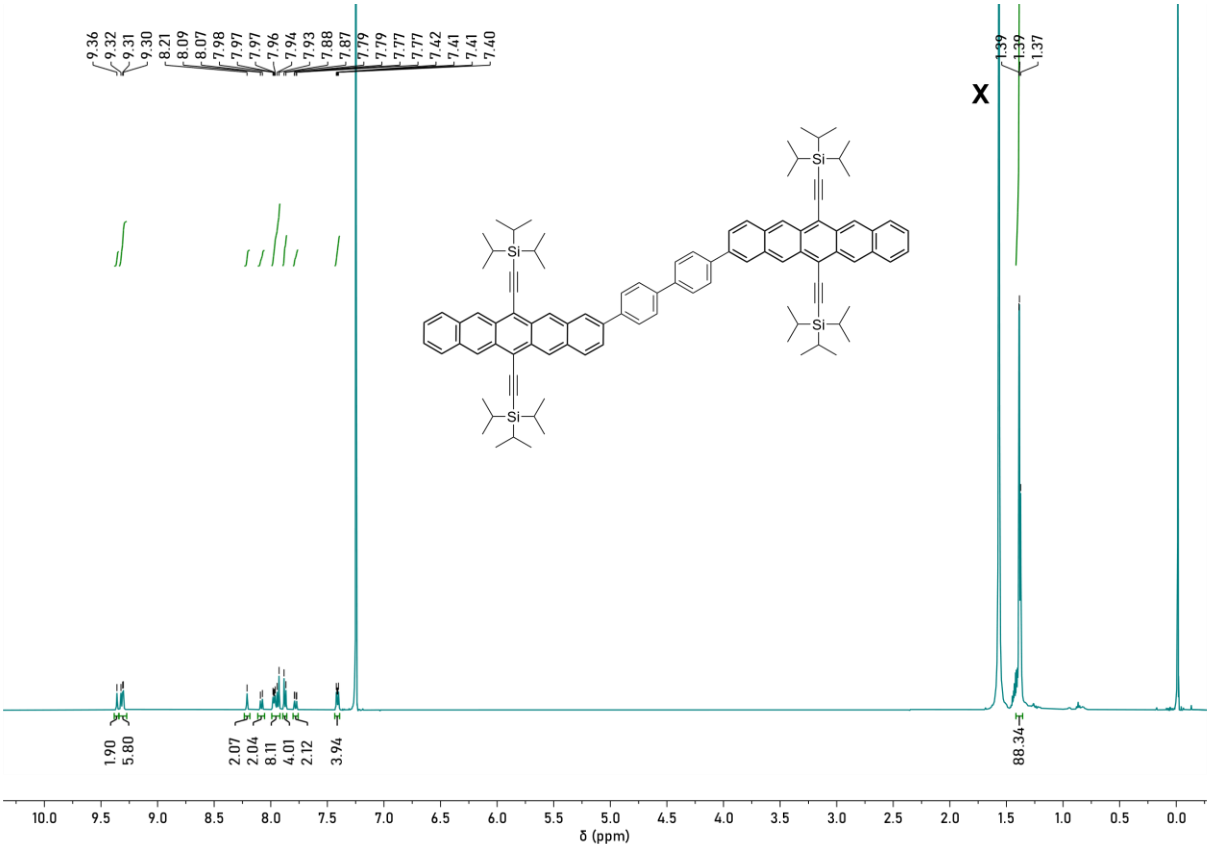

Figure S5.43. *^{1}H NMR spectrum of **2-P2BP** in $CDCl_3$ [x = H_2O]*

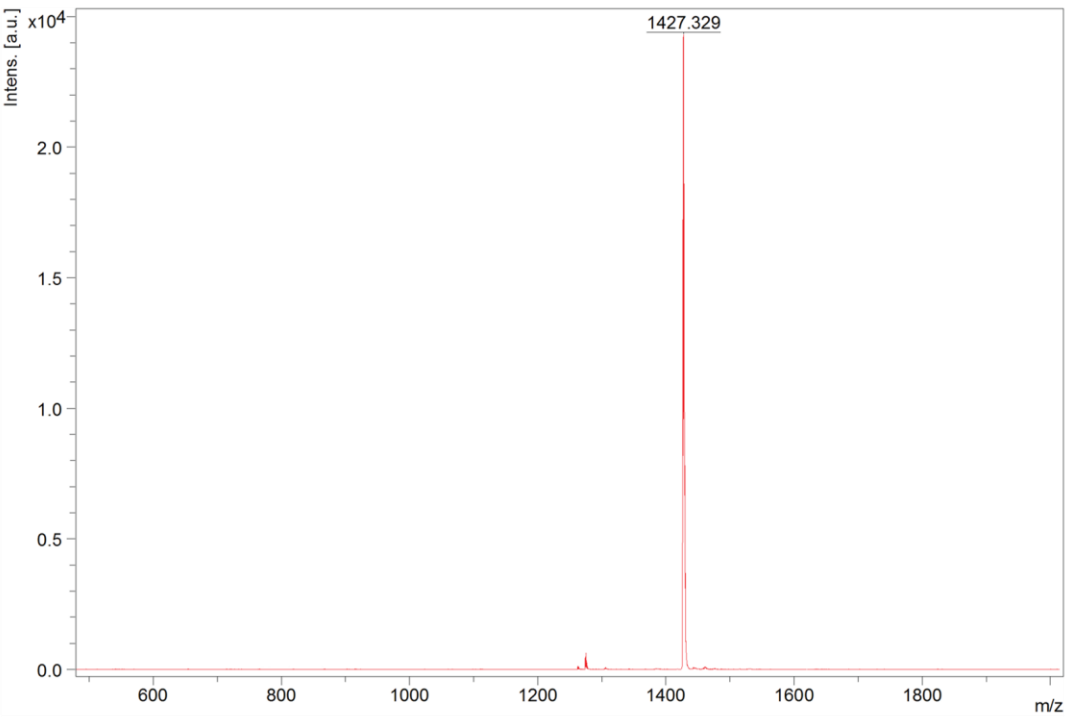

Acquisition Parameter

Date of acquisition	2021-09-28T12:53:26.989+05:30
Acquisition method name	D:\Methods\flexControlMethods\LP_700-2000_Da.par
Aquisition operation mode	Linear
Voltage polarity	POS
Number of shots	2000
Name of spectrum used for calibration	
Calibration reference list used	PeptideCalibStandard mono

Instrument Info

User	JNCASR
Instrument	ATS-00704
Instrument type	autoflex

Bruker Daltonics flexAnalysis printed: 9/28/2021 5:13:52 PM

Figure S5.44. *MALDI-MS of **2-P2BP***

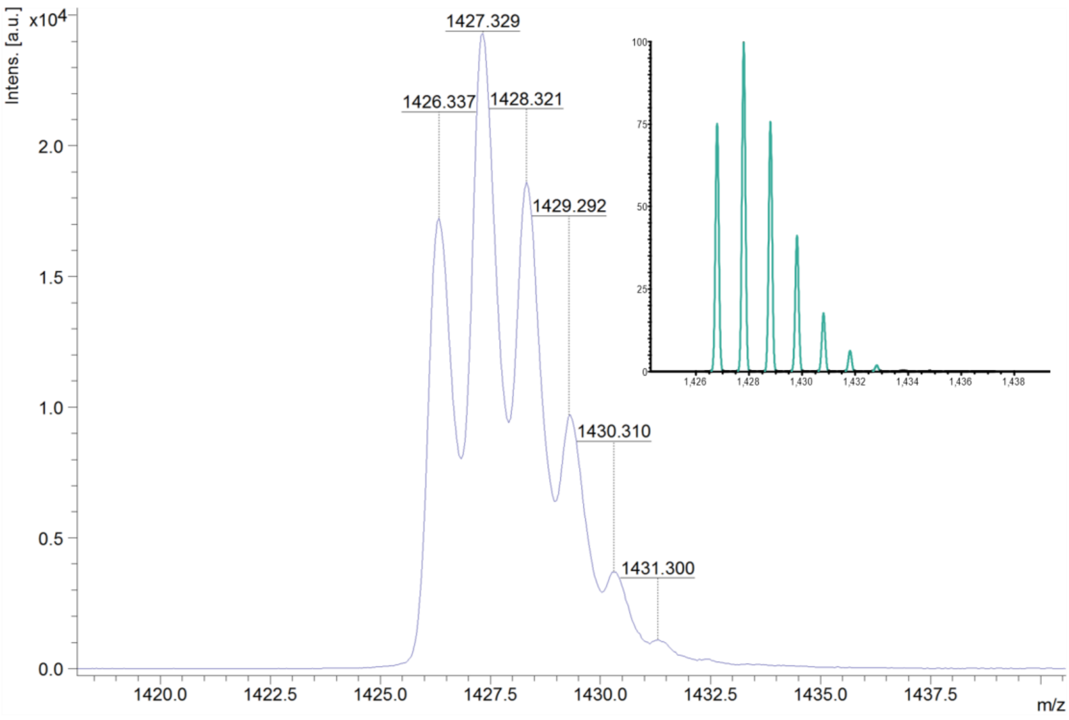

m/z	S/N	Quality Fac.	Res.	Intens.	Area
1426.337				17237	
1427.329	2667		576	24313	52861
1428.321				18614	
1429.292				9663	
1430.310				3686	
1431.300				1095	

Acquisition Parameter

Date of acquisition	2021-09-28T12:53:26.989+05:30
Acquisition method name	D:\Methods\flexControlMethods\LP_700-2000_Da.par
Aquisition operation mode	Linear
Voltage polarity	POS
Number of shots	2000
Name of spectrum used for calibration	
Calibration reference list used	PeptideCalibStandard mono

Instrument Info

User	JNCASR
Instrument	ATS-00704
Instrument type	autoflex

Bruker Daltonics flexAnalysis printed: 9/5/2022 5:41:53 PM

Figure S5.45. *MALDI-MS isotopic distribution of **2-P2BP**, (M^+). Simulated isotopic distribution (inset).*

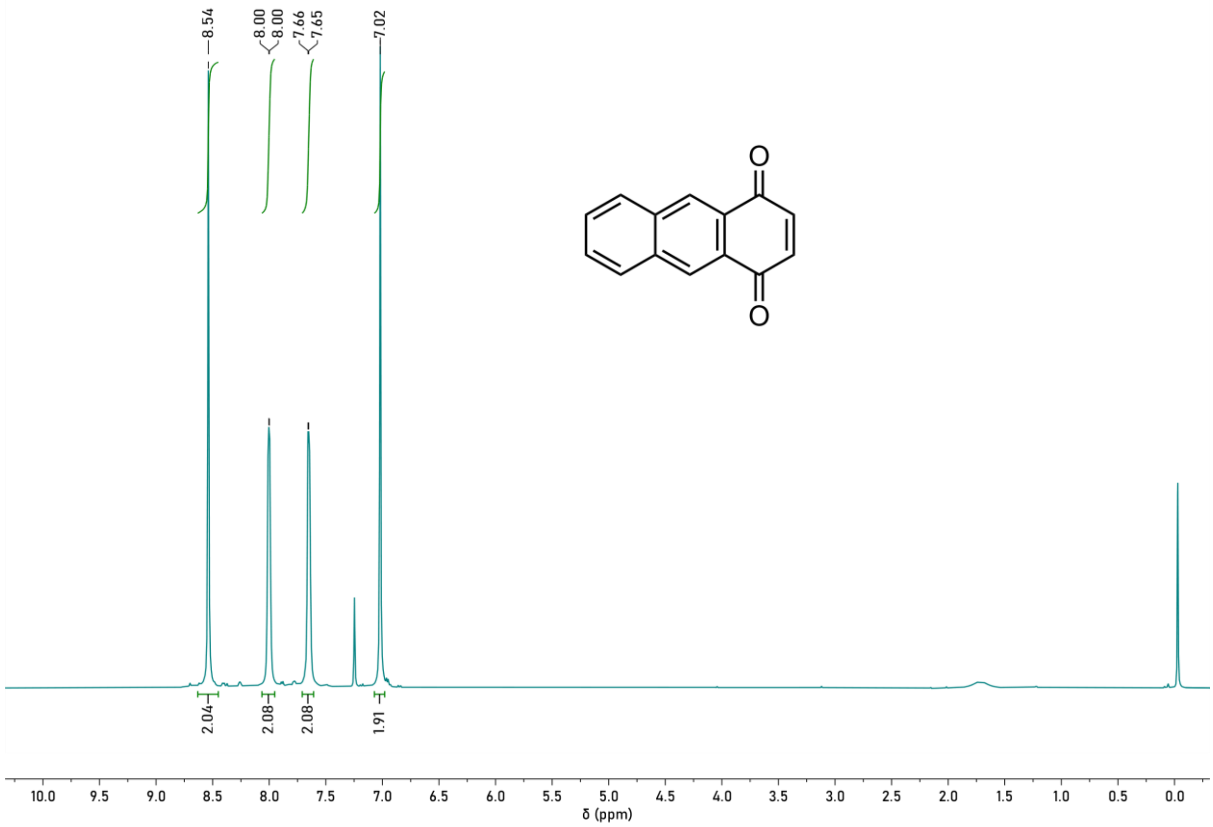

Figure S5.46. *^{1}H NMR spectrum of Anthracene 1,4 dione in $CDCl_3$ [x = H_2O]*

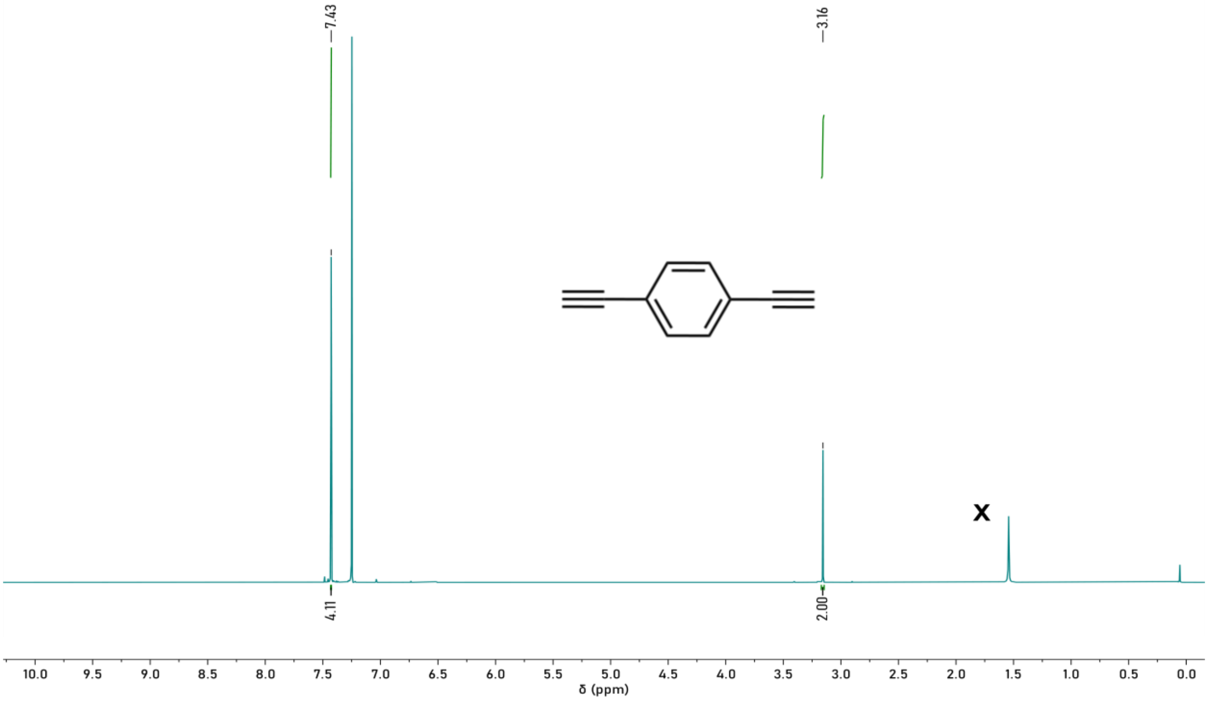

Figure S5.47. [1]*H NMR spectrum of* ***5b (n=1)*** *in* $CDCl_3$ *[x =* H_2O*]*

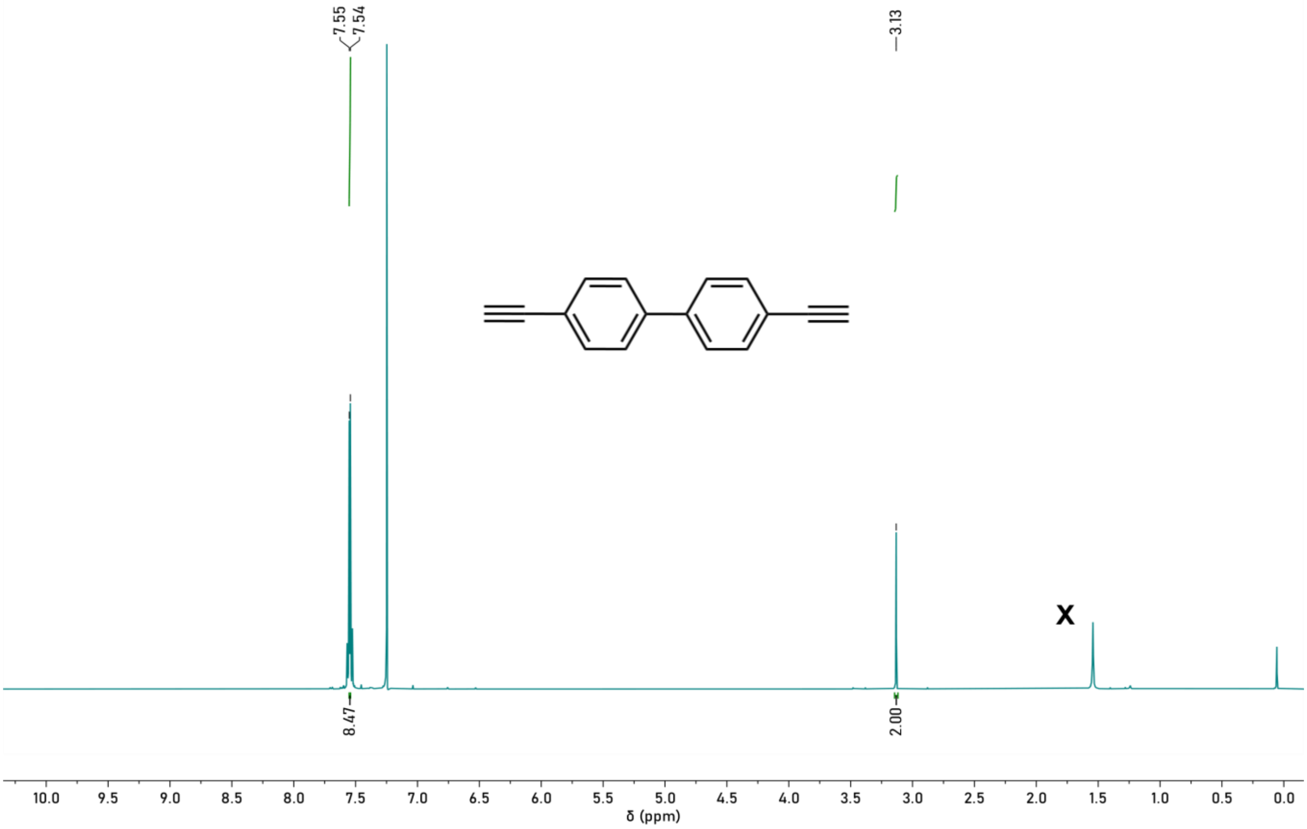

Figure S5.48. [1]*H NMR spectrum of* ***5b(n=2)*** *in* $CDCl_3$ *[x =* H_2O*]*

6. References

[1] M. J. et al. Frisch, *Gaussian 16 Rev. C.01; Wallingford, CT,* **n.d.**

[2] T. Yanai, D. P. Tew, N. C. Handy, *Chem. Phys. Lett.* **2004**, *393*, 51–57.

[3] G. A. Petersson, A. Bennett, T. G. Tensfeldt, M. A. Al- Laham, W. A. Shirley, J. Mantzaris, *J. Chem. Phys.* **1988**, *89*, 2193–2218.

[4] S. N. Sanders, E. Kumarasamy, A. B. Pun, M. T. Trinh, B. Choi, J. Xia, E. J. Taffet, J. Z. Low, J. R. Miller, X. Roy, X.-Y. Zhu, M. L. Steigerwald, M. Y. Sfeir, L. M. Campos, *J. Am. Chem. Soc.* **2015**, *137*, 8965–8972.

[5] K. N. Plunkett, K. Godula, C. Nuckolls, N. Tremblay, A. C. Whalley, S. Xiao, *Org. Lett.* **2009**, *11*, 2225–2228.

[6] N. Vets, M. Smet, W. Dehaen, *Tetrahedron Lett.* **2004**, *45*, 7287–7289.

[7] D. Lehnherr, A. H. Murray, R. McDonald, R. R. Tykwinski, *Angew. Chemie Int. Ed.* **2010**, *49*, 6190–6194.

[8] E. G. Fuemmeler, S. N. Sanders, A. B. Pun, E. Kumarasamy, T. Zeng, K. Miyata, M. L. Steigerwald, X.-Y. Zhu, M. Y. Sfeir, L. M. Campos, N. Ananth, *ACS Cent. Sci.* **2016**, *2*, 316–324.

[9] L. M. Yablon, S. N. Sanders, K. Miyazaki, E. Kumarasamy, G. He, B. Choi, N. Ananth, M. Y. Sfeir, L. M. Campos, *Mater. Horizons* **2022**, *9*, 462–470.
